# Current practice of usual clinic blood pressure measurement in people with and without diabetes: a survey and prospective ‘mystery shopper’ study in UK primary care

**DOI:** 10.1136/bmjopen-2017-020589

**Published:** 2018-04-12

**Authors:** Sarah L Stevens, Richard J McManus, Richard John Stevens

**Affiliations:** Nuffield Department of Primary Care Health Sciences, University of Oxford, Oxford, UK

**Keywords:** primary care, hypertension, general diabetes, quality in health care

## Abstract

**Objectives:**

Hypertension trials and epidemiological studies use multiple clinic blood pressure (BP) measurements at each visit. Repeat measurement is also recommended in international guidance; however, little is known about how BP is measured routinely. This is important for individual patient management and because routinely recorded readings form part of research databases. We aimed to determine the current practice of BP measurement during routine general practice appointments.

**Design:**

(1) An online cross-sectional survey and (2) a prospective ‘mystery shopper’ study where patients agreed to report how BP was measured during their next appointment.

**Setting:**

Primary care.

**Participants:**

Patient charity/involvement group members completing an online survey between July 2015 and January 2016. 334 participants completed the prospective study (51.5% male, mean age=59.3 years) of which 279 (83.5%) had diabetes.

**Primary outcome:**

Proportion of patients having BP measured according to guidelines.

**Results:**

217 participants with (183) and without diabetes (34) had their BP measured at their last appointment. BP was measured in line with UK guidance in 63.7% and 60.0% of participants with and without diabetes, respectively. Initial pressures were significantly higher in those who had their BP measured more than once compared with only once (p=0.016/0.089 systolic and p<0.001/p=0.022 diastolic, in patients with/without diabetes, respectively).

**Conclusions:**

Current practice of routine BP measurement in UK primary care is often concordant with guidelines for repeat measurement. Further studies are required to confirm findings in broader populations, to confirm when a third repeat reading is obtained routinely and to assess adherence to other aspects of BP measurement guidance.

**Strengths and limitations of this study**

- This survey has a novel ‘mystery shopper’ design, minimising biases that may be introduced by self-reported practitioner behaviour.
- We have examined how adherence to guidelines varies according to patient characteristics, whereas previous studies have taken a healthcare professional view.
- The use of an online survey may have resulted in an under-representation of some groups, such as the very elderly.
- Larger studies are required to confirm our findings with respect to second and third blood pressure readings.

## Introduction

Measurement of blood pressure (BP) is carried out in general practice by healthcare professionals on a daily basis. Such measurement is important for the diagnosis and management of hypertension, a major risk factor for cardiovascular disease (CVD) in both the general population^1^ and even more so in those with diabetes.^2^ Hypertension trials and major epidemiological studies typically measure clinic BP using strict protocols on two to three times per visit in most cases.^3^ For example, the Systolic Blood Pressure Interventon Trial (SPRINT)^4^ and the Action to Control Cardiovascular Risk in Diabetes (ACCORD)^5^ trial used the mean of three readings taken automatically to guide treatment. Repeated measurement protocols are also recommended in the UK,^6^ European^7^ and North American^8^ hypertension guidelines. For example, current UK guidance states that BP should be remeasured if it is initially high, or if two measurements differ substantially, with out-of-office monitoring recommended in those with sustained high BP in clinic.^6^ This reflects concerns that in many patients, clinic BP readings, particularly initial readings, may be systematically higher than BP during usual daily activities.^9^

Many factors can affect the accuracy of BP measurement and the number of measurements used can influence estimates of BP control.^10 11^ Measurement practices may also vary depending on the focus of the consultation or patient characteristics and recorded BPs may also be influenced by incentive schemes such as the UK Quality and Outcomes Framework.^12 13^ Potential differences between primary study protocols and clinical practice have implications for the generalisability and implementation of research findings. For example, SPRINT found that treatment to a systolic BP target of 120 mm Hg resulted in fewer cardiovascular events compared with a target of 140 mm Hg in low-risk patients.^4^ However, others have argued that mean automatically measured BP of 120 mm Hg may correspond to a routine measurement of 135–140 mm Hg^14^ and ACCORD (conducted in patients with diabetes) failed to show an effect of intensive treatment.^5^

Furthermore, increasing numbers of observational studies in electronic healthcare databases rely on routinely collected BP measurements. In particular, the recommended cardiovascular risk calculator in the UK, QRISK2^15^ was derived using such data. It is important to understand how BPs recorded in these databases were obtained, in order to reliably compare observational database and primary study results.

However, little is known about how BP is measured in routine practice. A 2006 survey of UK general practitioners’ (GPs’) adherence to hypertension guideline recommendations relied on self-reported data and did not ask about the use of repeat measurements.^16^ Other European studies have focused on whether implementation of lifestyle or treatment changes adheres to guidelines^17^ or reasons for non-adherence.^18^ These studies assume that an accurate BP reading is obtained initially and ignore the specifics of BP measurement. We, therefore, sought to determine the current practice of BP measurement during routine appointments in UK primary care, focusing on when repeat clinic and home BP measurements are obtained.

## Patients and methods

We conducted an online survey of patients, followed by a prospective survey of primary care consultations.

### Online survey

An online survey was advertised through charities and patient involvement groups (‘University of the Third Age’, ‘Blood Pressure UK’, ‘Citizen Scientist’, ‘Patients Active in Research’, ‘Call for Participants’ and ‘Research for the Future (Help BEAT Diabetes)’) between 23 July 2015 and 24 January 2016. Respondents anonymously reported basic demographic and health information, if and how many times their BP was measured at their last appointment and (recall permitting) their last BP reading (online supplementary material). Respondents were also asked about recommendations to monitor their BP at home.

10.1136/bmjopen-2017-020589.supp1 Supplementary file 1

### Prospective study

Participants completing the online survey were invited to take part in a prospective study. They were told the study would ask similar questions to those already asked about their BP after their next primary care appointment. Those wishing to take part gave explicit consent, provided an email address and were asked when they expected their next appointment to be. After the anticipated time of this appointment, a link to an online questionnaire was emailed to participants. This asked whether BP was measured at the appointment, and if so, how many times, and (recall permitting) for up to three systolic and diastolic BP values (online supplementary material). The questionnaire was open from 23 July 2015 to 16 June 2016. Two patient representatives helped design the study materials and three were asked to pilot the survey websites to test functionality.

### Statistical analysis

The prospective study was powered to estimate the proportion of people having their BP measured once or multiple times, in line with guidelines at the 95% confidence level with an accuracy of ±5%. Assuming a proportion of 10%, 139 respondents who had had their BP measured was required.^19^

Demographic and clinical history data were summarised using means and SD or proportions. Mean BP was summarised with 95% CIs and ranges. Respondents were classified as hypertensive if they answered yes to the question ‘Have you got high blood pressure or have you ever been told by your GP that you have high blood pressure?’. Responses were assessed against National Institute for Health and Care Excellence guidance and BP was deemed to have been measured according to guidelines if BP was measured: (1) once and the reading was below 140/90 mm Hg, (2) two times if the initial reading was above 140/90 mm Hg and the first two readings differed by less than 5 mm Hg systolic or (3) three times if the first reading was above 140/90 mm Hg and the first two readings differed by more than 5 mm Hg systolic.^6^ Respondents who had their BP measured more or less than guidance recommends were deemed not to have had their BP measured according to guidance. Proportions were compared using two-sided tests of proportions, under the assumption of large samples, at the 5% level. Due to an unexpectedly large proportion of participants with diabetes, a decision to stratify all prospective study analyses by patient diabetes status was made after data collection.

Since behaviour among professionals from the same practice may be similar, sensitivity analyses were carried out by randomly selecting one observation from each postal code district (assuming respondents from different districts are registered to distinct practices). We also conducted sensitivity analyses excluding prospective responses that were suspected of being duplicate submissions of the same initial survey data. Analysis was conducted using Stata V.14^20^ and R V.3.3.1.^21^

## Results

**Figure 1:**
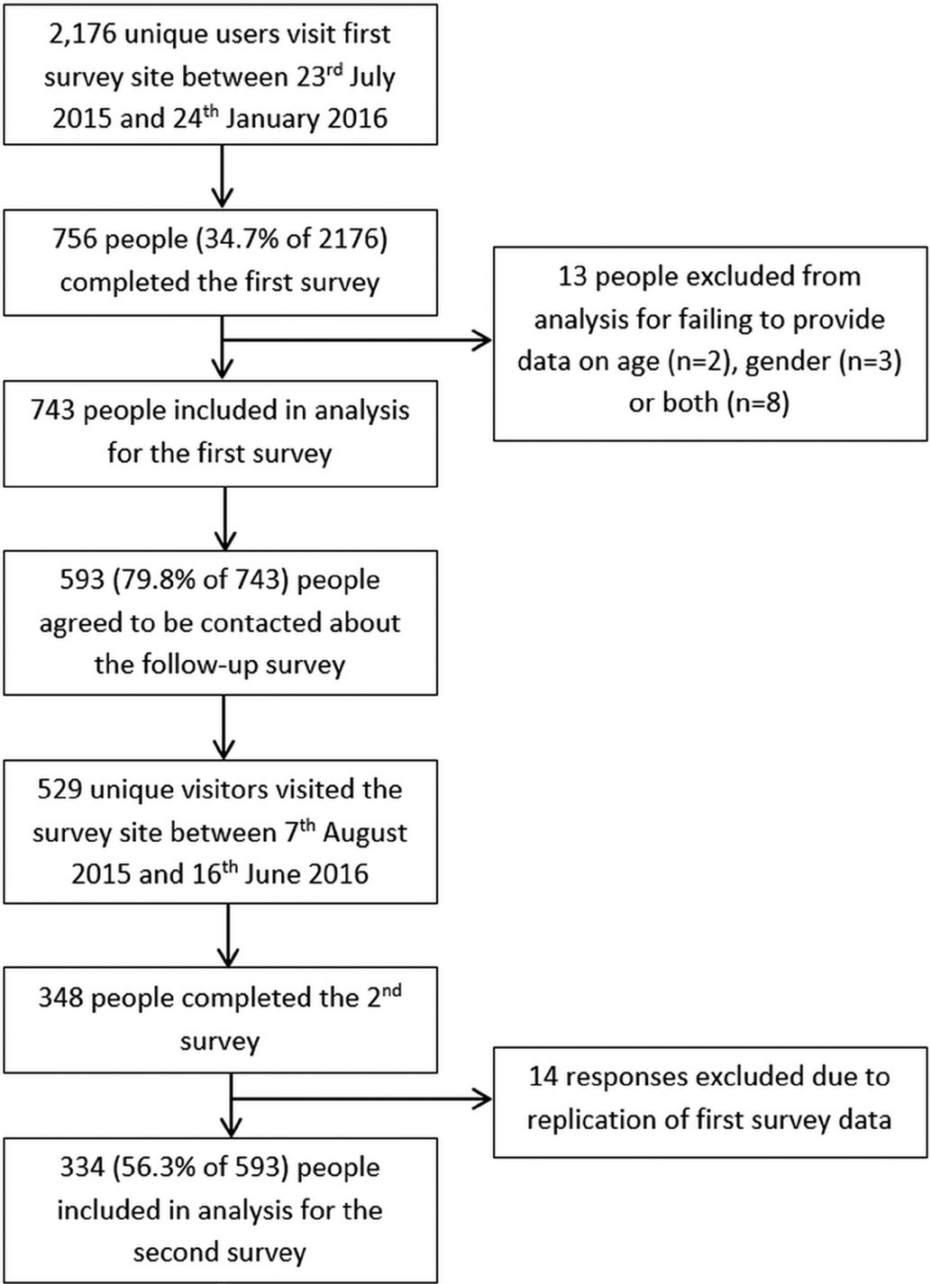
Study flow chart.

In total, 2176 unique users visited the survey site, of whom 756 completed the initial online survey, with complete data available in 743 individuals (623 with diabetes, 83.9%). Consent for the prospective study was given by 593 participants and was completed by 334 participants (279 with diabetes, 83.5%) (figure 1). The characteristics of those completing the initial and prospective surveys were broadly similar (online supplementary table S1).

### Initial survey

Of the 743 people completing the first survey, 489 (65.8%) reported having had their BP measured at their last appointment: 156 (31.9% of 489) by a GP, 321 (65.6%) by a nurse and 12 (2.5%) in the waiting room. Most respondents (480/489, 98.2%) could recall how many BP readings were taken: 286 (59.6% of 480) one, 144 (30.0%) two and 50 (10.4%) three or more readings. Results stratified by diabetes status are given in the online supplementary tables S2 and S3. Only 88 patients (11.8%) recalled ever having their BP measured in both arms at any one previous appointment. Compared with normotensives (20/330, (6.7%)), respondents with a previous diagnosis of hypertension (68/413, (16.5%)) were more likely to report having had their BP measured in both arms at any appointment previously.

### Prospective study

Baseline characteristics for those with and without diabetes completing the prospective study after a further GP appointment are given in table 1. Of the 279 participants with diabetes completing the follow-up questionnaire, 183 (65.6%) had their BP measured at the appointment: 38 (20.8%) by a GP, 139 (76.0%) by a nurse and 6 (3.3%) by themselves in the waiting room. Of the 55 participants without diabetes, 34 (61.8%) had their BP measured: 21 (61.8%) by a GP, 11 (32.4%) by a nurse and 2 (5.9%) by themselves in the waiting room.

**Table 1:** Characteristics of participants completing the prospective survey with and without diabetes

Characteristic	Participants with diabetes (n=279)	Participants without diabetes (n=55)
	Mean (SD)/n (%)	Mean (SD)/n (%)
Male	157 (56.3)	15 (27.3)
Age	59.0 (12.1)	60.3 (12.7)
Current smoker	21 (7.5)	4 (7.3)
Hypertensive	159 (57.0)	41 (74.6)
Antihypertensive medication	141 (88.7)	32 (78.0)
Previous CVD	29 (10.4)	2 (3.6)
Chronic kidney disease	11 (3.9)	1 (1.8)
Rheumatoid arthritis	12 (4.3)	1 (1.8)
Told at high risk of CVD	26 (9.3)	4 (7.3)
Region		
Northeast	9 (3.2)	0 (0.0)
Northwest	111 (39.8)	14 (25.5)
Yorkshire and The Humber	19 (6.8)	1 (1.8)
East Midlands	6 (2.2)	2 (3.6)
West Midlands	13 (4.7)	3 (5.5)
East of England	22 (7.9)	6 (10.9)
Southwest	40 (14.3)	9 (16.4)
Southeast	42 (15.1)	15 (27.3)
London	13 (4.7)	2 (3.6)
Other	0 (0.0)	2 (3.6)
Unknown	4 (1.4)	1 (1.8)

CVD, cardiovascular disease.

#### Participants with diabetes

**Figure 2:**
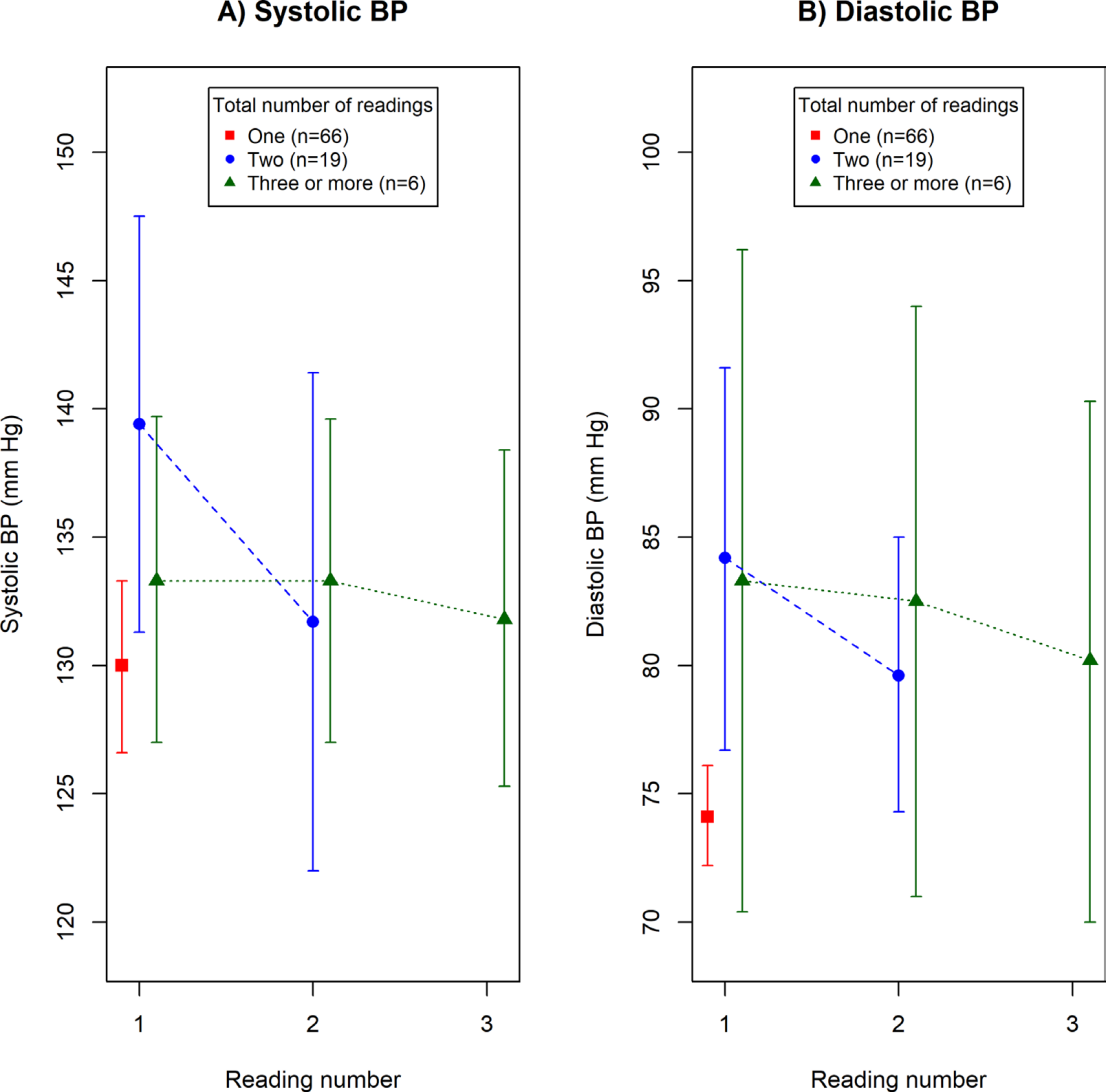
Mean blood pressure (BP) and 95% CI by reading number in 91 participants with diabetes who reported a value for each BP reading in the prospective survey.

Of the 183 participants with diabetes who had their BP measured, 91 (49.7%) could recall a value for all of the BP readings given. Fifty-eight respondents (63.7%, 95% CI 53.0% to 73.6%) had their BP measured according to guidelines. Mean BP values by reading number are presented graphically in figure 2A (systolic) and figure 2B (diastolic, see online supplementary table S4 for raw data). Initial systolic and diastolic BPs were lower in participants who had their BP measured only once than in those who had it measured two or more times (mean systolic difference=8.0 mm Hg, 95% CI 1.2 to 14.5 mm Hg, p=0.016 and mean diastolic difference=9.9 mm Hg, 95% CI 5.1 to 14.6 mm Hg, p<0.001).

The proportion of participants with diabetes who had their BP measured multiple times was similar regardless of hypertensive or treatment status, or measurement personnel (table 2, top left). However, they were more likely to be asked to monitor their BP at home when BP was measured by a GP compared with a nurse (table 2, top right). Those who had their BP measured once, two and three or more times, were asked to monitor their BP at home in 10/109 (9.2%, 95% CI 3.8% to 14.6%), 11/51 (21.6%, 95% CI 10.3% to 32.9%) and 7/23 (30.4%, 95% CI 11.6% to 49.2%) cases, respectively.

**Table 2:** Likelihood of having BP measured multiple times or being asked to monitor BP at home, according to patient and practitioner characteristics (stratified by diabetes status)

	Likelihood of multiple BP measurements (n (%) in each group) (difference (95% CI))	Likelihood of being asked to monitor BP at home (n (%) in each group) (difference (95% CI))
In participants with diabetes		
If the participant was hypertensive versus normotensive	46/103 (44.7%) vs 28/80 (35.0%), difference=9.7% (−4.5% to 23.9%)	24/159 (15.1%) vs 13/120 (10.8%), difference=4.3% (−3.6% to 12.1%)
If the participant had treated hypertension versus untreated hypertension	40/93 (43.0%) vs 6/10 (60.0%), difference=−17.0% (−49.0% to 15.0%)	22/141 (15.6%) vs 2/18 (11.1%), difference=4.5% (−11.2% to 20.2%)
If BP was measured by a GP versus a nurse	16/38 (42.1%) vs 56/139 (40.3%), difference=1.8% (−15.9% to 19.5%)	11/38 (28.9%) vs 15/139 (10.8%), difference=18.2% (2.8% to 33.5%)
In participants without diabetes		
If the participant was hypertensive versus normotensive	2/6 (33.3%) vs 9/28 (32.1%), difference=1.2% (−40.3% to 42.7%)	14/41 (34.1%) vs 0/14 (0.0%), difference=34.1% (19.6% to 48.7%)
If the participant had treated hypertension versus untreated hypertension	8/23 (34.8%) vs 1/5 (20.0%), difference=14.8% (−25.3% to 54.9%)	9/32 (28.1%) vs 5/9 (55.6%), difference=−27.4% (−63.4% to 8.6%)
If BP was measured by a GP versus a nurse	9/21 (42.9%) vs 2/11 (18.2%), difference=24.7% (−6.4% to 55.8%)	7/21 (33.3%) vs 4/11 (36.4%), difference=−3.0% (−37.9% to 31.8%)

BP, blood pressure; GP, general practitioner.

#### Participants without diabetes

**Figure 3:**
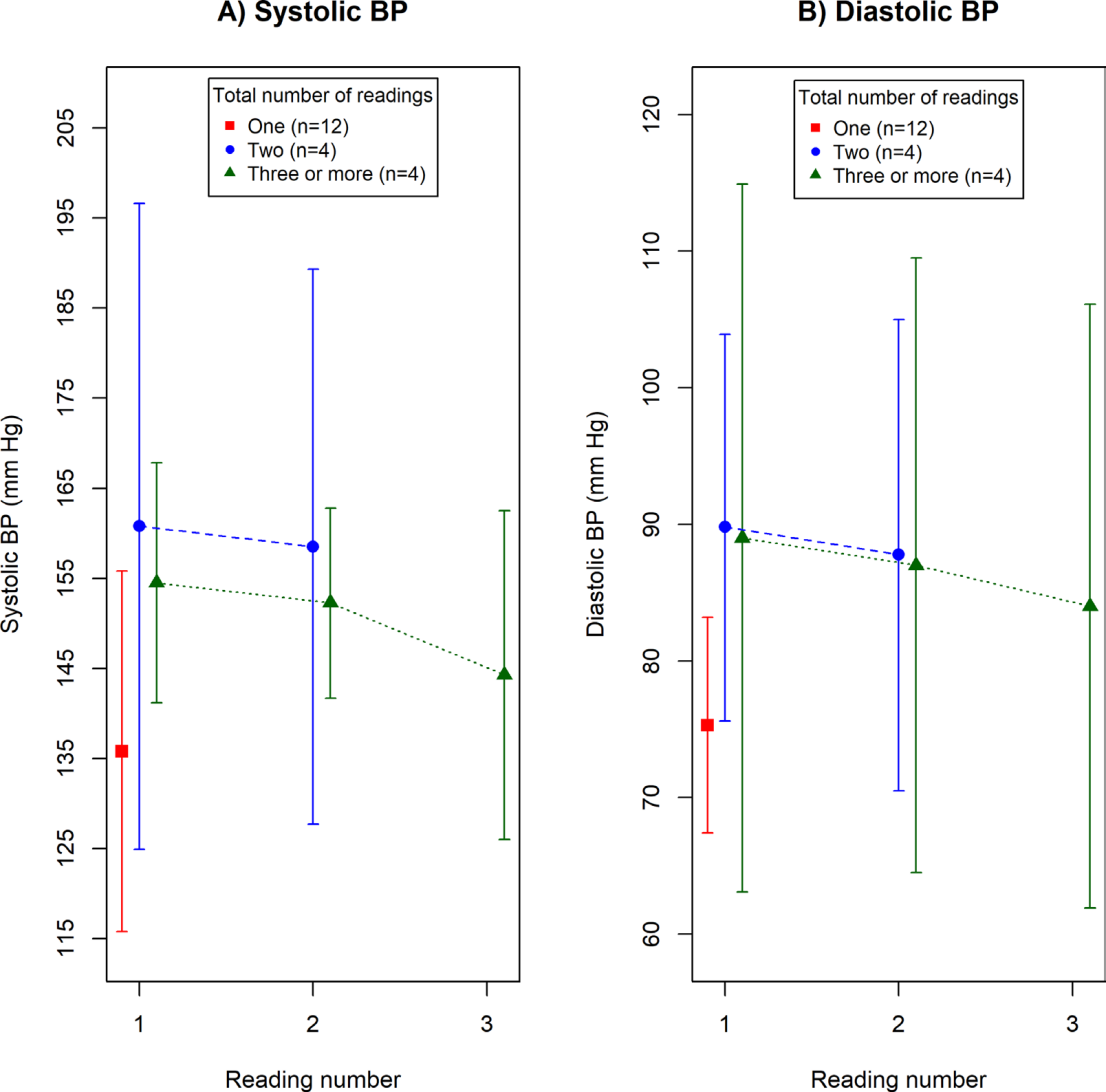
Mean blood pressure (BP) and 95% CIs by reading number in 20 participants without diabetes who reported a value for each BP reading in the prospective survey.

Of the 34 participants without diabetes who had their BP measured, 20 (58.8%) could recall a value for all of the BP readings given. Twelve respondents (60.0%, 95% CI 36.1% to 80.9%) had their BP measured according to guidelines. Mean BP values by reading number are presented graphically in figure 3A (systolic) and figure 3B (diastolic, see online supplementary table S5 for raw data). Patterns of repeat BP measurement were similar to those observed in participants with diabetes, although numbers in this group were smaller. Initial systolic BPs were non-significantly lower in participants who had their BP measured only once than in those who had it measured two or more times (mean systolic difference=21.8 mm Hg, 95% CI −3.7 to 47.3 mm Hg, p=0.089). However, a significant difference was observed for diastolic pressure (mean diastolic difference=14.1 mm Hg, 95% CI 2.3 to 26.0 mm Hg, p=0.022).

The proportion of participants without diabetes who had their BP measured multiple times was similar regardless of hypertensive or treatment status, or measurement personnel (table 2, bottom left). However, those with hypertension were more likely to be asked to monitor their BP at home compared with normotensives (table 2, bottom right). Those who had their BP measured once, two and three or more times were asked to monitor their BP at home in 5/23 (21.7%, 95% CI 4.9% to 38.6%), 3/5 (60.0%, 95% CI 17.1% to 100%) and 3/6 (50.0%, 95% CI 10.0% to 90.0%) cases, respectively.

### Sensitivity analyses

Results were similar after randomly sampling responses from unique postal code districts (online supplementary tables S6–S8) or when excluding prospective responses suspected of being duplicate submissions of the initial survey data (online supplementary tables S9–S11).

## Discussion

### Summary

This study has shown that a second BP measurement at clinic visit is more likely to be taken if the initial BP measurement is high. This is consistent with UK guidelines. However, there is no clear evidence that the decision to take a third measurement follows guidelines. The recommendation that a third measurement be taken only when the first two are discrepant (first measurement above threshold but second below threshold for diagnosis of hypertension) was not obviously reflected in our data, although CIs are wide. Although the majority of this evidence relates to people with diabetes, similar BP measurement practices were observed in those without diabetes.

### Strengths and limitations

The patient-centred nature of this study has allowed us to see into the consulting room for the first time and to determine how BP is measured in ‘real life’, in those with and without diabetes. Previous studies have taken a healthcare professional view.^16^

Our online survey was limited by the use of convenience mechanisms for recruitment, and like many internet surveys with no known denominator, these results should be interpreted with caution. The use of an online system itself may have resulted in an under-representation of some groups, such as the very elderly.^22^ For the prospective study, we were able to obtain ‘mystery shopper’ type data on more than 200 GP and nurse appointments without potentially influencing the appointment through direct observation by a researcher. To our knowledge, these data are unique. The lower numbers of respondents without diabetes could limit generalisability if healthcare professionals follow protocols less carefully in patients without additional cardiovascular risk factors.^23^ Recruitment through patient involvement groups may have also resulted in over-representation of patients who are actively engaged with their healthcare, and due to phenomenon such as the inverse-care law,^24^ may receive better quality (guideline adherent) care. However, since our aim was to study the behaviours of healthcare professionals, it is unclear how any biases at the patient level will have translated into biases at the healthcare professional level. Furthermore, previous research regarding current practice of BP self-monitoring showed similar results using both convenience and representative sampling of professionals.^25^

Although our mechanism of data collection, asking patients to study the behaviour of their healthcare practitioners, has the limitations discussed above, we chose our ‘mystery shopper’ approach over several other study designs. For a full discussion of the study designs considered, see Stevens PhD thesis.^26^ Briefly, studies based on alternative methodologies, such as practitioner self-report or direct observation, would have been subject to selection bias among practitioners, the Hawthorne effect and reporting bias and we have avoided these biases through our novel design.

Self-reported BP readings may have been subject to rounding error, digit preference or recall error. This introduces uncertainty into some analyses concerning BP values, but the number of measurements taken, is likely to be recalled with greater accuracy, especially in the prospective study. Guidance covers many factors affecting the accuracy of BP measurement, such as the use an appropriately sized cuff, but such factors are less easily assessed by patients and we chose to limit the focus of this study in order to maximise response rates. The type and accuracy of devices used in UK general practice has been studied previously,^27^ but further direct observation of clinicians is warranted to determine if other aspects of BP measurement guidance is followed.

Fewer than anticipated participants provided all BP readings and therefore we could only estimate the proportion of people with diabetes having their BP measured according to guidelines with an error of ±10% (compared with an original target of ±5%). However, we have demonstrated important differences (eg, in first systolic BP readings) despite this. Although we have demonstrated that BP is measured in line with guidance in the majority of cases, this was driven by a large number of participants with low BP who had their BP measured only once. Larger studies would be required to confirm our findings, particularly with respect to second and third readings and in those without diabetes.

Many factors, other than the initial BP value, can influence the decision to measure BP multiple times including previous measurement of BP in clinic or at home and the presence of CVD or cardiovascular risk factors. Such factors may explain the considerable variability in BP measurement practices observed in some specific patient examples. Although we have addressed key factors such as diabetes, hypertension and treatment status, future research could explore behaviour in other subgroups. Furthermore, we did not ask respondents about the primary reason for their consultation which may have influenced BP measurement and this also requires further study.

### Comparison with existing literature

A previous review^18^ of barriers to hypertension awareness and treatment found that professionals were concerned about the accuracy of individual clinic BP readings. Our results support the idea that professionals treat single readings with caution, particularly those above the diagnostic threshold which require further action (eg, in the form of treatment change). Although numbers were smaller, results suggest that this caution also extends to high BP sustained over two readings. Previous research suggests that recording of BP may be influenced by specific BP-related targets in the UK’s Quality and Outcomes Framework,^12^ and hence routine practice in other healthcare systems, with different incentive schemes, may differ. Despite agreement between current practice and guidelines, GPs may be better advised to use multiple readings more widely^28^ to ensure comparability with BP monitoring studies and detection of masked hypertension which affects approximately 19% of adults.^29^

The finding that patients are more likely to be monitored at home if they have high clinic pressures or hypertension is consistent with results from a recent practitioner survey in Canada,^30^ where guidance is similar.^31^ A recent survey of general practices in the Southwest of England found that only 1 in 10 GP practices were not following current guidelines for the use of home and ambulatory BP monitoring in the diagnosis of hypertension,^32^ which is also consistent with our results in that guidance appears to be followed in most cases.

GPs were more likely to recommend home monitoring than nurses in those with diabetes. It is difficult to interpret this finding as it may reflect the primary reason for consultation, with certain tasks (such as diabetes reviews) performed primarily by nurses. Current guidance for BP management in diabetes recommends that high BP is confirmed at subsequent appointments, rather than through home monitoring.^33^ Hence, this finding, which importantly was not replicated in those without diabetes, may be explained if many of those with diabetes had annual review appointments. Overall, few patients were encouraged to monitor their BP at home, although it is likely that around 31% of patients were already self-monitoring based on previous UK survey data.^34^

### Implications for research and practice

Our findings indicate that routine BP measurement does not reflect the strict measurement protocols in primary research studies. This has implications for patient care if results from primary research studies cannot be appropriately translated into guidance for routine care (eg, in the form of adjusted treatment targets). Users of electronic healthcare databases should also be aware of the potential for recording biases^12^ which may dilute the observed effect of BP on outcomes and may extend to other biological factors subject to measurement error.

The current practice of BP measurement will, reassuringly, detect white coat hypertension but may not identify those with masked effects (where BP is higher outside of the clinic). This could potentially result in missed diagnoses and suboptimal treatment. One solution which would not increase workload is the use of the Predicting Out-of-Office Blood Pressure (PROOF-BP) tool which was developed by two of the authors with colleagues.^35^ This combines factors associated with home-clinic BP differences with BP readings to identify which patients may exhibit masked or white coat effects and would benefit most from out-of-office monitoring. It accurately identifies hypertension in 93% of cases and is more accurate than current diagnostic guidelines.^36^ Implementation of this tool could improve detection of masked effects and avoid unnecessary out-of-office monitoring.

Less than one in five participants with hypertension reported having their BP measured in both arms at a single appointment previously. Large differences between arms are associated with vascular disease and mortality.^37^ These results suggest little change since 13% of GPs said they adhered to this recommendation a decade ago.^16^ Other recent estimates suggest that around half of practices measure BP in both arms as part of the diagnostic procedure,^32^ which, although more optimistic, further demonstrates room for improvement. Barriers to such improvement may include practitioner discordance with guidance (previously only 30% agreed with the recommendation) or a lack of a suitable devices.^38^

The results of this study provide a preliminary insight into how BP is measured routinely and indicate that repeat BP measurements are taken in line with guidelines but not with strict study protocols. The impact of these differences on patient care requires further investigation.

## Supplementary Material

Reviewer comments

Author's manuscript

## References

[1] Lewington S, Clarke R, Qizilbash N, et al Prospective Studies Collaboration. Age-specific relevance of usual blood pressure to vascular mortality: a meta-analysis of individual data for one million adults in 61 prospective studies. Lancet 2002;360:1903–13. 12493255 10.1016/s0140-6736(02)11911-8

[2] Adler AI, Stratton IM, Neil HA, et al Association of systolic blood pressure with macrovascular and microvascular complications of type 2 diabetes (UKPDS 36): prospective observational study. BMJ 2000;321:412–9. 10.1136/bmj.321.7258.412 10938049 PMC27455

[3] Giorgini P, Weder AB, Jackson EA, et al A review of blood pressure measurement protocols among hypertension trials: implications for "evidence-based" clinical practice. J Am Soc Hypertens 2014;8:670–6. 10.1016/j.jash.2014.07.024 25224868

[4] Wright JT, Williamson JD, Whelton PK, et al A Randomized Trial of Intensive versus Standard Blood-Pressure Control. N Engl J Med 2015;373:2103–16. 10.1056/NEJMoa1511939 26551272 PMC4689591

[5] Cushman WC, Evans GW, Byington RP, et al Effects of intensive blood-pressure control in type 2 diabetes mellitus. N Engl J Med 2010;362:1575–85. 10.1056/NEJMoa1001286 20228401 PMC4123215

[6] National Institute for Health and Care Excellence. Hypertension: Clinical management of primary hypertension in adults (NICE CG127). London: National Institute for Health and Care Excellence, 2011.

[7] Mancia G, Fagard R, Narkiewicz K, et al 2013 ESH/ESC guidelines for the management of arterial hypertension: the Task Force for the Management of Arterial Hypertension of the European Society of Hypertension (ESH) and of the European Society of Cardiology (ESC). Eur Heart J 2013;34:2159–219. 10.1093/eurheartj/eht151 23771844

[8] James PA, Oparil S, Carter BL, et al 2014 evidence-based guideline for the management of high blood pressure in adults: report from the panel members appointed to the Eighth Joint National Committee (JNC 8). JAMA 2014;311:507–20. 10.1001/jama.2013.284427 24352797

[9] Pickering TG, Gerin W, Schwartz AR What is the white-coat effect and how should it be measured? Blood Press Monit 2002;7:293–300. 10.1097/00126097-200212000-00001 12488648

[10] Birkett NJ The effect of alternative criteria for hypertension on estimates of prevalence and control. J Hypertens 1997;15:237–44. 10.1097/00004872-199715030-00004 9468450

[11] McAlister FA, Straus SE Evidence based treatment of hypertension. Measurement of blood pressure: an evidence based review. BMJ 2001;322:908–11. 10.1136/bmj.322.7291.908 11302909 PMC1120072

[12] Carey IM, Nightingale CM, DeWilde S, et al Blood pressure recording bias during a period when the Quality and Outcomes Framework was introduced. J Hum Hypertens 2009;23:764–70. 10.1038/jhh.2009.18 19279657

[13] Doran T, Fullwood C, Gravelle H, et al Pay-for-performance programs in family practices in the United Kingdom. N Engl J Med 2006;355:375–84. 10.1056/NEJMsa055505 16870916

[14] Schiffrin EL, Calhoun DA, Flack JM Do We Need a New Definition of Hypertension After SPRINT? Am J Hypertens 2016;29:1127–9. 10.1093/ajh/hpw068 27334926

[15] Hippisley-Cox J, Coupland C, Vinogradova Y, et al Predicting cardiovascular risk in England and Wales: prospective derivation and validation of QRISK2. BMJ 2008;336:1475–82. 10.1136/bmj.39609.449676.25 18573856 PMC2440904

[16] Heneghan C, Perera R, Mant D, et al Hypertension guideline recommendations in general practice: awareness, agreement, adoption, and adherence. Br J Gen Pract 2007;57:948–52. 10.3399/096016407782604965 18252069 PMC2084133

[17] Meneton P, Ricordeau P, Weill A, et al Evaluation of the agreement between guidelines and initial antihypertensive drug treatment using a national health care reimbursement database. J Eval Clin Pract 2012;18:623–9. 10.1111/j.1365-2753.2011.01640.x 21276142

[18] Khatib R, Schwalm JD, Yusuf S, et al Patient and healthcare provider barriers to hypertension awareness, treatment and follow up: a systematic review and meta-analysis of qualitative and quantitative studies. PLoS One 2014;9:e84238 10.1371/journal.pone.0084238 24454721 PMC3893097

[19] Bartlett JE, Kotrlik JW, Higgins CC Organizational Research: Determining Appropriate Sample Size in Survey Research. Inf Technol Learn Perform J 2001;19:43–50.

[20] StataCorp. Stata Statistical Software: Release 14. College Station, Texas, USA: StataCorp LP, 2015.

[21] R Core Team. R: A language and environment for statistical computing. Vienna, Austria: R Foundation for Statistical Computing, 2016.

[22] Office for National Statistics. Internet users in the UK : 2016 [Internet]. 2016 https://www.ons.gov.uk/businessindustryandtrade/itandinternetindustry/bulletins/internetusers/2016

[23] Stamler J, Vaccaro O, Neaton JD, et al Diabetes, other risk factors, and 12-yr cardiovascular mortality for men screened in the Multiple Risk Factor Intervention Trial. Diabetes Care 1993;16:434–44. 10.2337/diacare.16.2.434 8432214

[24] Mercer SW, Guthrie B, Furler J, et al Multimorbidity and the inverse care law in primary care. BMJ 2012;344:e4152 10.1136/bmj.e4152 22718915

[25] Fletcher BR, Hinton L, Bray EP, et al Self-monitoring blood pressure in patients with hypertension: an internet-based survey of UK GPs. Br J Gen Pract 2016;66:e831–7. 10.3399/bjgp16X687037 27578811 PMC5072921

[26] Stevens SL Improving cardiovascular risk prediction through more accurate and alternative methods of blood pressure measurement. Oxford, UK: University of Oxford, 2017.

[27] A’Court C, Stevens R, Sanders S, et al Type and accuracy of sphygmomanometers in primary care: a cross-sectional observational study. Br J Gen Pract 2011;61:598–603. 10.3399/bjgp11X593884 22152749 PMC3162183

[28] Keenan K, Hayen A, Neal BC, et al Long term monitoring in patients receiving treatment to lower blood pressure: analysis of data from placebo controlled randomised controlled trial. BMJ 2009;338:b1492 10.1136/bmj.b1492 19406886 PMC2675695

[29] Verberk WJ, Kessels AG, de Leeuw PW Prevalence, causes, and consequences of masked hypertension: a meta-analysis. Am J Hypertens 2008;21:969–75. 10.1038/ajh.2008.221 18583985

[30] Kaczorowski J, Myers MG, Gelfer M, et al How do family physicians measure blood pressure in routine clinical practice? National survey of Canadian family physicians. Can Fam Physician 2017;63:e193–9. 28292817 PMC5349740

[31] Leung AA, Nerenberg K, Daskalopoulou SS, et al Hypertension Canada’s 2016 Canadian Hypertension Education Program Guidelines for Blood Pressure Measurement, Diagnosis, Assessment of Risk, Prevention, and Treatment of Hypertension. Can J Cardiol 2016;32:569–88. 10.1016/j.cjca.2016.02.066 27118291

[32] Mejzner N, Clark CE, Smith LF, et al Trends in the diagnosis and management of hypertension: repeated primary care survey in South West England. Br J Gen Pract 2017;67:e306–13. 10.3399/bjgp17X690461 28347984 PMC5409425

[33] National Institute for Health and Care Excellence. NICE CG28. Type 2 diabetes in adults: management. London: National Institute for Health and Care Excellence, 2015.

[34] Baral-Grant S, Haque MS, Nouwen A, et al Self-Monitoring of Blood Pressure in Hypertension: A UK Primary Care Survey. Int J Hypertens 2012;2012:1–4. 10.1155/2012/582068 PMC3195273 22013510

[35] Sheppard JP, Fletcher B, Gill P, et al Predictors of the Home-Clinic Blood Pressure Difference: A Systematic Review and Meta-Analysis. Am J Hypertens 2016;29:614–25. 10.1093/ajh/hpv157 26399981 PMC4829055

[36] Sheppard JP, Stevens R, Gill P, et al Predicting Out-of-Office Blood Pressure in the Clinic (PROOF-BP): Derivation and Validation of a Tool to Improve the Accuracy of Blood Pressure Measurement in Clinical Practice. Hypertension 2016;67:941–50. 10.1161/HYPERTENSIONAHA.115.07108 27001299 PMC4905620

[37] Clark CE, Taylor RS, Shore AC, et al Association of a difference in systolic blood pressure between arms with vascular disease and mortality: a systematic review and meta-analysis. Lancet 2012;379:905–14. 10.1016/S0140-6736(11)61710-8 22293369

[38] A’Court C, Sheppard J, Greenhalgh T Blood pressure measurement: a call to arms. Br J Gen Pract 2016;66:552–3. 10.3399/bjgp16X687637 27789487 PMC5072892

