# Supplementary material for: Current practice of usual clinic blood pressure measurement in people with and without diabetes: a survey and prospective ‘mystery shopper’ study in UK primary care
Source: BMJ Open. 2018 Apr 12;8(4):e020589. doi: 10.1136/bmjopen-2017-020589 (PMC5898319; doi:10.1136/bmjopen-2017-020589)
Supplement: Supplementary file 1 [file bmjopen-2017-020589supp001.pdf]

Supplementary material

Initial survey

NUFFIELD DEPARTMENT OF PRIMARY CARE HEALTH SCIENCES

Nuffield Department of Primary Care Health Sciences • University of Oxford • New Radcliffe House • Radcliffe Observatory Quarter • Woodstock Road • Oxford OX2 6GG • www.phc.ox.ac.uk

Sarah Stevens
+44 (0)1865 289449 • sarah.stevens@phc.ox.ac.uk

Survey of blood pressure measurement in primary care (Survey 1)

Q1 Have you got high blood pressure or have you ever been told by your GP that you have high blood pressure?
- ☐ Yes. Go to Q2
- ☐ No. Go to Q3

Q2 Do you take any medication for your high blood pressure?
- ☐ Yes
- ☐ No

Q3 When was your last appointment with a GP or practice nurse at your general practice surgery?
- ☐ In the last week
- ☐ In the last month
- ☐ In the last 6 months
- ☐ In the last year
- ☐ More than a year ago
- ☐ Can't remember

Q4 Who was your last appointment at the surgery with?
- ☐ GP
- ☐ Nurse
- ☐ Can't remember

Q5 Was your blood pressure measured during your last appointment?
- ☐ Yes, by a GP. Go to Q6
- ☐ Yes, by a nurse. Go to Q6
- ☐ Yes, by yourself in the waiting room. Go to Q6.
- ☐ No. Go to Q9
- ☐ Can't remember

Q6 How many times was your blood pressure measured during the appointment? Count one measurement for each time the cuff on your arm went up.
- ☐ Once
- ☐ Twice
- ☐ Three times
- ☐ Four or more times
- ☐ Can't remember

Q7 Did your GP/ nurse tell you your blood pressure reading or discuss it with you?
- ☐ Yes
- ☐ No
- ☐ Can't remember

Q8 Do you know your blood pressure reading from the appointment?
- ☐ Yes. Go to Q9
- ☐ No. Go to Q10
- ☐ Wasn't told. Go to Q10
- ☐ Can't remember. Go to Q10

Q9 What was your blood pressure reading during the appointment?
(Your blood pressure consists of two numbers: systolic pressure and diastolic pressure. Systolic pressure is always the larger of the two numbers. For example if your blood pressure was 130/80 mmHg, 130 is the systolic reading and 80 is the diastolic reading.)

Systolic ___________ Diastolic ___________

Q10 During your last appointment, were you asked to measure your blood pressure yourself away from the practice e.g. at home?

- ☐ Yes
- ☐ No
- ☐ Can't remember

Q11 Have you ever had your blood pressure measured on both arms at the same appointment?

- ☐ Yes
- ☐ No
- ☐ Can't remember

Q12 Are you

- ☐ Male
- ☐ Female

Q13 How old are you? ____________ years

Q14 Do you currently smoke?

- ☐ Yes
- ☐ No

Q15 Do you take any medication for high cholesterol e.g. statins?

- ☐ Yes
- ☐ No

Q16 Have you ever been told by your GP that you have had any of the following conditions or a chronic condition? (Tick all that apply)

- ☐ Diabetes (Type 1 or Type 2)
- ☐ Chronic kidney disease
- ☐ Stroke
- ☐ Heart attack
- ☐ Irregular heart beat (atrial fibrillation)
- ☐ Rheumatoid arthritis
- ☐ At high risk of having a heart attack or stroke
- ☐ Other. Please specify ___________________________
- ☐ None of the above

Q17 How did you hear about this survey?

- ☐ University of the Third Age
- ☐ Blood Pressure UK
- ☐ Other. Please specify ______________________

Q18 Please provide the first half of your postcode (e.g. ME19 4SH would be ME19. This will be used to analyse results by area only)

____

Q19 When do you expect your next appointment with your GP or nurse at your general practice surgery to be (confirmed or possible)?

- ☐ In the next week
- ☐ In the next two weeks
- ☐ In the next month
- ☐ In the next 3 months
- ☐ Don't know
- ☐ None scheduled

Q20 Would you be prepared to take part in a short (5 minute) follow-on survey after your next appointment?
(The follow-on survey will ask further, similar questions about whether and how your blood pressure was measured at the appointment. This will include how many times your blood pressure was measured and what the level of your blood pressure was.)

- ☐ Yes (Go to Q21)
- ☐ No (END)

Q21 In order to take part in the follow-on survey, the researcher will need to contact you by email and needs your consent to do this. Please complete the following participant declaration:

a) I have read the study information above, had the opportunity to ask questions and have received satisfactory answers (Q1 of 8)

- ☐ Yes

b) I understand that this project has been reviewed by, and received ethics clearance through, the University of Oxford Central University Research Ethics Committee (Q2 of 8)

- ☐ Yes

c) I understand that my participation is voluntary and I am free to withdraw myself and my data at any time, without giving any reason, and without any adverse consequences (Q3 of 8)

- ☐ Yes

d) I understand who will have access to personal data provided (Q4 of 8)

- ☐ Yes

e) I understand that personal data will be stored according to the Data Protection Act and will only be accessed by researchers from the Nuffield Department of Primary Care Health Sciences, University of Oxford (Q5 of 8)

- ☐ Yes

f) I understand that the research will be written up and published peer-reviewed journals, presented at research meetings and published online as part of a student thesis, deposited both in print and online in the University of Oxford archives (Q6 of 8)

- ☐ Yes

g) I understand how to raise concerns or make a complaint (Q7 of 8)

- ☐ Yes

h) I agree to take part in the study (Q8 of 8)

- ☐ Yes

i) Please enter your email address (This will only be used to contact you via email)

____

Thank you for taking the time to complete this survey.

Prospective follow-up survey

NUFFIELD DEPARTMENT OF **PRIMARY CARE** HEALTH SCIENCES

Nuffield Department of Primary Care Health Sciences • University of Oxford • New Radcliffe House • Radcliffe Observatory Quarter • Woodstock Road • Oxford OX2 6GG • www.phc.ox.ac.uk

Sarah Stevens
+44 (0)1865 289449 • sarah.stevens@phc.ox.ac.uk

Survey of blood pressure measurement in primary care (Survey 2)

Q1 Please enter your email address (the email address given in survey 1. This will be used to match your responses from both surveys.)

Email address _______________

Q3 When was your last appointment with a GP or practice nurse at your general practice surgery?

- ☐ In the last week
- ☐ In the last month
- ☐ In the last 6 months
- ☐ In the last year
- ☐ More than a year ago
- ☐ Can't remember

Q4 Who was your last appointment at the surgery with?

- ☐ GP
- ☐ Nurse
- ☐ Can't remember

Q5 Was your blood pressure measured during your last appointment?

- ☐ Yes, by a GP. Go to Q6
- ☐ Yes, by a nurse. Go to Q6
- ☐ Yes, by yourself in the waiting room. Go to Q6.
- ☐ No. Go to Q9
- ☐ Can't remember

Q6 How many times was your blood pressure measured during the appointment? Count one measurement for each time the cuff on your arm went up.

- ☐ Once
- ☐ Twice
- ☐ Three times
- ☐ Four or more times
- ☐ Can't remember

Q7 Did your GP/ nurse tell you your blood pressure level or discuss it with you?

- ☐ Yes
- ☐ No
- ☐ Can't remember

Q8 Do you know your blood pressure reading from the appointment?

- ☐ Yes. Go to Q9
- ☐ No. Go to Q10
- ☐ Wasn't told. Go to Q10
- ☐ Can't remember. Go to Q10

Q9 What was your blood pressure reading during the appointment?
(Your blood pressure consists of two numbers: systolic pressure and diastolic pressure. Systolic pressure is always the larger of the two numbers. For example if your blood pressure was 130/80 mmHg, 130 is the systolic reading and 80 is the diastolic reading.)

	Systolic	Diastolic
First measurement	___________	___________
Second measurement	___________	___________
Third measurement	___________	___________

Q10 During your last appointment, were you asked to measure your blood pressure yourself away from the practice e.g. at home?

- ☐ Yes
- ☐ No
- ☐ Can't remember

Thank you for taking the time to complete this survey.

Table S1: Characteristics of participants completing initial and follow-up surveys

		Completed 1st survey (N=743)	Completed prospective survey (N=334)
**Characteristic**		Mean (SD) / N (%)	Mean (SD) / N (%)
**Male**		377 (50.7)	172 (51.5)
**Age**		57.4 (13.28)	59.3 (12.14)
**Current smoker**		48 (6.5)	25 (7.5)
**Hypertensive**		413 (55.6)	200 (59.9)
	**Antihypertensive medication**	353 (85.5)	173 (86.5)
**Diabetes**		623 (83.9)	279 (83.5)
**Previous CVD**		62 (8.3)	31 (9.3)
**Chronic kidney disease**		25 (3.4)	12 (3.6)
**Rheumatoid arthritis**		23 (3.1)	13 (3.9)
**Told at high risk of CVD**		55 (7.4)	30 (9.0)
**Region**			
	**North East**	18 (2.4)	9 (2.7)
	**North West**	285 (38.4)	125 (37.4)
	**Yorkshire & The Humber**	55 (7.4)	20 (6.0)
	**East Midlands**	23 (3.1)	8 (2.4)
	**West Midlands**	38 (5.1)	16 (4.8)
	**East of England**	42 (5.7)	28 (8.4)
	**South West**	103 (13.9)	49 (14.7)
	**South East**	93 (12.5)	57 (17.1)
	**London**	50 (6.7)	15 (4.5)
	**Other**	8 (1.1)	2 (0.6)
	**Unknown**	28 (3.8)	5 (1.5)

Table S2: Participant blood pressure (BP) measurement at their last general practice appointment in those with or without diabetes (initial survey results)

	Participants with diabetes (N=623)	Participants without diabetes (N=120)
**Did not have their BP measured**	209 (33.6%)	45 (37.5%)
**Had their BP measured by a GP**	119 (19.1%)	37 (30.8%)
**Had their BP measured by a nurse**	286 (45.9%)	35 (29.2%)
**Measured their BP themselves in the waiting room**	9 (1.4%)	3 (2.5%)

Table S3: Number of blood pressure measurements taken in participants with and without diabetes who could recall how many BP readings were taken (initial survey results)

Number of blood pressure measurements	Number of participants with diabetes (N=408)	Number of participants without diabetes (N=72)
**1**	250 (61.3%)	36 (50.0%)
**2**	124 (30.4%)	20 (27.8%)
**3 or more**	34 (8.3)	16 (22.2%)

Table S4: Blood pressure values by reading number and total number of readings in 91 participants with diabetes who reported a value for each blood pressure reading in the prospective survey

	**Total number of readings**	**Mean (95% CI) [Range]**		
		**Reading number**		
		**1**	**2**	**3**
**Systolic**	**1 (n=66)**	130.0 (126.6 to 133.3) [110,176]	- -	- -
	**2 (n=19)**	139.4 (131.3 to 147.5) [100,173]	131.7 (122.0 to 141.4) [80, 173]	- -
	**3 (n=6)**	133.3 (127.0 to 139.7) [126, 140]	133.3 (127.0 to 139.6) [126, 140]	131.8 (125.3 to 138.4) [121, 139]
**Diastolic**	**1 (n=66)**	74.1 (72.2 to 76.1) [60, 95]	- -	- -
	**2 (n=19)**	84.2 (76.7 to 91.6) [65, 133]	79.6 (74.3 to 85.0) [62, 112]	- -
	**3 (n=6)**	83.3 (70.4 to 96.2) [66, 96]	82.5 (71.0 to 94.0) [69, 94]	80.2 (70.0 to 90.3) [68, 91]

Table S5: Blood pressure values by reading number and total number of readings in 20 participants without diabetes reporting a value for all blood pressure readings in the prospective survey.

	Total number of readings	Mean (95% CI) [Range]		
		Reading number		
		1	2	3
**Systolic**	**1 (n=12)**	135.8 (115.8 to 155.8) [110, 213]	- -	- -
	**2 (n=4)**	160.8 (124.9 to 196.6) [137,181]	158.5 (127.7 to 189.3) [137, 179]	- -
	**3 (n=4)**	154.5 (141.2 to 167.8) [147, 166]	152.3 (141.7 to 162.8) [147, 162]	144.3 (126.0 to 162.5) [128, 155]
**Diastolic**	**1 (n=12)**	75.3 (67.3 to 83.2) [60, 100]	- -	- -
	**2 (n=4)**	89.8 (75.6 to 103.9) [78, 98]	87.8 (70.5 to 105.0) [78, 99]	- -
	**3 (n=4)**	89.0 (63.1 to 114.9) [70, 108]	87.0 (64.5 to 109.5) [69, 101]	84.0 (61.9 to 106.1) [65, 97]

Table S6: Blood pressure measurement by diabetes status in a random sample of respondents from unique postcode districts

	**Participants with diabetes**<br>BP was measured in = 108/171 participants: 22 (20.4%) by a GP, 81 (75%) by a nurse and 5 (4.6%) by the patient		**Participants without diabetes**<br>BP was measured in = 24/41 participants: 15 (62.5%) by a GP, 7 (29.2%) by a nurse and 2 (8.3%) by the patient	
**Number of times BP measured**	**N (%)**	**Asked to measure BP at home (N, %)**	**N (%)**	**Asked to measure BP at home (N, %)**
**Once**	58 (53.7%)	6 (10.3%)	17 (70.8%)	3 (17.6%)
**Twice**	34 (31.5%)	7 (20.6%)	2 (8.3%)	2 (100.0%)
**Three or more**	16 (14.8%)	5 (31.3%)	5 (20.8%)	3 (60.0%)

Table S7: Blood pressure values by reading number and total number of readings in a random sample of respondents from unique postcode districts (stratified by diabetes status, in those reporting all BP readings)

	**Total number of readings**	**Mean (SD)**		
		**Reading number**		
		**1**	**2**	**3**
**Participants with diabetes reporting all BP readings (N=49; 31 (63.3%) had BP measured according to guidelines)**				
**Systolic**	**1 (n=35)**	128.0 (16.3)	-	-
	**2 (n=10)**	135.1 (11.8)	125.1 (19.3)	-
	**3 (n=4)**	132.5 (6.5)	132.0 (6.5)	130.8 (7.7)
**Diastolic**	**1 (n=35)**	75.8 (8.7)	-	-
	**2 (n=10)**	85.8 (19.1)	80.9 (13.6)	-
	**3 (n=4)**	78.5 (12.3)	79.0 (11.7)	77.8 (10.8)
**Participants without diabetes reporting all BP readings (N=15; 8 (53.3%) had BP measured according to guidelines)**				
**Systolic**	**1 (n=9)**	137.0 (36.8)	-	-
	**2 (n=2)**	163.5 (24.7)	159.0 (15.6)	-
	**3 (n=4)**	154.5 (8.3)	152.3 (6.6)	144.3 (11.5)
**Diastolic**	**1 (n=9)**	78.3 (12.8)	-	-
	**2 (n=2)**	93.0 (7.1)	89.0 (14.1)	-
	**3 (n=4)**	89.0 (6.3)	87.0 (14.1)	84.0 (13.9)

Table S8: Likelihood of having BP measured multiple times or being asked to monitor BP at home, according to patient and practitioner characteristics in patients with and without diabetes from in a random sample of responses from unique postcode districts

	**Likelihood of multiple BP measurements (n (%) in each group) (difference [95% confidence interval])**	**Likelihood of being asked to monitor BP at home (n (%) in each group) (difference [95% confidence interval])**
**In patients with diabetes**		
**If the patient is hypertensive vs. normotensive**	31/55 (56.4%) vs. 19/53 (35.9%) difference = 20.5% [2.1 to 38.9%]	14/88 (15.9%) vs. 10/83 (12.1%) difference = 3.9% [-6.5 to 14.2%]
**If the patient has treated hypertension vs. untreated hypertension**	27/48 (56.3%) vs. 4/7 (57.1%) difference = 0.9% [-38.4 to 40.1%]	13/75 (17.3%) vs. 1/13 (7.7%) difference = 9.6% [-7.2 to 26.5%]
**If BP was measured by a GP vs. a nurse**	12/22 (54.5%) vs. 36/81 (44.4%) difference = 10.1% [-13.4 to 33.6%]	7/22 (31.8%) vs. 9/81 (11.1%) difference = 20.7% [0.1 to 41.3%]
**In patients without diabetes**		
**If the patient is hypertensive vs. normotensive**	6/19 (31.6%) vs. 1/5 (20.0%) difference = 11.6% [-29.2 to 52.4%]	11/31 (35.5%) vs. 0/10 (0.0%) difference = 35.5% [18.6% to 52.3%]
**If the patient has treated hypertension vs. untreated hypertension**	5/15 (33.3%) vs. 1/4 (25.0%) difference = 8.3% [-40.3 to 57.0%]	7/23 (30.4%) vs. 4/8 (50.0%) difference = 19.6% [-19.9 to 59.0%]
**If BP was measured by a GP vs. a nurse**	5/15 (33.3%) vs. 2/7 (28.6%) difference = 4.8% [-36.3 to 45.9%]	6/15 (40.0%) vs. 2/7 (28.6%) difference = 11.4% [-30.2 to 53.1%]

Table S9: Blood pressure measurement by diabetes status, excluding possible duplicate submissions

	**Participants with diabetes**<br>BP was measured in = 172/263 participants: 36 (20.9%) by a GP, 130 (75.6%) by a nurse and 6 (3.5%) by the patient		**Participants without diabetes**<br>BP was measured in = 33/54 participants: 21 (63.6%) by a GP, 10 (30.3%) by a nurse and 2 (6.1%) by the patient	
**Number of times BP measured**	**N (%)**	**Asked to measure BP at home (N, %)**	**N (%)**	**Asked to measure BP at home (N, %)**
**Once**	100 (58.1%)	10 (10.0%)	22 (66.6%)	5 (22.7%)
**Twice**	50 (29.1%)	11 (22.0%)	5 (12.2%)	3 (60.0%)
**Three or more**	22 (12.8%)	7 (31.8%)	6 (18.2%)	3 (50.0%)

Table S10: Blood pressure values by reading number and total number of readings excluding possible duplicate submissions (stratified by diabetes status, in those reporting all BP readings)

	**Total number of readings**	**Mean (SD)**		
		**Reading number**		
		**1**	**2**	**3**
**Participants with diabetes reporting all BP readings (N=91; 58 (63.7%) had BP measured according to guidelines)**				
**Systolic**	**1 (n=66)**	130.0 (13.5)	-	-
	**2 (n=19)**	139.4 (16.8)	131.7 (20.1)	-
	**3 (n=6)**	133.3 (6.1)	133.3 (6.0)	131.8 (6.2)
**Diastolic**	**1 (n=66)**	74.1 (7.9)	-	-
	**2 (n=19)**	84.2 (15.4)	79.6 (11.1)	-
	**3 (n=6)**	83.3 (12.3)	82.5 (10.9)	80.2 (9.7)
**Participants without diabetes reporting all BP readings (N=20; 12 (60.0%) had BP measured according to guidelines)**				
**Systolic**	**1 (n=12)**	135.8 (31.5)	-	-
	**2 (n=4)**	160.8 (22.5)	158.5 (19.4)	-
	**3 (n=4)**	154.5 (8.3)	152.3 (6.7)	144.3 (11.5)
**Diastolic**	**1 (n=12)**	75.3 (12.5)	-	-
	**2 (n=4)**	89.8 (8.9)	87.8 (10.8)	-
	**3 (n=4)**	89.0 (16.3)	87.0 (14.1)	84.0 (13.9)

Table S11: Likelihood of having BP measured multiple times or being asked to monitor BP at home, according to patient and practitioner characteristics in patients with and without diabetes, excluding possible duplicate submissions

	**Likelihood of multiple BP measurements (n (%) in each group) (difference [95% confidence interval])**	**Likelihood of being asked to monitor BP at home (n (%) in each group) (difference [95% confidence interval])**
**In patients with diabetes**		
**If the patient is hypertensive vs. normotensive**	45/98 (45.9%) vs. 27/74 (36.5%)<br>difference = 9.4% [-5.3 to 24.2%]	24/153 (15.7%) vs. 11/110 (10.0%)<br>difference = 5.7% [-2.4 to 13.7%]
**If the patient has treated hypertension vs. untreated hypertension**	39/88 (44.3%) vs. 6/10 (60.0%)<br>difference = 15.7% [-16.4 to 47.8%]	22/135 (16.3%) vs. 2/18 (11.1%)<br>difference = 5.2% [-10.6 to 21.0%]
**If BP was measured by a GP vs. a nurse**	16/36 (44.4%) vs. 54/130 (41.5%)<br>difference = 2.9% [-15.4 to 21.2%]	11/36 (30.6%) vs. 15/130 (11.5%)<br>difference = 19.0% [3.0 to 35.0%]
**In patients without diabetes**		
**If the patient is hypertensive vs. normotensive**	9/27 (33.3%) vs. 2/6 (33.3%)<br>difference = 0.0% [-41.7 to 41.7%]	14/40 (35.0%) vs. 0/14 (0.0%)<br>difference = 35.0% [20.2 to 49.8%]
**If the patient has treated hypertension vs. untreated hypertension**	8/22 (36.4%) vs. 1/5 (20.0%)<br>difference = 16.4% [-24.1 to 56.8%]	9/31 (29.0%) vs. 5/9 (55.6%)<br>difference = 26.5% [-9.7 to 62.7%]
**If BP was measured by a GP vs. a nurse**	9/21 (42.9%) vs 2/10 (20.0%)<br>difference = 22.9% [-9.7 to 55.5%]	7/21 (33.3%) vs 4/10 (40.0%)<br>difference = 6.7% [-29.8 to 43.1%]
