# Supplementary material for: Current practice of usual clinic blood pressure measurement in people with and without diabetes: a survey and prospective ‘mystery shopper’ study in UK primary care
Source: BMJ Open. 2018 Apr 12;8(4):e020589. doi: 10.1136/bmjopen-2017-020589 (PMC5898319; doi:10.1136/bmjopen-2017-020589)
Supplement: Author's manuscript [file bmjopen-2017-020589.draft_revisions.pdf]

BMJ Open

BMJ Open is committed to open peer review. As part of this commitment we make the peer review history of every article we publish publicly available.

When an article is published we post the peer reviewers' comments and the authors' responses online. We also post the versions of the paper that were used during peer review. These are the versions that the peer review comments apply to.

The versions of the paper that follow are the versions that were submitted during the peer review process. They are not the versions of record or the final published versions. They should not be cited or distributed as the published version of this manuscript.

BMJ Open is an open access journal and the full, final, typeset and author-corrected version of record of the manuscript is available on our site with no access controls, subscription charges or pay-per-view fees (http://bmjopen.bmj.com).

If you have any questions on BMJ Open's open peer review process please email info.bmjopen@bmj.com

BMJ Open

Current practice of usual clinic blood pressure measurement in people with and without diabetes: a survey and prospective "mystery shopper" study in UK primary care

Journal:	*BMJ Open*
Manuscript ID	bmjopen-2017-020589
Article Type:	Research
Date Submitted by the Author:	10-Nov-2017
Complete List of Authors:	Stevens, Sarah; University of Oxford, Nuffield Department of Primary Care Health Sciences<br>McManus, Richard; University of Oxford, Dept of Primary Care Health Sciences<br>Stevens, Richard; University of Oxford, Nuffield Dept Primary Care Health Sciences
**Primary Subject Heading**:	Cardiovascular medicine
Secondary Subject Heading:	Evidence based practice, General practice / Family practice, Health services research, Epidemiology, Diabetes and endocrinology
Keywords:	PRIMARY CARE, Hypertension < CARDIOLOGY, General diabetes < DIABETES & ENDOCRINOLOGY, Protocols & guidelines < HEALTH SERVICES ADMINISTRATION & MANAGEMENT, Quality in health care < HEALTH SERVICES ADMINISTRATION & MANAGEMENT

SCHOLARONE™
Manuscripts

Title: Current practice of usual clinic blood pressure measurement in people with and without diabetes: a survey and prospective "mystery shopper" study in UK primary care

S L Stevens[1], R J McManus[1], R J Stevens[1]

1. Nuffield Department of Primary Care Health Sciences, University of Oxford, Radcliffe Observatory Quarter,Woodstock Road,Oxford,OX2 6GG

Corresponding author: Sarah Stevens, sarah.stevens@phc.ox.ac.uk

Manuscript word count: 2739

For peer review only

For peer review only - http://bmjopen.bmj.com/site/about/guidelines.xhtml

Abstract

Objectives: Hypertension trials and epidemiological studies use multiple clinic blood pressure (BP) measurements at each visit. Repeat measurement is also recommended in international guidance, however little is known about how BP is measured routinely. This is important for individual patient management and because routinely recorded readings form part of research databases. We aimed to determine the current practice of BP measurement during routine general practice appointments.

Design: (1) An online cross-sectional survey and (2) A prospective "mystery shopper" study where patients agreed to report how BP was measured during their next appointment.

Setting: Primary care

Participants: Patient charity/ involvement group members completing an online survey between July 2015 and January 2016. 334 participants completed the prospective study (51.5% male, mean age = 59.3 years) of which 279 (83.5%) had diabetes.

Primary outcome: Proportion of patients having BP measured according to guidelines.

Results: 217 participants with (183) and without diabetes (34) had their BP measured at their last appointment. BP was measured in line with UK guidance in 63.7% and 60.0% of participants with and without diabetes respectively. Initial pressures were significantly higher in those who had their BP measured more than once compared to only once (p=0.0.016/ 0.089 systolic and p<0.001/=0.022 diastolic, in patients with/without diabetes respectively).

Conclusions: Current practice of routine BP measurement in UK primary care is concordant with guidelines in the majority of cases. Guidelines may be less robust than primary study protocols and miss masked hypertension. Apparent relationships between BP and outcomes may be attenuated in studies using routine electronic healthcare databases which do not account for this.

Strengths and limitations of this study

- This survey has a novel "mystery-shopper" design, minimising biases that may be introduced by self-reported practitioner behaviour.
- We have examined how adherence to guidelines varies according to patient characteristics, whereas previous studies have taken a healthcare professional view.
- The use of an online survey may have resulted in an under-representation of some groups, such as the very elderly.
- Larger studies are required to confirm our findings with respect to second and third blood pressure readings.

For peer review only - http://bmjopen.bmj.com/site/about/guidelines.xhtml

Introduction

Measurement of blood pressure (BP) is carried out in general practice by healthcare professionals on a daily basis. Such measurement is important for the diagnosis and management of hypertension, a major risk factor for cardiovascular disease (CVD) in both the general population,(1) and even more so in those with diabetes.(2) Hypertension trials and major epidemiological studies typically measure clinic BP using strict protocols on three or more times per visit. For example the SPRINT(3) and ACCORD(4) trials used the mean of three readings taken automatically to guide treatment. Repeated measurement protocols are also recommended in the UK,(5) European,(6) and North American(7) hypertension guidelines. For example, current UK guidance states that BP should be re-measured if it is initially high, or if two measurements differ substantially, with out-of-office monitoring recommended in those with sustained high BP in clinic.(5)This reflects concerns that in many patients, clinic BP readings, particularly initial readings, may be systematically higher than BP during usual daily activities.(8)

Many factors can affect the accuracy of BP measurement and the number of measurements used can influence estimates of BP control.(9,10) Measurement practices may also vary depending on the focus of the consultation or patient characteristics and recorded blood pressures may also be influenced by incentive schemes such as the UK Quality and Outcomes Framework.(11,12) Potential differences between primary study protocols and clinical practice have implications for the generalisability and implementation of research findings. For example, SPRINT found that treatment to a systolic BP target of 120 mmHg resulted in fewer cardiovascular events compared to a target of 140 mmHg in low risk patients.(4) However others have argued that mean automatically measured BP of 120 mmHg may correspond to a routine measurement of 135-140 mmHg(15) and ACCORD (conducted in patients with diabetes) failed to show an effect of intensive treatment.(5)

Furthermore, increasing numbers of observational studies in electronic healthcare databases rely on routinely collected BP measurements. In particular, the recommended cardiovascular risk calculator in the UK, QRISK2,(14) was derived using such data. It is important to understand how blood pressures recorded in these databases were obtained, in order to reliably compare observational database and primary study results.

However, little is known about how BP is measured in routine practice. A 2006 survey of UK general practitioners' (GPs') adherence to hypertension guideline recommendations relied on self-reported data and did not ask about the use of repeat measurements.(15) Other European studies have focussed on whether implementation of lifestyle or treatment changes adheres to guidelines,(16) or reasons for non-adherence.(17) These studies assume that an accurate BP reading is obtained initially and ignore the specifics of BP measurement. We therefore sought to determine the current practice of BP measurement during routine appointments in UK primary care.

Patients and methods

We conducted an online survey of patients, followed by a prospective survey of primary care consultations.

Online survey

For peer review only - http://bmjopen.bmj.com/site/about/guidelines.xhtml

An online survey was advertised through charities and patient involvement groups (“University of the Third Age”, “Blood Pressure UK”, “Citizen Scientist”, “Patients Active in Research”, “Call for Participants” and “Research for the Future (Help BEAT Diabetes)”) between 23rd July 2015 and 24th January 2016. Respondents anonymously reported basic demographic and health information, if and how their BP was measured at their last appointment and (recall permitting) their last BP reading (Supplement).

Prospective study

Participants completing the online survey were invited to take part in a prospective study. They were told the study would ask similar questions to those already asked about their BP after their next primary care appointment. Those wishing to take part gave explicit consent, provided an email address and were asked when they expected their next appointment to be. After the anticipated time of this appointment, a link to an online questionnaire was emailed to participants. This asked whether BP was measured at the appointment; and if so, how many times, and (recall permitting) for up to three systolic and diastolic BP values (Supplement). The questionnaire was open from 23rd July 2015 to 16th June 2016.Two patient representatives helped design the study materials and three were asked to pilot the survey websites to test functionality. The study was approved by the Medical Sciences Interdivisional Research Ethics Committee, University of Oxford (reference: MS-IDREC-C1-2015-095).

Statistical Analysis

The prospective study was powered to estimate the proportion of people having their BP measured in line with guidelines at the 95% confidence level with an accuracy of +/-5%. Assuming a proportion of 10%, 139 respondents who had had their BP measured was required.(18)

Demographic and clinical history data were summarised using means and standard deviations or proportions. Mean BP was summarized with 95% confidence intervals and ranges. Respondents were classified as hypertensive if they answered yes to the question “Have you got high blood pressure or have you ever been told by your GP that you have high blood pressure?”. Responses were assessed against NICE guidance and BP was deemed to have been measured according to guidelines if BP was measured; a) once and the reading was below 140/90 mmHg, b) twice if the initial reading was above 140/90 mmHg and the first two readings differed by less than 5 mmHg systolic, or c) three times if the first reading was above 140/90 mmHg and the first two readings differed by more than 5 mmHg systolic.(5) Proportions were compared using two-sided tests of proportions, under the assumption of large samples, at the 5% level. Due to an unexpectedly large proportion of participants with diabetes, a decision to stratify all prospective study analyses by patient diabetes status was made after data collection.

Since behaviour amongst professionals from the same practice may be similar, sensitivity analyses were carried out by randomly selecting one observation from each postcode district (assuming respondents from different districts are registered to distinct practices). We also conducted sensitivity analyses excluding prospective responses that were suspected of being duplicate submissions of the same initial survey data. Analysis was conducted using Stata 14,(19) and R 3.3.1.(20)

Results

For peer review only - http://bmjopen.bmj.com/site/about/guidelines.xhtml

In total 2176 unique users visited the survey site, of whom 756 completed the initial online survey, with complete data available in 743 individuals (623 with diabetes, 83.9%). Consent for the prospective study was given by 593 participants and was completed by 334 participants (279 with diabetes, 83.5%) (Fig. 1). The characteristics of those completing the initial and prospective surveys were broadly similar (Table S1, Supplement).

Initial survey

Of the 743 people completing the first survey, 489 (65.8%) reported having had their BP measured at their last appointment: 156 (31.9% of 489) by a GP, 321 (65.6%) by a nurse and 12 (2.5%) in the waiting room. Most respondents (480/489, 98.2%) could recall how many BP readings were taken: 286 (59.6% of 480) one, 144 (30.0%) two and 50 (10.4%) three or more readings. Results stratified by diabetes status are given in the supplement (Tables S2 and S3). Only 88 patients (11.8%) recalled ever having their BP measured in both arms at any one previous appointment. Compared to normotensives (20/330, (6.7%)), respondents with a previous diagnosis of hypertension (68/413, (16.5%)) were more likely to report having had their BP measured in both arms at any appointment previously.

Prospective study

Baseline characteristics for those with and without diabetes completing the prospective study after a further GP appointment are given in Table 1. Of the 279 participants with diabetes completing the follow-up questionnaire, 183 (65.6%) had their BP measured at the appointment: 38 (20.8%) by a GP, 139 (76.0%) by a nurse and 6 (3.3%) by themselves in the waiting room. Of the 55 participants without diabetes, 34 (61.8%), had their BP measured: 21 (61.8%) by a GP, 11 (32.4%) by a nurse and 2 (5.9%) by themselves in the waiting room.

Participants with diabetes

Of the 183 participants with diabetes who had their BP measured, 91 (49.7%) could recall a value for all of the BP readings given. Fifty-eight respondents (63.7%, 95% CI [53.0 to 73.6%]) had their BP measured according to guidelines. Mean BP values by reading number are presented graphically in Fig. 2A (systolic) and Fig. 2B (diastolic, see Table S4 for raw data). Initial systolic and diastolic blood pressures were lower in participants who had their BP measured only once than in those who had it measured two or more times (mean systolic difference = 8.0 mm Hg, 95% CI [1.2 to 14.5 mm Hg], p=0.016 and mean diastolic difference = 9.9 mm Hg, 95% CI [5.1 to 14.6 mm Hg], p<0.001).

The proportion of participants with diabetes who had their BP measured multiple times was similar regardless of hypertensive or treatment status, or measurement personnel (Table 2, top left). However they were more likely to be asked to monitor their BP at home when BP was measured by a GP compared to a nurse (Table 2, top right). Those who had their BP measured once, twice and three or more times, were asked to monitor their BP at home in 10/109 (9.2%, 95% CI [3.8 to 14.6%]), 11/51 (21.6%, 95% CI [10.3% to 32.9%]) and 7/23 (30.4%, 95% CI [11.6 to 49.2%]) cases respectively.

Participants without diabetes

Of the 34 participants without diabetes who had their BP measured, 20 (58.8%) could recall a value for all of the BP readings given. Twelve respondents (60.0%, 95% CI [36.1% to 80.9%]) had their BP

For peer review only - http://bmjopen.bmj.com/site/about/guidelines.xhtml

measured according to guidelines. Mean BP values by reading number are presented graphically in Fig. 3A (systolic) and Fig. 3B (diastolic, see Table S5 for raw data). Patterns of repeat BP measurement were similar to those observed in participants with diabetes, although numbers in this group were smaller. Initial systolic blood pressures were non-significantly lower in participants who had their BP measured only once than in those who had it measured two or more times (mean systolic difference = 21.8 mm Hg, 95% CI [-3.7 to 47.3 mm Hg], p=0.089). However, a significant difference was observed for diastolic pressure (mean diastolic difference = 14.1 mm Hg, 95% CI [2.3 to 26.0 mm Hg], p=0.022).

The proportion of participants without diabetes who had their BP measured multiple times was similar regardless of hypertensive or treatment status, or measurement personnel (Table 2, bottom left). However, those with hypertension were more likely to be asked to monitor their BP at home compared to normotensives (Table 2, bottom right).Those who had their BP measured once, twice and three or more times, were asked to monitor their BP at home in 5/23 (21.7%, 95% CI [4.9 to 38.6%]), 3/5 (60.0%, 95% CI [17.1% to 100.0%]) and 3/6 (50.0%, 95% CI [10.0 to 90.0%]) cases respectively.

Sensitivity analyses

Results were similar after randomly sampling responses from unique postcode districts (Tables S6 to S8) or when excluding prospective responses suspected of being duplicate submissions of the initial survey data (Tables S9 to S11).

Discussion

Summary

This study has shown that a second BP measurement at clinic visit is more likely to be taken if the initial BP measurement is high. This is consistent with UK guidelines. However, there is no clear evidence that the decision to take a third measurement follows guidelines. The recommendation that a third measurement be taken only when the first two are discrepant (first measurement above threshold but second below threshold for diagnosis of hypertension) was not obviously reflected in our data, although confidence intervals are wide. Although the majority of this evidence relates to people with diabetes, similar BP measurement practices were observed in those without diabetes.

Strengths and limitations

The patient centred nature of this study has allowed us to see into the consulting room for the first time and to determine how BP is measured in “real life”, in those with and without diabetes. Previous studies have taken a healthcare professional view.(15)

Our online survey was limited by the use of convenience mechanisms for recruitment, and like many internet surveys with no known denominator, these results should be interpreted with caution. The use of an online system itself may have resulted in an under-representation of some groups, such as the very elderly.(21) For the prospective study, we were able to obtain “mystery shopper” type data on more than two hundred GP and nurse appointments without potentially influencing the appointment through direct observation by a researcher. To our knowledge, these data are unique. The lower numbers of respondents without diabetes could limit generalisability if health care

For peer review only - http://bmjopen.bmj.com/site/about/guidelines.xhtml

professionals follow protocols less carefully in patients without additional cardiovascular risk factors.(22)

Furthermore, self-reported BP readings may have been subject to rounding error, digit preference or recall error. This introduces uncertainty into some analyses concerning blood pressure values, but the number of measurements taken, is likely to be recalled with greater accuracy, especially in the prospective study.

Fewer than anticipated participants provided all BP readings and therefore we could only estimate the proportion of people with diabetes having their BP measured according to guidelines with an error of +/- 10% (compared to an original target of +/-5%). However we have demonstrated important differences (for example in first systolic BP readings) despite this. Although we have demonstrated that BP is measured in line with guidance in the majority of cases, this was driven by a large number of participants with low BP who had their BP measured only once. Larger studies would be required to confirm our findings, particularly with respect to second and third readings and in those without diabetes.

Comparison with existing literature

A previous review(17) of barriers to hypertension awareness and treatment found that professionals were concerned about the accuracy of individual clinic BP readings. Our results support the idea that professionals treat single readings with caution, particularly those above the diagnostic threshold. This behaviour is concordant with guidelines but as guidance does not reflect measurement in studies of blood pressure monitoring, GPs may be better advised to use multiple readings more widely.(23)

The finding that patients are more likely to be monitored at home if they have high clinic pressures or hypertension is consistent with results from a recent practitioner survey in Canada, (24) where guidance is similar.(25) A recent survey of general practices in the South West of England found that only 1 in 10 GP practices were not following current guidelines for the use of home and ambulatory BP monitoring in the diagnosis of hypertension,(26) which is also consistent with our results in that guidance appears to be followed in most cases.

Implications for research and practice

Our findings indicate that routine BP measurement does not reflect the strict measurement protocols in primary research studies. This has implications for patient care if results from primary research studies cannot be appropriately translated into guidance for routine care (e.g. in the form of adjusted treatment targets). Users of electronic healthcare databases should also be aware of the potential for recording biases(11) which may dilute the observed effect of BP on outcomes and may extend to other biological factors subject to measurement error.

The current practice of BP measurement will, reassuringly, detect white coat hypertension but may not identify those with masked effects (where BP is higher outside of the clinic). This could potentially result in missed diagnoses and sub-optimal treatment. One solution which would not increase workload is use of the PROOF-BP tool which was developed by two of the authors with colleagues.(27) This combines factors associated with home-clinic BP differences with BP readings to identify which patients may exhibit masked or white coat effects and would benefit most from out-of-office monitoring. It accurately identifies hypertension in 93% of cases and is more accurate than

For peer review only - http://bmjopen.bmj.com/site/about/guidelines.xhtml

For peer review only

current diagnostic guidelines.(28) Implementation of this tool could improve detection of masked effects and avoid unnecessary out-of-office monitoring.

Less than 1 in 5 participants with hypertension reported having their BP measured in both arms at a single appointment previously. Large differences between arms are associated with vascular disease and mortality.(29) These results suggest little change since 13% of GPs said they adhered to this recommendation a decade ago.(15) Other recent estimates suggest that around half of practices measure BP in both arms as part of the diagnostic procedure,(26) which, although more optimistic, further demonstrates room for improvement. Barriers to such improvement may include practitioner discordance with guidance (previously only 30% agreed with the recommendation), or a lack of a suitable devices.(30)

The results of this study provide a preliminary insight into how BP is measured routinely and indicate that BP is measured in line with guidelines but not with strict study protocols. The impact of these differences on patient care requires further investigation.

Funding

This project is funded by the National Institute for Health Research School for Primary Care Research (NIHR SPCR). The views expressed are those of the author(s) and not necessarily those of the NHS, the NIHR or the Department of Health.

Conflicts of interest

RM has received BP monitoring equipment for research purposes from Lloyds Pharmacies and Omron.

Contributions

All authors conceived and designed the study. SS was responsible for the management of the study and carried out the statistical analysis. SS drafted the paper which RS and RM then contributed to. We thank computer programmers David Judge and Luis Castello (Nuffield Department of Primary Care Health Sciences, University of Oxford) who developed and managed the survey sites. We also thank patient representatives Derek Shaw, Valerie Keston-Hole and others for their help developing the study materials.

Data sharing

No additional data are available.

For peer review only - http://bmjopen.bmj.com/site/about/guidelines.xhtml

References

1. Lewington S, Clarke R, Qizilbash N, Peto R, Collins R. Age-specific relevance of usual blood pressure to vascular mortality: A meta-analysis of individual data for one million adults in 61 prospective studies. Lancet. 2002;360(9349):1903–13.

2. Adler AI, Stratton IM, Neil HAW, Yudkin JS, Matthews DR, Cull CA, et al. Association of systolic blood pressure with macrovascular and microvascular complications of type 2 diabetes (UKPDS 36): prospective observational study. BMJ. 2000;321(7258).

3. The SPRINT Research Group. A Randomized Trial of Intensive versus Standard Blood-Pressure Control. N Engl J Med. 2015 Nov 26;373(22):2103–16.

4. The ACCORD Study Group. Effects of Intensive Blood-Pressure Control in Type 2 Diabetes Mellitus. N Engl J Med. Massachusetts Medical Society; 2010 Apr 29;362(17):1575–85.

5. National Institute for Health and Care Excellence. Hypertension: Clinical management of primary hypertension in adults (NICE CG127). National Institute for Health and Care Excellence; 2011.

6. Mancia G, Fagard R, Narkiewicz K, Redon J, Zanchetti A, Böhm M, et al. 2013 ESH/ESC guidelines for the management of arterial hypertension: the Task Force for the Management of Arterial Hypertension of the European Society of Hypertension (ESH) and of the European Society of Cardiology (ESC). Eur Heart J. 2013 Jul 14;34(28):2159–219.

7. James PA, Oparil S, Carter BL, Cushman WC, Dennison-Himmelfarb C, Handler J, et al. Evidence-Based Guideline for the Management of High Blood Pressure in Adults. Jama. 2013;1097(5):1–14.

8. Pickering TG, Gerin W, Schwartz AR. What is the white-coat effect and how should it be measured? Blood Press Monit. 2002;7(6):329–33.

9. Birkett NJ. The effect of alternative criteria for hypertension on estimates of prevalence and control. J Hypertens. 1997;15:237–44.

10. McAlister FA, Straus SE. Evidence based treatment of hypertension: Measurement of blood presssure: an evidence based review. BMJ. 2001;322(7291):908–11.

11. Carey IM, Nightingale CM, DeWilde S, Harris T, Whincup PH, Cook DG. Blood pressure recording bias during a period when the Quality and Outcomes Framework was introduced. J Hum Hypertens. 2009 Nov 12;23(11):764–70.

12. Doran T, Fullwood C, Gravelle H, Reeves D, Kontopantelis E, Hiroeh U, et al. Pay-for-performance programs in family practices in the United Kingdom. N Engl J Med. 2006 Jul 27;355(4):375–84.

13. Schiffrin EL, Calhoun DA, Flack JM. Do We Need a New Definition of Hypertension After SPRINT? Am J Hypertens. 2016 Oct;29(10):1127–9.

14. Hippisley-Cox J, Coupland C, Vinogradova Y, Robson J, Minhas R, Sheikh A, et al. Predicting cardiovascular risk in England and Wales: prospective derivation and validation of QRISK2. BMJ. 2008;336(7659):1475–82.

15. Heneghan C, Perera R, Mant D, Glasziou P. Hypertension guideline recommendations in general practice: awareness, agreement, adoption, and adherence. Br J Gen Pract. 2007 Dec;57(545):948–52.

For peer review only

For peer review only - http://bmjopen.bmj.com/site/about/guidelines.xhtml

16. Meneton P, Ricordeau P, Weill A, Tuppin P, Samson S, Allemand H, et al. Evaluation of the agreement between guidelines and initial antihypertensive drug treatment using a national health care reimbursement database. J Eval Clin Pract. 2012 Jun;18(3):623–9.

17. Khatib R, Schwalm J-D, Yusuf S, Haynes RB, McKee M, Khan M, et al. Patient and healthcare provider barriers to hypertension awareness, treatment and follow up: a systematic review and meta-analysis of qualitative and quantitative studies. PLoS One. 2014 Jan 15;9(1):e84238.

18. Bartlett JE, Kotrlik JW, Higgins CC. Organizational Research: Determining Appropriate Sample Size in Survey Research . Inf Technol Learn Perform J. 2001;19(1).

19. StataCorp. Stata Statistical Software: Release 14. College Station, Texas, USA: StataCorp LP; 2015.

20. R Core Team. R: A language and environment for statistical computing. R Foundation for Statistical Computing, Vienna, Austria; 2016.

21. Office for National Statistics. Internet users in the UK : 2016. 2016.

22. Stamler J, Vaccaro O, Neaton JD, Wentworth D. Diabetes, Other Risk Factors, and 12-Yr Cardiovascular Mortality for Men Screened in clusively of people with the Multiple Risk Factor Intervention Trial. Diabetes Care. 1993;16(2):434–44.

23. Keenan K, Hayen A, Neal BC, Irwig L. Long term monitoring in patients receiving treatment to lower blood pressure: analysis of data from placebo controlled randomised controlled trial. BMJ. 2009;338.

24. Kaczorowski J, Myers MG, Gelfer M, Dawes M, Mang EJ, Berg A, et al. How do family physicians measure blood pressure in routine clinical practice? Can Fam Physician. 2017;63(3).

25. Leung AA, Nerenberg K, Daskalopoulou SS, McBrien K, Zarnke KB, Dasgupta K, et al. Hypertension Canada's 2016 Canadian Hypertension Education Program Guidelines for Blood Pressure Measurement, Diagnosis, Assessment of Risk, Prevention, and Treatment of Hypertension. Can J Cardiol. 2016;32(5):569–88.

26. Mejzner N, Clark CE, Smith LF, Campbell JL. Trends in the diagnosis and management of hypertension: repeated primary care survey in South West England. Br J Gen Pract. 2017;67(658).

27. Sheppard JP, Fletcher B, Gill P, Martin U, Roberts N, McManus RJ. Predictors of the Home-Clinic Blood Pressure Difference: A Systematic Review and Meta-Analysis. Am J Hypertens. 2016 May;29(5):614–25.

28. Sheppard JP, Stevens R, Gill P, Martin U, Godwin M, Hanley J, et al. Predicting Out-of-Office Blood Pressure in the Clinic (PROOF-BP). Hypertension. 2016 May;67(5):941–50.

29. Clark CE, Taylor RS, Shore AC, Ukoumunne OC, Campbell JL. Association of a difference in systolic blood pressure between arms with vascular disease and mortality: a systematic review and meta-analysis. Lancet. 2012 Mar 10;379(9819):905–14.

30. A'Court C, Sheppard J, Greenhalgh T. Blood pressure measurement: a call to arms. Br J Gen Pract. 2016;66(652).

For peer review only - http://bmjopen.bmj.com/site/about/guidelines.xhtml

For peer review only

For peer review only - http://bmjopen.bmj.com/site/about/guidelines.xhtml

Table 1: Characteristics of participants completing the prospective survey with and without diabetes

		Participants with diabetes (N=279)	Participants without diabetes (N=55)
**Characteristic**		Mean (SD) / N (%)	Mean (SD) / N (%)
**Male**		157 (56.3)	15 (27.3)
**Age**		59.0 (12.1)	60.3 (12.7)
**Current smoker**		21 (7.5)	4 (7.3)
**Hypertensive**		159 (57.0)	41 (74.6)
	**Antihypertensive medication**	141 (88.7)	32 (78.0)
**Previous CVD**		29 (10.4)	2 (3.6)
**Chronic kidney disease**		11 (3.9)	1 (1.8)
**Rheumatoid arthritis**		12 (4.3)	1 (1.8)
**Told at high risk of CVD**		26 (9.3)	4 (7.3)
**Region**			
	**North East**	9 (3.2)	0 (0.0)
	**North West**	111 (39.8)	14 (25.5)
	**Yorkshire & The Humber**	19 (6.8)	1 (1.8)
	**East Midlands**	6 (2.2)	2 (3.6)
	**West Midlands**	13 (4.7)	3 (5.5)
	**East of England**	22 (7.9)	6 (10.9)
	**South West**	40 (14.3)	9 (16.4)
	**South East**	42 (15.1)	15 (27.3)
	**London**	13 (4.7)	2 (3.6)
	**Other**	0 (0.0)	2 (3.6)
	**Unknown**	4 (1.4)	1 (1.8)

For peer review only

For peer review only - http://bmjopen.bmj.com/site/about/guidelines.xhtml

Table 2: Likelihood of having BP measured multiple times or being asked to monitor BP at home, according to patient and practitioner characteristics (stratified by diabetes status)

	Likelihood of multiple BP measurements<br>(n (%) in each group)<br>(difference [95% confidence interval])	Likelihood of being asked to monitor BP at home<br>(n (%) in each group)<br>(difference [95% confidence interval])
In participants with diabetes		
If the participant was hypertensive vs. normotensive	46/103 (44.7%) vs. 28/80 (35.0%)<br>difference = 9.7% [-4.5 to 23.9%])	24/159 (15.1%) vs. 13/120 (10.8%)<br>difference=4.3% [-3.6 to 12.1%]
If the participant had treated hypertension vs. untreated hypertension	40/93 (43.0%) vs. 6/10 (60.0%)<br>difference = -17.0% [-49.0 to 15.0%]	22/141 (15.6%) vs. 2/18 (11.1%)<br>difference = 4.5% [-11.2 to 20.2%]
If BP was measured by a GP vs. a nurse	16/38 (42.1%) vs. 56/139 (40.3%)<br>difference = 1.8% [-15.9 to 19.5%]	11/38 (28.9%) vs. 15/139 (10.8%)<br>difference = 18.2% [2.8 to 33.5%]
In participants without diabetes		
If the participant was hypertensive vs. normotensive	2/6 (33.3%) vs. 9/28 (32.1%)<br>difference = 1.2% [-40.3 to 42.7%]	14/41 (34.1%) vs. 0/14 (0.0%)<br>difference = 34.1% [19.6 to 48.7%]
If the participant had treated hypertension vs. untreated hypertension	8/23 (34.8%) vs. 1/5 (20.0%)<br>difference = 14.8% [-25.3 to 54.9%]	9/32 (28.1%) vs. 5/9 (55.6%)<br>difference = -27.4% [-63.4% to 8.6%]
If BP was measured by a GP vs. a nurse	9/21 (42.9%) vs. 2/11 (18.2%)<br>difference = 24.7% [-6.4 to 55.8%]	7/21 (33.3%) vs. 4/11 (36.4%)<br>difference = -3.0% [-37.9 to 31.8%]

For peer review only - http://bmjopen.bmj.com/site/about/guidelines.xhtml

Fig. 1: Study flowchart

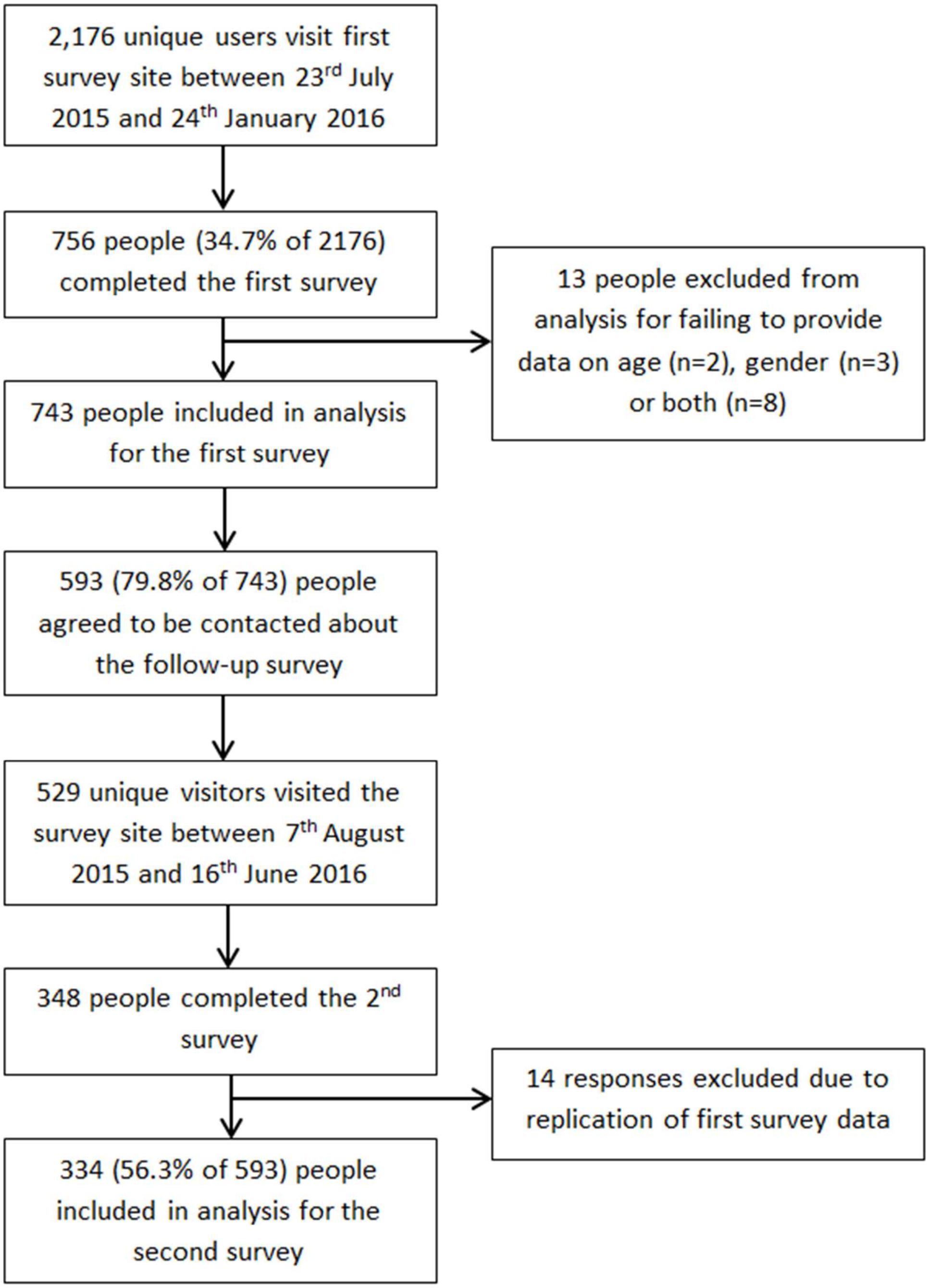

Fig.2: Mean blood pressure and 95% confidence intervals by reading number in 91 participants with diabetes who reported a value for each blood pressure reading in the prospective survey

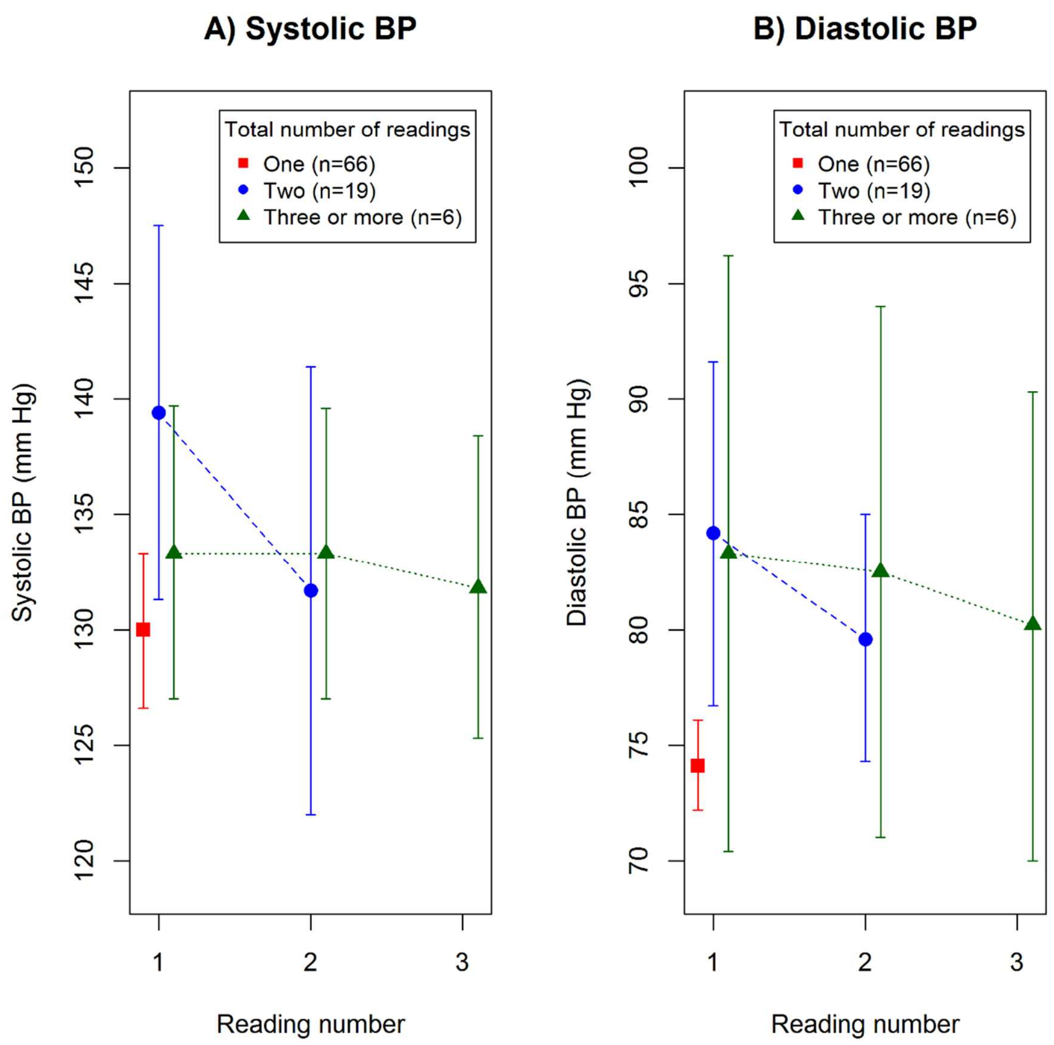

For peer review only - http://bmjopen.bmj.com/site/about/guidelines.xhtml

Fig. 3: Mean blood pressure and 95% confidence intervals by reading number in 20 participants without diabetes who reported a value for each blood pressure reading in the prospective survey

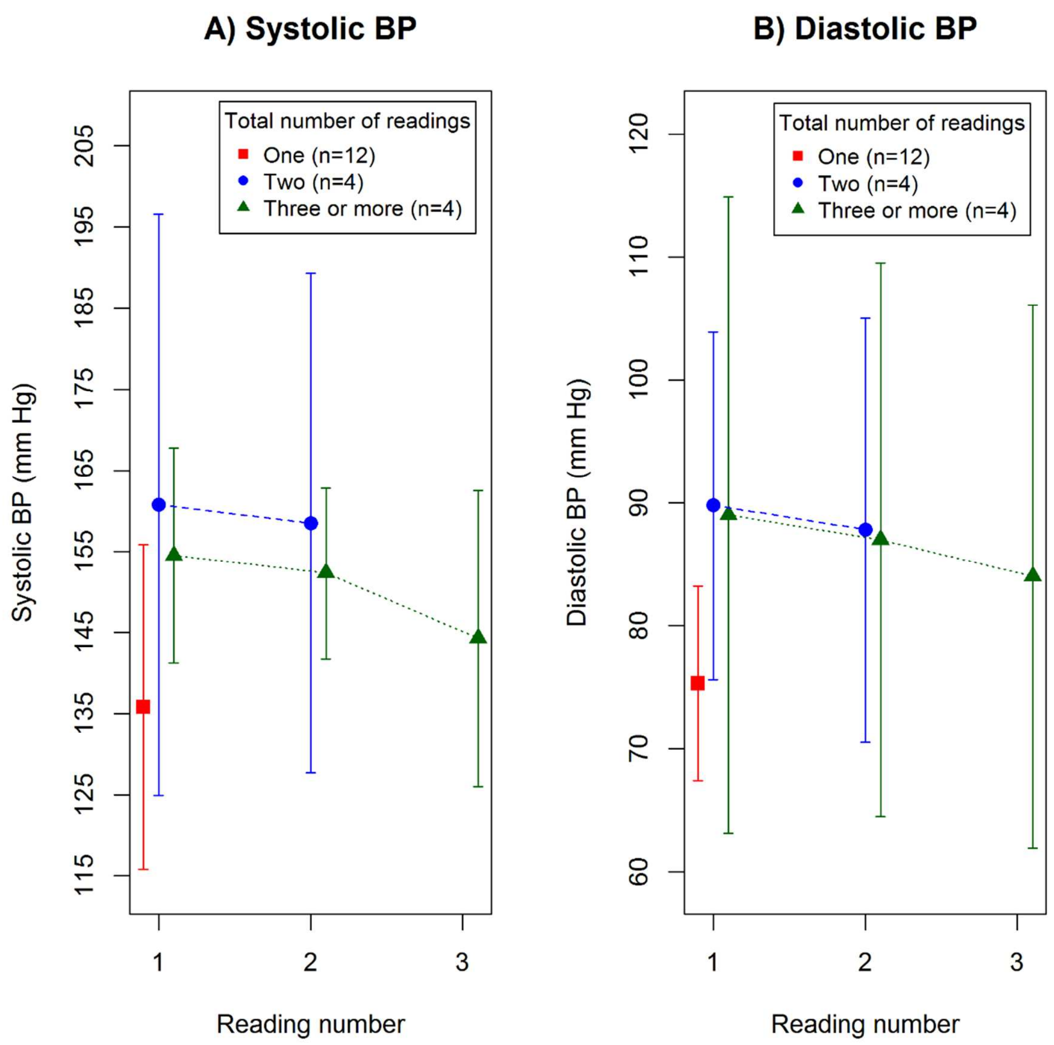

For peer review only - http://bmjopen.bmj.com/site/about/guidelines.xhtml

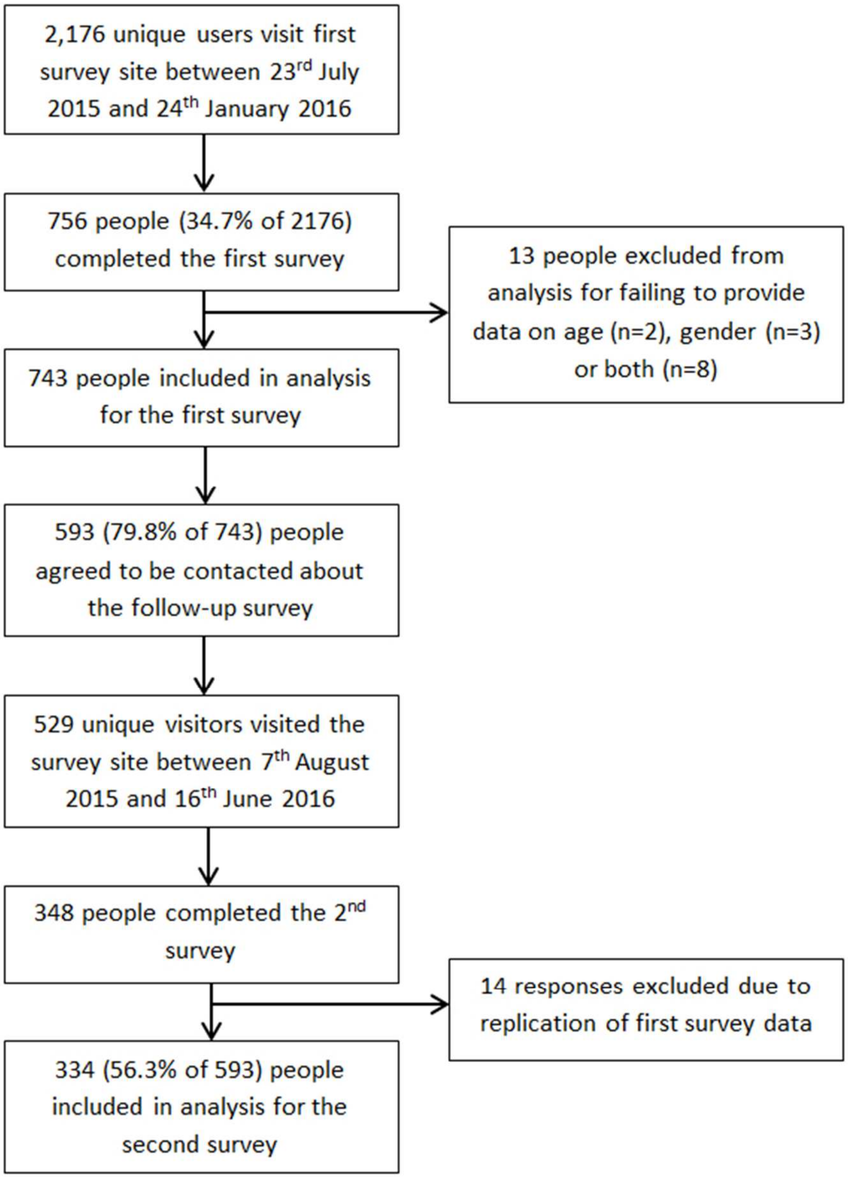

Fig. 1: Study flowchart

175x212mm (96 x 96 DPI)

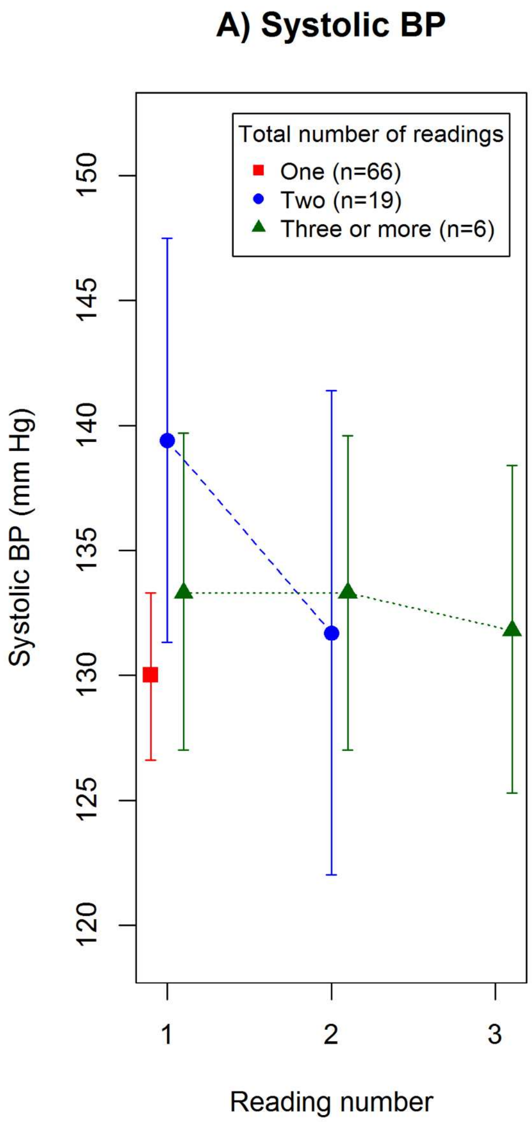

Fig.2: Mean blood pressure and 95% confidence intervals by reading number in 91 participants with diabetes who reported a value for each blood pressure reading in the prospective survey

179x360mm (300 x 300 DPI)

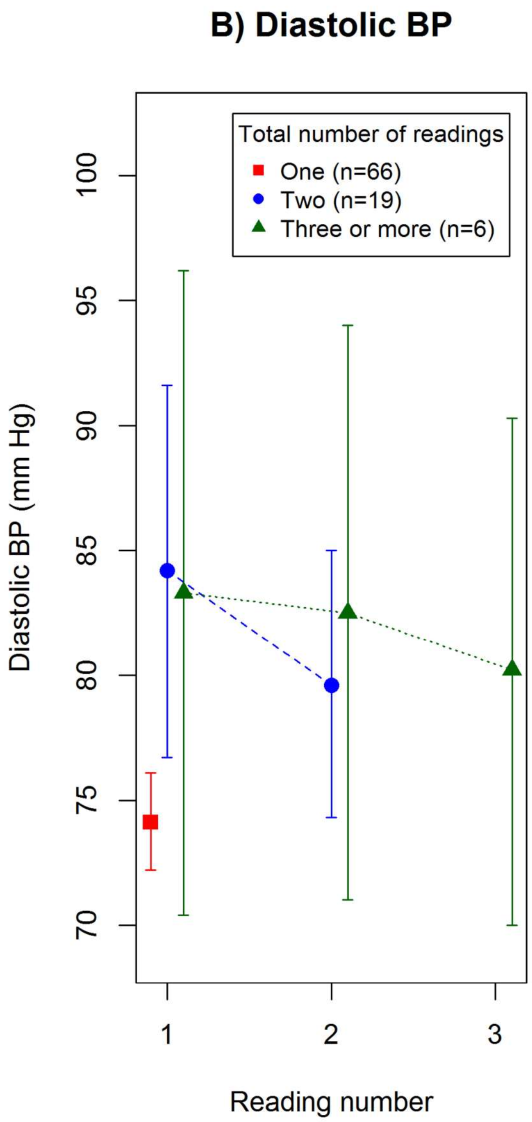

Fig.2: Mean blood pressure and 95% confidence intervals by reading number in 91 participants with diabetes who reported a value for each blood pressure reading in the prospective survey

179x360mm (300 x 300 DPI)

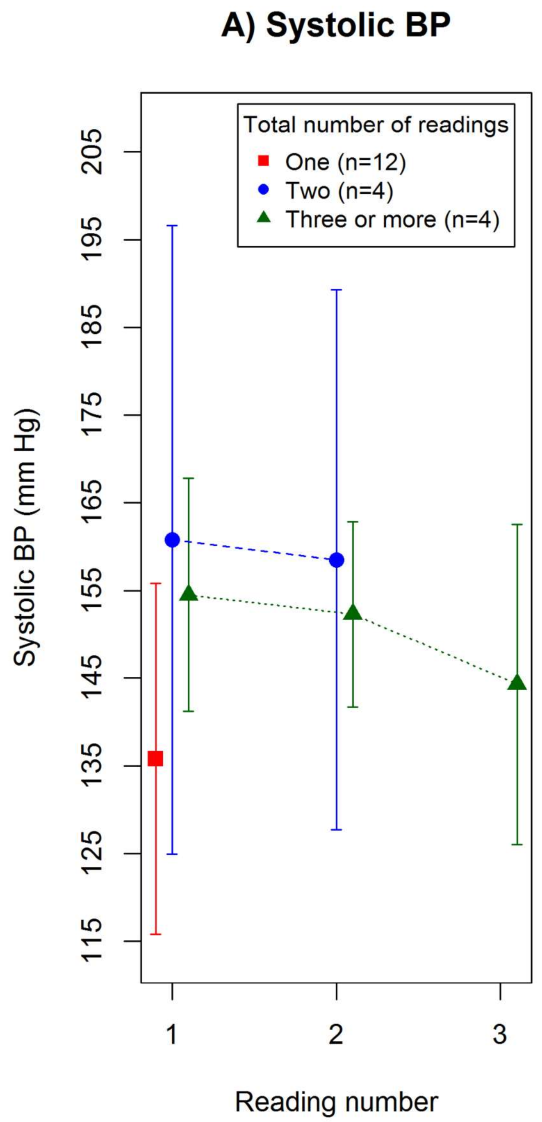

Fig. 3: Mean blood pressure and 95% confidence intervals by reading number in 20 participants without diabetes who reported a value for each blood pressure reading in the prospective survey

179x360mm (300 x 300 DPI)

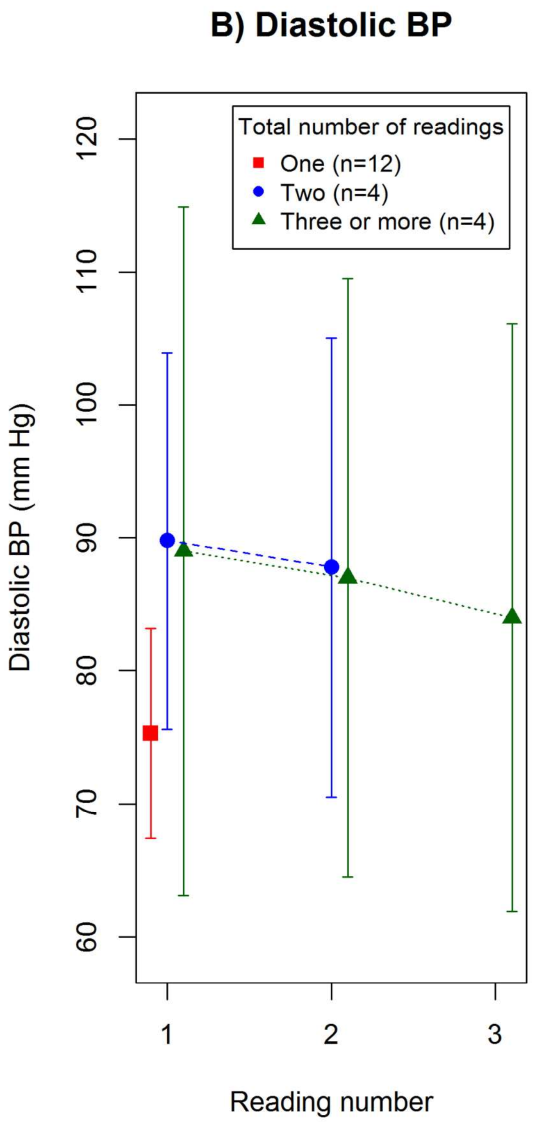

Fig. 3: Mean blood pressure and 95% confidence intervals by reading number in 20 participants without diabetes who reported a value for each blood pressure reading in the prospective survey

179x360mm (300 x 300 DPI)

Supplementary material

Initial survey

NUFFIELD DEPARTMENT OF
PRIMARY CARE
HEALTH SCIENCES

Nuffield Department of Primary Care Health Sciences • University of Oxford •
New Radcliffe House • Radcliffe Observatory Quarter • Woodstock Road •
Oxford OX2 6GG • www.phc.ox.ac.uk

Sarah Stevens
+44 (0)1865 289449 • sarah.stevens@phc.ox.ac.uk

Survey of blood pressure measurement in primary care (Survey 1)

Q1 Have you got high blood pressure or have you ever been told by your GP that you have high blood pressure?
- ☐ Yes. Go to Q2
- ☐ No. Go to Q3

Q2 Do you take any medication for your high blood pressure?
- ☐ Yes
- ☐ No

Q3 When was your last appointment with a GP or practice nurse at your general practice surgery?
- ☐ In the last week
- ☐ In the last month
- ☐ In the last 6 months
- ☐ In the last year
- ☐ More than a year ago
- ☐ Can't remember

Q4 Who was your last appointment at the surgery with?
- ☐ GP
- ☐ Nurse
- ☐ Can't remember

Q5 Was your blood pressure measured during your last appointment?
- ☐ Yes, by a GP. Go to Q6
- ☐ Yes, by a nurse. Go to Q6
- ☐ Yes, by yourself in the waiting room. Go to Q6.
- ☐ No. Go to Q9
- ☐ Can't remember

Q6 How many times was your blood pressure measured during the appointment? Count one measurement for each time the cuff on your arm went up.
- ☐ Once
- ☐ Twice
- ☐ Three times
- ☐ Four or more times
- ☐ Can't remember

Q7 Did your GP/ nurse tell you your blood pressure reading or discuss it with you?
- ☐ Yes
- ☐ No
- ☐ Can't remember

Q8 Do you know your blood pressure reading from the appointment?
- ☐ Yes. Go to Q9
- ☐ No. Go to Q10
- ☐ Wasn't told. Go to Q10
- ☐ Can't remember. Go to Q10

Q9 What was your blood pressure reading during the appointment?
(Your blood pressure consists of two numbers: systolic pressure and diastolic pressure. Systolic pressure is always the larger of the two numbers. For example if your blood pressure was 130/80 mmHg, 130 is the systolic reading and 80 is the diastolic reading.)

Systolic ___________ Diastolic ___________

Q10 During your last appointment, were you asked to measure your blood pressure yourself away from the practice e.g. at home?
- ☐ Yes
- ☐ No
- ☐ Can't remember

Q11 Have you ever had your blood pressure measured on both arms at the same appointment?
- ☐ Yes
- ☐ No
- ☐ Can't remember

Q12 Are you
- ☐ Male
- ☐ Female

Q13 How old are you? ____________ years

Q14 Do you currently smoke?
- ☐ Yes
- ☐ No

Q15 Do you take any medication for high cholesterol e.g. statins?
- ☐ Yes
- ☐ No

Q16 Have you ever been told by your GP that you have had any of the following conditions or a chronic condition?
(Tick all that apply)
- ☐ Diabetes (Type 1 or Type 2)
- ☐ Chronic kidney disease
- ☐ Stroke
- ☐ Heart attack
- ☐ Irregular heart beat (atrial fibrillation)
- ☐ Rheumatoid arthritis
- ☐ At high risk of having a heart attack or stroke
- ☐ Other. Please specify ___________________________
- ☐ None of the above

Q17 How did you hear about this survey?
- ☐ University of the Third Age
- ☐ Blood Pressure UK
- ☐ Other. Please specify ______________________

Q18 Please provide the first half of your postcode (e.g. ME19 4SH would be ME19. This will be used to analyse results by area only)

____

Q19 When do you expect your next appointment with your GP or nurse at your general practice surgery to be (confirmed or possible)?

- ☐ In the next week
- ☐ In the next two weeks
- ☐ In the next month
- ☐ In the next 3 months
- ☐ Don't know
- ☐ None scheduled

Q20 Would you be prepared to take part in a short (5 minute) follow-on survey after your next appointment? (The follow-on survey will ask further, similar questions about whether and how your blood pressure was measured at the appointment. This will include how many times your blood pressure was measured and what the level of your blood pressure was.)

- ☐ Yes (Go to Q21)
- ☐ No (END)

Q21 In order to take part in the follow-on survey, the researcher will need to contact you by email and needs your consent to do this. Please complete the following participant declaration:

a) I have read the study information above, had the opportunity to ask questions and have received satisfactory answers (Q1 of 8)

- ☐ Yes

b) I understand that this project has been reviewed by, and received ethics clearance through, the University of Oxford Central University Research Ethics Committee (Q2 of 8)

- ☐ Yes

c) I understand that my participation is voluntary and I am free to withdraw myself and my data at any time, without giving any reason, and without any adverse consequences (Q3 of 8)

- ☐ Yes

d) I understand who will have access to personal data provided (Q4 of 8)

- ☐ Yes

e) I understand that personal data will be stored according to the Data Protection Act and will only be accessed by researchers from the Nuffield Department of Primary Care Health Sciences, University of Oxford (Q5 of 8)

- ☐ Yes

f) I understand that the research will be written up and published peer-reviewed journals, presented at research meetings and published online as part of a student thesis, deposited both in print and online in the University of Oxford archives (Q6 of 8)

- ☐ Yes

g) I understand how to raise concerns or make a complaint (Q7 of 8)

- ☐ Yes

h) I agree to take part in the study (Q8 of 8)

- ☐ Yes

i) Please enter your email address (This will only be used to contact you via email)

____

Thank you for taking the time to complete this survey.

only

Prospective follow-up survey

UNIVERSITY OF OXFORD
NUFFIELD DEPARTMENT OF
PRIMARY CARE
HEALTH SCIENCES

Nuffield Department of Primary Care Health Sciences • University of Oxford • New Radcliffe House • Radcliffe Observatory Quarter • Woodstock Road • Oxford OX2 6GG • www.phc.ox.ac.uk

Sarah Stevens
+44 (0)1865 289449 • sarah.stevens@phc.ox.ac.uk

Survey of blood pressure measurement in primary care (Survey 2)

Q1 Please enter your email address (the email address given in survey 1. This will be used to match your responses from both surveys.)
Email address ______________

Q3 When was your last appointment with a GP or practice nurse at your general practice surgery?
- ☐ In the last week
- ☐ In the last month
- ☐ In the last 6 months
- ☐ In the last year
- ☐ More than a year ago
- ☐ Can't remember

Q4 Who was your last appointment at the surgery with?
- ☐ GP
- ☐ Nurse
- ☐ Can't remember

Q5 Was your blood pressure measured during your last appointment?
- ☐ Yes, by a GP. Go to Q6
- ☐ Yes, by a nurse. Go to Q6
- ☐ Yes, by yourself in the waiting room. Go to Q6.
- ☐ No. Go to Q9
- ☐ Can't remember

Q6 How many times was your blood pressure measured during the appointment? Count one measurement for each time the cuff on your arm went up.
- ☐ Once
- ☐ Twice
- ☐ Three times
- ☐ Four or more times
- ☐ Can't remember

Q7 Did your GP/ nurse tell you your blood pressure level or discuss it with you?
- ☐ Yes
- ☐ No
- ☐ Can't remember

Q8 Do you know your blood pressure reading from the appointment?
- ☐ Yes. Go to Q9
- ☐ No. Go to Q10
- ☐ Wasn't told. Go to Q10
- ☐ Can't remember. Go to Q10

Q9 What was your blood pressure reading during the appointment?
(Your blood pressure consists of two numbers: systolic pressure and diastolic pressure. Systolic pressure is always the larger of the two numbers. For example if your blood pressure was 130/80 mmHg, 130 is the systolic reading and 80 is the diastolic reading.)

	Systolic	Diastolic
First measurement	__________	__________
Second measurement	__________	__________
Third measurement	__________	__________

Q10 During your last appointment, were you asked to measure your blood pressure yourself away from the practice e.g. at home?

- ☐ Yes
- ☐ No
- ☐ Can't remember

Thank you for taking the time to complete this survey.

For peer review only

Table S1: Characteristics of participants completing initial and follow-up surveys

		Completed 1st survey (N=743)	Completed prospective survey (N=334)
**Characteristic**		Mean (SD) / N (%)	Mean (SD) / N (%)
**Male**		377 (50.7)	172 (51.5)
**Age**		57.4 (13.28)	59.3 (12.14)
**Current smoker**		48 (6.5)	25 (7.5)
**Hypertensive**		413 (55.6)	200 (59.9)
	**Antihypertensive medication**	353 (85.5)	173 (86.5)
**Diabetes**		623 (83.9)	279 (83.5)
**Previous CVD**		62 (8.3)	31 (9.3)
**Chronic kidney disease**		25 (3.4)	12 (3.6)
**Rheumatoid arthritis**		23 (3.1)	13 (3.9)
**Told at high risk of CVD**		55 (7.4)	30 (9.0)
**Region**			
	**North East**	18 (2.4)	9 (2.7)
	**North West**	285 (38.4)	125 (37.4)
	**Yorkshire & The Humber**	55 (7.4)	20 (6.0)
	**East Midlands**	23 (3.1)	8 (2.4)
	**West Midlands**	38 (5.1)	16 (4.8)
	**East of England**	42 (5.7)	28 (8.4)
	**South West**	103 (13.9)	49 (14.7)
	**South East**	93 (12.5)	57 (17.1)
	**London**	50 (6.7)	15 (4.5)
	**Other**	8 (1.1)	2 (0.6)
	**Unknown**	28 (3.8)	5 (1.5)

Table S2: Participant blood pressure (BP) measurement at their last general practice appointment in those with or without diabetes (initial survey results)

	Participants with diabetes (N=623)	Participants without diabetes (N=120)
**Did not have their BP measured**	209 (33.6%)	45 (37.5%)
**Had their BP measured by a GP**	119 (19.1%)	37 (30.8%)
**Had their BP measured by a nurse**	286 (45.9%)	35 (29.2%)
**Measured their BP themselves in the waiting room**	9 (1.4%)	3 (2.5%)

For peer review only

Table S3: Number of blood pressure measurements taken in participants with and without diabetes who could recall how many BP readings were taken (initial survey results)

Number of blood pressure measurements	Number of participants with diabetes (N=408)	Number of participants without diabetes (N=72)
**1**	250 (61.3%)	36 (50.0%)
**2**	124 (30.4%)	20 (27.8%)
**3 or more**	34 (8.3)	16 (22.2%)

Table S4: Blood pressure values by reading number and total number of readings in 91 participants with diabetes who reported a value for each blood pressure reading in the prospective survey

	**Total number of readings**	**Mean (95% CI) [Range]**		
		**Reading number**		
		**1**	**2**	**3**
**Systolic**	**1 (n=66)**	130.0 (126.6 to 133.3)<br>[110,176]	-<br>-	-<br>-
	**2 (n=19)**	139.4 (131.3 to 147.5)<br>[100,173]	131.7 (122.0 to 141.4)<br>[80, 173]	-<br>-
	**3 (n=6)**	133.3 (127.0 to 139.7)<br>[126, 140]	133.3 (127.0 to 139.6)<br>[126, 140]	131.8 (125.3 to 138.4)<br>[121, 139]
**Diastolic**	**1 (n=66)**	74.1 (72.2 to 76.1)<br>[60, 95]	-<br>-	-<br>-
	**2 (n=19)**	84.2 (76.7 to 91.6)<br>[65, 133]	79.6 (74.3 to 85.0)<br>[62, 112]	-<br>-
	**3 (n=6)**	83.3 (70.4 to 96.2)<br>[66, 96]	82.5 (71.0 to 94.0)<br>[69, 94]	80.2 (70.0 to 90.3)<br>[68, 91]

For peer review only

Table S5: Blood pressure values by reading number and total number of readings in 20 participants without diabetes reporting a value for all blood pressure readings in the prospective survey.

	Total number of readings	Mean (95% CI) [Range]		
		Reading number		
		1	2	3
**Systolic**	**1 (n=12)**	135.8 (115.8 to 155.8)<br>[110, 213]	-<br>-	-<br>-
	**2 (n=4)**	160.8 (124.9 to 196.6)<br>[137,181]	158.5 (127.7 to 189.3)<br>[137, 179]	-<br>-
	**3 (n=4)**	154.5 (141.2 to 167.8)<br>[147, 166]	152.3 (141.7 to 162.8)<br>[147, 162]	144.3 (126.0 to 162.5)<br>[128, 155]
**Diastolic**	**1 (n=12)**	75.3 (67.3 to 83.2)<br>[60, 100]	-<br>-	-<br>-
	**2 (n=4)**	89.8 (75.6 to 103.9)<br>[78, 98]	87.8 (70.5 to 105.0)<br>[78, 99]	-<br>-
	**3 (n=4)**	89.0 (63.1 to 114.9)<br>[70, 108]	87.0 (64.5 to 109.5)<br>[69, 101]	84.0 (61.9 to 106.1)<br>[65, 97]

For peer review only

Table S6: Blood pressure measurement by diabetes status in a random sample of respondents from unique postcode districts

	**Participants with diabetes** BP was measured in = 108/171 participants: 22 (20.4%) by a GP, 81 (75%) by a nurse and 5 (4.6%) by the patient		**Participants without diabetes** BP was measured in = 24/41 participants: 15 (62.5%) by a GP, 7 (29.2%) by a nurse and 2 (8.3%) by the patient	
**Number of times BP measured**	**N (%)**	**Asked to measure BP at home (N, %)**	**N (%)**	**Asked to measure BP at home (N, %)**
**Once**	58 (53.7%)	6 (10.3%)	17 (70.8%)	3 (17.6%)
**Twice**	34 (31.5%)	7 (20.6%)	2 (8.3%)	2 (100.0%)
**Three or more**	16 (14.8%)	5 (31.3%)	5 (20.8%)	3 (60.0%)

Table S7: Blood pressure values by reading number and total number of readings in a random sample of respondents from unique postcode districts (stratified by diabetes status, in those reporting all BP readings)

	**Total number of readings**	**Mean (SD)**		
		**Reading number**		
		**1**	**2**	**3**
**Participants with diabetes reporting all BP readings (N=49; 31 (63.3%) had BP measured according to guidelines)**				
**Systolic**	**1 (n=35)**	128.0 (16.3)	-	-
	**2 (n=10)**	135.1 (11.8)	125.1 (19.3)	-
	**3 (n=4)**	132.5 (6.5)	132.0 (6.5)	130.8 (7.7)
**Diastolic**	**1 (n=35)**	75.8 (8.7)	-	-
	**2 (n=10)**	85.8 (19.1)	80.9 (13.6)	-
	**3 (n=4)**	78.5 (12.3)	79.0 (11.7)	77.8 (10.8)
**Participants without diabetes reporting all BP readings (N=15; 8 (53.3%) had BP measured according to guidelines)**				
**Systolic**	**1 (n=9)**	137.0 (36.8)	-	-
	**2 (n=2)**	163.5 (24.7)	159.0 (15.6)	-
	**3 (n=4)**	154.5 (8.3)	152.3 (6.6)	144.3 (11.5)
**Diastolic**	**1 (n=9)**	78.3 (12.8)	-	-
	**2 (n=2)**	93.0 (7.1)	89.0 (14.1)	-
	**3 (n=4)**	89.0 (6.3)	87.0 (14.1)	84.0 (13.9)

Table S8: Likelihood of having BP measured multiple times or being asked to monitor BP at home, according to patient and practitioner characteristics in patients with and without diabetes from in a random sample of responses from unique postcode districts

	Likelihood of multiple BP measurements (n (%) in each group) (difference [95% confidence interval])	Likelihood of being asked to monitor BP at home (n (%) in each group) (difference [95% confidence interval])
**In patients with diabetes**		
**If the patient is hypertensive vs. normotensive**	31/55 (56.4%) vs. 19/53 (35.9%) difference = 20.5% [2.1 to 38.9%]	14/88 (15.9%) vs. 10/83 (12.1%) difference = 3.9% [-6.5 to 14.2%]
**If the patient has treated hypertension vs. untreated hypertension**	27/48 (56.3%) vs. 4/7 (57.1%) difference = 0.9% [-38.4 to 40.1%]	13/75 (17.3%) vs. 1/13 (7.7%) difference = 9.6% [-7.2 to 26.5%]
**If BP was measured by a GP vs. a nurse**	12/22 (54.5%) vs. 36/81 (44.4%) difference = 10.1% [-13.4 to 33.6%]	7/22 (31.8%) vs. 9/81 (11.1%) difference = 20.7% [0.1 to 41.3%]
**In patients without diabetes**		
**If the patient is hypertensive vs. normotensive**	6/19 (31.6%) vs. 1/5 (20.0%) difference = 11.6% [-29.2 to 52.4%]	11/31 (35.5%) vs. 0/10 (0.0%) difference = 35.5% [18.6% to 52.3%]
**If the patient has treated hypertension vs. untreated hypertension**	5/15 (33.3%) vs. 1/4 (25.0%) difference = 8.3% [-40.3 to 57.0%]	7/23 (30.4%) vs. 4/8 (50.0%) difference = 19.6% [-19.9 to 59.0%]
**If BP was measured by a GP vs. a nurse**	5/15 (33.3%) vs. 2/7 (28.6%) difference = 4.8% [-36.3 to 45.9%]	6/15 (40.0%) vs. 2/7 (28.6%) difference = 11.4% [-30.2 to 53.1%]

For peer review only

Table S9: Blood pressure measurement by diabetes status, excluding possible duplicate submissions

	**Participants with diabetes**<br>BP was measured in = 172/263 participants: 36 (20.9%) by a GP, 130 (75.6%) by a nurse and 6 (3.5%) by the patient		**Participants without diabetes**<br>BP was measured in = 33/54 participants: 21 (63.6%) by a GP, 10 (30.3%) by a nurse and 2 (6.1%) by the patient	
**Number of times BP measured**	**N (%)**	**Asked to measure BP at home (N, %)**	**N (%)**	**Asked to measure BP at home (N, %)**
**Once**	100 (58.1%)	10 (10.0%)	22 (66.6%)	5 (22.7%)
**Twice**	50 (29.1%)	11 (22.0%)	5 (12.2%)	3 (60.0%)
**Three or more**	22 (12.8%)	7 (31.8%)	6 (18.2%)	3 (50.0%)

Table S10: Blood pressure values by reading number and total number of readings excluding possible duplicate submissions (stratified by diabetes status, in those reporting all BP readings)

	**Total number of readings**	**Mean (SD)**		
		**Reading number**		
		**1**	**2**	**3**
**Participants with diabetes reporting all BP readings (N=91; 58 (63.7%) had BP measured according to guidelines)**				
**Systolic**	**1 (n=66)**	130.0 (13.5)	-	-
	**2 (n=19)**	139.4 (16.8)	131.7 (20.1)	-
	**3 (n=6)**	133.3 (6.1)	133.3 (6.0)	131.8 (6.2)
**Diastolic**	**1 (n=66)**	74.1 (7.9)	-	-
	**2 (n=19)**	84.2 (15.4)	79.6 (11.1)	-
	**3 (n=6)**	83.3 (12.3)	82.5 (10.9)	80.2 (9.7)
**Participants without diabetes reporting all BP readings (N=20; 12 (60.0%) had BP measured according to guidelines)**				
**Systolic**	**1 (n=12)**	135.8 (31.5)	-	-
	**2 (n=4)**	160.8 (22.5)	158.5 (19.4)	-
	**3 (n=4)**	154.5 (8.3)	152.3 (6.7)	144.3 (11.5)
**Diastolic**	**1 (n=12)**	75.3 (12.5)	-	-
	**2 (n=4)**	89.8 (8.9)	87.8 (10.8)	-
	**3 (n=4)**	89.0 (16.3)	87.0 (14.1)	84.0 (13.9)

For peer review only

Table S11: Likelihood of having BP measured multiple times or being asked to monitor BP at home, according to patient and practitioner characteristics in patients with and without diabetes, excluding possible duplicate submissions

	**Likelihood of multiple BP measurements (n (%) in each group) (difference [95% confidence interval])**	**Likelihood of being asked to monitor BP at home (n (%) in each group) (difference [95% confidence interval])**
**In patients with diabetes**		
**If the patient is hypertensive vs. normotensive**	45/98 (45.9%) vs. 27/74 (36.5%)<br>difference = 9.4% [-5.3 to 24.2%]	24/153 (15.7%) vs. 11/110 (10.0%)<br>difference = 5.7% [-2.4 to 13.7%]
**If the patient has treated hypertension vs. untreated hypertension**	39/88 (44.3%) vs. 6/10 (60.0%)<br>difference = 15.7% [-16.4 to 47.8%]	22/135 (16.3%) vs. 2/18 (11.1%)<br>difference = 5.2% [-10.6 to 21.0%]
**If BP was measured by a GP vs. a nurse**	16/36 (44.4%) vs. 54/130 (41.5%)<br>difference = 2.9% [-15.4 to 21.2%]	11/36 (30.6%) vs. 15/130 (11.5%)<br>difference = 19.0% [3.0 to 35.0%]
**In patients without diabetes**		
**If the patient is hypertensive vs. normotensive**	9/27 (33.3%) vs. 2/6 (33.3%)<br>difference = 0.0% [-41.7 to 41.7%]	14/40 (35.0%) vs. 0/14 (0.0%)<br>difference = 35.0% [20.2 to 49.8%]
**If the patient has treated hypertension vs. untreated hypertension**	8/22 (36.4%) vs. 1/5 (20.0%)<br>difference = 16.4% [-24.1 to 56.8%]	9/31 (29.0%) vs. 5/9 (55.6%)<br>difference = 26.5% [-9.7 to 62.7%]
**If BP was measured by a GP vs. a nurse**	9/21 (42.9%) vs 2/10 (20.0%)<br>difference = 22.9% [-9.7 to 55.5%]	7/21 (33.3%) vs 4/10 (40.0%)<br>difference = 6.7% [-29.8 to 43.1%]

For peer review only

STROBE 2007 (v4) Statement—Checklist of items that should be included in reports of *cross-sectional studies*

Section/Topic	Item #	Recommendation	Reported on page #
**Title and abstract**	1	(*a*) Indicate the study's design with a commonly used term in the title or the abstract	1
		(*b*) Provide in the abstract an informative and balanced summary of what was done and what was found	2
**Introduction**			
Background/rationale	2	Explain the scientific background and rationale for the investigation being reported	3
Objectives	3	State specific objectives, including any prespecified hypotheses	3
**Methods**			
Study design	4	Present key elements of study design early in the paper	4
Setting	5	Describe the setting, locations, and relevant dates, including periods of recruitment, exposure, follow-up, and data collection	4
Participants	6	(*a*) Give the eligibility criteria, and the sources and methods of selection of participants	4
Variables	7	Clearly define all outcomes, exposures, predictors, potential confounders, and effect modifiers. Give diagnostic criteria, if applicable	4
Data sources/ measurement	8*	For each variable of interest, give sources of data and details of methods of assessment (measurement). Describe comparability of assessment methods if there is more than one group	4/ Supplement
Bias	9	Describe any efforts to address potential sources of bias	*4*
Study size	10	Explain how the study size was arrived at	4
Quantitative variables	11	Explain how quantitative variables were handled in the analyses. If applicable, describe which groupings were chosen and why	4
Statistical methods	12	(*a*) Describe all statistical methods, including those used to control for confounding	4
		(*b*) Describe any methods used to examine subgroups and interactions	4
		(*c*) Explain how missing data were addressed	5
		(*d*) If applicable, describe analytical methods taking account of sampling strategy	N/A
		(*e*) Describe any sensitivity analyses	4
**Results**			

For peer review only

Participants	13*	(a) Report numbers of individuals at each stage of study—eg numbers potentially eligible, examined for eligibility, confirmed eligible, included in the study, completing follow-up, and analysed	5/ Figure 1
		(b) Give reasons for non-participation at each stage	Figure 1
		(c) Consider use of a flow diagram	Figure 1
Descriptive data	14*	(a) Give characteristics of study participants (eg demographic, clinical, social) and information on exposures and potential confounders	Table 1/ Table S1
		(b) Indicate number of participants with missing data for each variable of interest	5/Table 1/ S1
Outcome data	15*	Report numbers of outcome events or summary measures	5
Main results	16	(*a*) Give unadjusted estimates and, if applicable, confounder-adjusted estimates and their precision (eg, 95% confidence interval). Make clear which confounders were adjusted for and why they were included	5/ Table 2/ Figures 2 and 3
		(*b*) Report category boundaries when continuous variables were categorized	N/A
		(*c*) If relevant, consider translating estimates of relative risk into absolute risk for a meaningful time period	N/A
Other analyses	17	Report other analyses done—eg analyses of subgroups and interactions, and sensitivity analyses	5/Supplement
**Discussion**			
Key results	18	Summarise key results with reference to study objectives	6
Limitations	19	Discuss limitations of the study, taking into account sources of potential bias or imprecision. Discuss both direction and magnitude of any potential bias	6
Interpretation	20	Give a cautious overall interpretation of results considering objectives, limitations, multiplicity of analyses, results from similar studies, and other relevant evidence	7/8
Generalisability	21	Discuss the generalisability (external validity) of the study results	7
**Other information**			
Funding	22	Give the source of funding and the role of the funders for the present study and, if applicable, for the original study on which the present article is based	8

*Give information separately for cases and controls in case-control studies and, if applicable, for exposed and unexposed groups in cohort and cross-sectional studies.

Note: An Explanation and Elaboration article discusses each checklist item and gives methodological background and published examples of transparent reporting. The STROBE checklist is best used in conjunction with this article (freely available on the Web sites of PLoS Medicine at http://www.plosmedicine.org/, Annals of Internal Medicine at http://www.annals.org/, and Epidemiology at http://www.epidem.com/). Information on the STROBE Initiative is available at www.strobe-statement.org.

For peer review only

BMJ Open

Current practice of usual clinic blood pressure measurement in people with and without diabetes: a survey and prospective "mystery shopper" study in UK primary care

Journal:	*BMJ Open*
Manuscript ID	bmjopen-2017-020589.R1
Article Type:	Research
Date Submitted by the Author:	30-Jan-2018

Complete List of Authors:	Stevens, Sarah; University of Oxford, Nuffield Department of Primary Care Health Sciences<br>McManus, Richard; University of Oxford, Dept of Primary Care Health Sciences<br>Stevens, Richard; University of Oxford, Nuffield Dept Primary Care Health Sciences

**Primary Subject Heading**:	Cardiovascular medicine
Secondary Subject Heading:	Evidence based practice, General practice / Family practice, Health services research, Epidemiology, Diabetes and endocrinology
Keywords:	PRIMARY CARE, Hypertension < CARDIOLOGY, General diabetes < DIABETES & ENDOCRINOLOGY, Protocols & guidelines < HEALTH SERVICES ADMINISTRATION & MANAGEMENT, Quality in health care < HEALTH SERVICES ADMINISTRATION & MANAGEMENT

SCHOLARONE™ Manuscripts

Title: Current practice of usual clinic blood pressure measurement in people with and without diabetes: a survey and prospective "mystery shopper" study in UK primary care

S L Stevens[1], R J McManus[1], R J Stevens[1]

1. Nuffield Department of Primary Care Health Sciences, University of Oxford, Radcliffe Observatory Quarter,Woodstock Road,Oxford,OX2 6GG

Corresponding author: Sarah Stevens, sarah.stevens@phc.ox.ac.uk

Manuscript word count: 3334

For peer review only

Abstract

Objectives: Hypertension trials and epidemiological studies use multiple clinic blood pressure (BP) measurements at each visit. Repeat measurement is also recommended in international guidance, however little is known about how BP is measured routinely. This is important for individual patient management and because routinely recorded readings form part of research databases. We aimed to determine the current practice of BP measurement during routine general practice appointments.

Design: (1) An online cross-sectional survey and (2) A prospective “mystery shopper” study where patients agreed to report how BP was measured during their next appointment.

Setting: Primary care

Participants: Patient charity/ involvement group members completing an online survey between July 2015 and January 2016. 334 participants completed the prospective study (51.5% male, mean age = 59.3 years) of which 279 (83.5%) had diabetes.

Primary outcome: Proportion of patients having BP measured according to guidelines.

Results: 217 participants with (183) and without diabetes (34) had their BP measured at their last appointment. BP was measured in line with UK guidance in 63.7% and 60.0% of participants with and without diabetes respectively. Initial pressures were significantly higher in those who had their BP measured more than once compared to only once (p=0.0.016/ 0.089 systolic and p<0.001/=0.022 diastolic, in patients with/without diabetes respectively).

Conclusions: Current practice of routine BP measurement in UK primary care is often concordant with guidelines for repeat measurement. Further studies are required to confirm findings in broader populations, to confirm when a third repeat reading is obtained routinely and to assess adherence to other aspects of BP measurement guidance.

Strengths and limitations of this study

- This survey has a novel “mystery-shopper” design, minimising biases that may be introduced by self-reported practitioner behaviour.
- We have examined how adherence to guidelines varies according to patient characteristics, whereas previous studies have taken a healthcare professional view.
- The use of an online survey may have resulted in an under-representation of some groups, such as the very elderly.
- Larger studies are required to confirm our findings with respect to second and third blood pressure readings.

For peer review only - http://bmjopen.bmj.com/site/about/guidelines.xhtml

Introduction

Measurement of blood pressure (BP) is carried out in general practice by healthcare professionals on a daily basis. Such measurement is important for the diagnosis and management of hypertension, a major risk factor for cardiovascular disease (CVD) in both the general population,(1) and even more so in those with diabetes.(2) Hypertension trials and major epidemiological studies typically measure clinic BP using strict protocols on two to three times per visit in most cases.(3) For example the SPRINT(4) and ACCORD(5) trials used the mean of three readings taken automatically to guide treatment. Repeated measurement protocols are also recommended in the UK,(6) European,(7) and North American(8) hypertension guidelines. For example, current UK guidance states that BP should be re-measured if it is initially high, or if two measurements differ substantially, with out-of-office monitoring recommended in those with sustained high BP in clinic.(6)This reflects concerns that in many patients, clinic BP readings, particularly initial readings, may be systematically higher than BP during usual daily activities.(9)

Many factors can affect the accuracy of BP measurement and the number of measurements used can influence estimates of BP control.(10,11) Measurement practices may also vary depending on the focus of the consultation or patient characteristics and recorded blood pressures may also be influenced by incentive schemes such as the UK Quality and Outcomes Framework.(12,13) Potential differences between primary study protocols and clinical practice have implications for the generalisability and implementation of research findings. For example, SPRINT found that treatment to a systolic BP target of 120 mmHg resulted in fewer cardiovascular events compared to a target of 140 mmHg in low risk patients.(4) However others have argued that mean automatically measured BP of 120 mmHg may correspond to a routine measurement of 135-140 mmHg(14) and ACCORD (conducted in patients with diabetes) failed to show an effect of intensive treatment.(5)

Furthermore, increasing numbers of observational studies in electronic healthcare databases rely on routinely collected BP measurements. In particular, the recommended cardiovascular risk calculator in the UK, QRISK2,(15) was derived using such data. It is important to understand how blood pressures recorded in these databases were obtained, in order to reliably compare observational database and primary study results.

However, little is known about how BP is measured in routine practice. A 2006 survey of UK general practitioners' (GPs') adherence to hypertension guideline recommendations relied on self-reported data and did not ask about the use of repeat measurements.(16) Other European studies have focussed on whether implementation of lifestyle or treatment changes adheres to guidelines,(17) or reasons for non-adherence.(18) These studies assume that an accurate BP reading is obtained initially and ignore the specifics of BP measurement. We therefore sought to determine the current practice of BP measurement during routine appointments in UK primary care, focusing on when repeat clinic and home BP measurements are obtained.

Patients and methods

We conducted an online survey of patients, followed by a prospective survey of primary care consultations.

Online survey

For peer review only - http://bmjopen.bmj.com/site/about/guidelines.xhtml

An online survey was advertised through charities and patient involvement groups ("University of the Third Age", "Blood Pressure UK", "Citizen Scientist", "Patients Active in Research", "Call for Participants" and "Research for the Future (Help BEAT Diabetes)") between 23rd July 2015 and 24th January 2016. Respondents anonymously reported basic demographic and health information, if and how many times their BP was measured at their last appointment and (recall permitting) their last BP reading (Supplement). Respondents were also asked about recommendations to monitor their BP at home.

Prospective study

Participants completing the online survey were invited to take part in a prospective study. They were told the study would ask similar questions to those already asked about their BP after their next primary care appointment. Those wishing to take part gave explicit consent, provided an email address and were asked when they expected their next appointment to be. After the anticipated time of this appointment, a link to an online questionnaire was emailed to participants. This asked whether BP was measured at the appointment; and if so, how many times, and (recall permitting) for up to three systolic and diastolic BP values (Supplement). The questionnaire was open from 23rd July 2015 to 16th June 2016.Two patient representatives helped design the study materials and three were asked to pilot the survey websites to test functionality. The study was approved by the Medical Sciences Interdivisional Research Ethics Committee, University of Oxford (reference: MS-IDREC-C1-2015-095).

Statistical Analysis

The prospective study was powered to estimate the proportion of people having their BP measured once or multiple times, in line with guidelines at the 95% confidence level with an accuracy of +/-5%. Assuming a proportion of 10%, 139 respondents who had had their BP measured was required.(19)

Demographic and clinical history data were summarised using means and standard deviations or proportions. Mean BP was summarized with 95% confidence intervals and ranges. Respondents were classified as hypertensive if they answered yes to the question "Have you got high blood pressure or have you ever been told by your GP that you have high blood pressure?". Responses were assessed against NICE guidance and BP was deemed to have been measured according to guidelines if BP was measured; a) once and the reading was below 140/90 mmHg, b) twice if the initial reading was above 140/90 mmHg and the first two readings differed by less than 5 mmHg systolic, or c) three times if the first reading was above 140/90 mmHg and the first two readings differed by more than 5 mmHg systolic.(6) Respondents who had their BP measured more or less than guidance recommends were deemed not to have had their BP measured according to guidance. Proportions were compared using two-sided tests of proportions, under the assumption of large samples, at the 5% level. Due to an unexpectedly large proportion of participants with diabetes, a decision to stratify all prospective study analyses by patient diabetes status was made after data collection.

Since behaviour amongst professionals from the same practice may be similar, sensitivity analyses were carried out by randomly selecting one observation from each postcode district (assuming respondents from different districts are registered to distinct practices). We also conducted sensitivity analyses excluding prospective responses that were suspected of being duplicate

For peer review only - http://bmjopen.bmj.com/site/about/guidelines.xhtml

submissions of the same initial survey data. Analysis was conducted using Stata 14,(20) and R 3.3.1.(21)

Results

In total 2176 unique users visited the survey site, of whom 756 completed the initial online survey, with complete data available in 743 individuals (623 with diabetes, 83.9%). Consent for the prospective study was given by 593 participants and was completed by 334 participants (279 with diabetes, 83.5%) (Fig. 1). The characteristics of those completing the initial and prospective surveys were broadly similar (Table S1, Supplement).

Initial survey

Of the 743 people completing the first survey, 489 (65.8%) reported having had their BP measured at their last appointment: 156 (31.9% of 489) by a GP, 321 (65.6%) by a nurse and 12 (2.5%) in the waiting room. Most respondents (480/489, 98.2%) could recall how many BP readings were taken: 286 (59.6% of 480) one, 144 (30.0%) two and 50 (10.4%) three or more readings. Results stratified by diabetes status are given in the supplement (Tables S2 and S3). Only 88 patients (11.8%) recalled ever having their BP measured in both arms at any one previous appointment. Compared to normotensives (20/330, (6.7%)), respondents with a previous diagnosis of hypertension (68/413, (16.5%)) were more likely to report having had their BP measured in both arms at any appointment previously.

Prospective study

Baseline characteristics for those with and without diabetes completing the prospective study after a further GP appointment are given in Table 1. Of the 279 participants with diabetes completing the follow-up questionnaire, 183 (65.6%) had their BP measured at the appointment: 38 (20.8%) by a GP, 139 (76.0%) by a nurse and 6 (3.3%) by themselves in the waiting room. Of the 55 participants without diabetes, 34 (61.8%), had their BP measured: 21 (61.8%) by a GP, 11 (32.4%) by a nurse and 2 (5.9%) by themselves in the waiting room.

Participants with diabetes

Of the 183 participants with diabetes who had their BP measured, 91 (49.7%) could recall a value for all of the BP readings given. Fifty-eight respondents (63.7%, 95% CI [53.0 to 73.6%]) had their BP measured according to guidelines. Mean BP values by reading number are presented graphically in Fig. 2A (systolic) and Fig. 2B (diastolic, see Table S4 for raw data). Initial systolic and diastolic blood pressures were lower in participants who had their BP measured only once than in those who had it measured two or more times (mean systolic difference = 8.0 mm Hg, 95% CI [1.2 to 14.5 mm Hg], p=0.016 and mean diastolic difference = 9.9 mm Hg, 95% CI [5.1 to 14.6 mm Hg], p<0.001).

The proportion of participants with diabetes who had their BP measured multiple times was similar regardless of hypertensive or treatment status, or measurement personnel (Table 2, top left). However, they were more likely to be asked to monitor their BP at home when BP was measured by a GP compared to a nurse (Table 2, top right). Those who had their BP measured once, twice and three or more times, were asked to monitor their BP at home in 10/109 (9.2%, 95% CI [3.8 to 14.6%]), 11/51 (21.6%, 95% CI [10.3% to 32.9%]) and 7/23 (30.4%, 95% CI [11.6 to 49.2%]) cases respectively.

For peer review only - http://bmjopen.bmj.com/site/about/guidelines.xhtml

Participants without diabetes

Of the 34 participants without diabetes who had their BP measured, 20 (58.8%) could recall a value for all of the BP readings given. Twelve respondents (60.0%, 95% CI [36.1% to 80.9%]) had their BP measured according to guidelines. Mean BP values by reading number are presented graphically in Fig. 3A (systolic) and Fig. 3B (diastolic, see Table S5 for raw data). Patterns of repeat BP measurement were similar to those observed in participants with diabetes, although numbers in this group were smaller. Initial systolic blood pressures were non-significantly lower in participants who had their BP measured only once than in those who had it measured two or more times (mean systolic difference = 21.8 mm Hg, 95% CI [-3.7 to 47.3 mm Hg], p=0.089). However, a significant difference was observed for diastolic pressure (mean diastolic difference = 14.1 mm Hg, 95% CI [2.3 to 26.0 mm Hg], p=0.022).

The proportion of participants without diabetes who had their BP measured multiple times was similar regardless of hypertensive or treatment status, or measurement personnel (Table 2, bottom left). However, those with hypertension were more likely to be asked to monitor their BP at home compared to normotensives (Table 2, bottom right). Those who had their BP measured once, twice and three or more times, were asked to monitor their BP at home in 5/23 (21.7%, 95% CI [4.9 to 38.6%]), 3/5 (60.0%, 95% CI [17.1% to 100.0%]) and 3/6 (50.0%, 95% CI [10.0 to 90.0%]) cases respectively.

Sensitivity analyses

Results were similar after randomly sampling responses from unique postcode districts (Tables S6 to S8) or when excluding prospective responses suspected of being duplicate submissions of the initial survey data (Tables S9 to S11).

Discussion

Summary

This study has shown that a second BP measurement at clinic visit is more likely to be taken if the initial BP measurement is high. This is consistent with UK guidelines. However, there is no clear evidence that the decision to take a third measurement follows guidelines. The recommendation that a third measurement be taken only when the first two are discrepant (first measurement above threshold but second below threshold for diagnosis of hypertension) was not obviously reflected in our data, although confidence intervals are wide. Although the majority of this evidence relates to people with diabetes, similar BP measurement practices were observed in those without diabetes.

Strengths and limitations

The patient centred nature of this study has allowed us to see into the consulting room for the first time and to determine how BP is measured in "real life", in those with and without diabetes. Previous studies have taken a healthcare professional view.(16)

Our online survey was limited by the use of convenience mechanisms for recruitment, and like many internet surveys with no known denominator, these results should be interpreted with caution. The use of an online system itself may have resulted in an under-representation of some groups, such as the very elderly.(22) For the prospective study, we were able to obtain "mystery shopper" type data

For peer review only - http://bmjopen.bmj.com/site/about/guidelines.xhtml

on more than two hundred GP and nurse appointments without potentially influencing the appointment through direct observation by a researcher. To our knowledge, these data are unique. The lower numbers of respondents without diabetes could limit generalisability if health care professionals follow protocols less carefully in patients without additional cardiovascular risk factors.(23) Recruitment through patient involvement groups may have also resulted in over-representation of patients who are actively engaged with their healthcare, and due to phenomenon such as the inverse-care law,(24) may receive better quality (guideline adherent) care. However, since our aim was to study the behaviours of healthcare professionals, it is unclear how any biases at the patient-level will have translated into biases at the healthcare professional level. Furthermore, previous research regarding current practice of BP self-monitoring, showed similar results using both convenience and representative sampling of professionals.(25)

Although our mechanism of data collection, asking patients to study the behaviour of their health care practitioners, has the limitations discussed above, we chose our "mystery shopper" approach over several other study designs. For a full discussion of the study designs considered see Stevens PhD thesis, 2017.(26) Briefly, studies based on alternative methodologies, such as practitioner self-report or direct observation, would have been subject to selection bias among practitioners, the Hawthorne effect, and reporting bias and we have avoided these biases through our novel design.

Self-reported BP readings may have been subject to rounding error, digit preference or recall error. This introduces uncertainty into some analyses concerning blood pressure values, but the number of measurements taken, is likely to be recalled with greater accuracy, especially in the prospective study. Guidance covers many factors affecting the accuracy of BP measurement, such as the use an appropriately sized cuff, but such factors are less easily assessed by patients and we chose to limit the focus of this study in order to maximise response rates. The type and accuracy of devices used in UK general practice has been studied previously,(27) but further direct observation of clinicians is warranted to determine if other aspects of BP measurement guidance is followed.

Fewer than anticipated participants provided all BP readings and therefore we could only estimate the proportion of people with diabetes having their BP measured according to guidelines with an error of +/- 10% (compared to an original target of +/-5%). However, we have demonstrated important differences (for example in first systolic BP readings) despite this. Although we have demonstrated that BP is measured in line with guidance in the majority of cases, this was driven by a large number of participants with low BP who had their BP measured only once. Larger studies would be required to confirm our findings, particularly with respect to second and third readings and in those without diabetes.

Many factors, other than the initial BP value, can influence the decision to measure BP multiple times including previous measurement of BP in clinic or at home and the presence of cardiovascular disease or cardiovascular risk factors. Such factors may explain the considerable variability in BP measurement practices observed in some specific patient examples. Although we have addressed key factors such as diabetes, hypertension and treatment status, future research could explore behaviour in other subgroups. Furthermore, we did not ask respondents about the primary reason for their consultation which may have influenced BP measurement and this also requires further study.

Comparison with existing literature

For peer review only - http://bmjopen.bmj.com/site/about/guidelines.xhtml
For peer review only

A previous review(18) of barriers to hypertension awareness and treatment found that professionals were concerned about the accuracy of individual clinic BP readings. Our results support the idea that professionals treat single readings with caution, particularly those above the diagnostic threshold which require further action (e.g. in the form of treatment change). Although numbers were smaller, results suggest that this caution also extends to high BP sustained over two readings. Previous research suggests that recording of blood pressure may be influenced by specific BP related targets in the UK's Quality and Outcomes Framework,(12) and hence routine practice in other healthcare systems, with different incentive schemes, may differ. Despite agreement between current practice and guidelines, GPs may be better advised to use multiple readings more widely,(28) to ensure comparability with BP monitoring studies and detection of masked hypertension which affects approximately 19% of adults.(29)

The finding that patients are more likely to be monitored at home if they have high clinic pressures or hypertension is consistent with results from a recent practitioner survey in Canada, (30) where guidance is similar.(31) A recent survey of general practices in the South West of England found that only 1 in 10 GP practices were not following current guidelines for the use of home and ambulatory BP monitoring in the diagnosis of hypertension,(32) which is also consistent with our results in that guidance appears to be followed in most cases.

GPs were more likely to recommend home monitoring than nurses in those with diabetes. It is difficult to interpret this finding as it may reflect the primary reason for consultation, with certain tasks (such as diabetes reviews) performed primarily by nurses. Current guidance for BP management in diabetes recommends that high BP is confirmed at subsequent appointments, rather than through home monitoring.(33) Hence this finding, which importantly was not replicated in those without diabetes, may be explained if many of those with diabetes had annual review appointments. Overall, few patients were encouraged to monitor their BP at home, although it is likely that around 31% of patients were already self-monitoring based on previous UK survey data.(34)

Implications for research and practice

Our findings indicate that routine BP measurement does not reflect the strict measurement protocols in primary research studies. This has implications for patient care if results from primary research studies cannot be appropriately translated into guidance for routine care (e.g. in the form of adjusted treatment targets). Users of electronic healthcare databases should also be aware of the potential for recording biases(12) which may dilute the observed effect of BP on outcomes and may extend to other biological factors subject to measurement error.

The current practice of BP measurement will, reassuringly, detect white coat hypertension but may not identify those with masked effects (where BP is higher outside of the clinic). This could potentially result in missed diagnoses and sub-optimal treatment. One solution which would not increase workload is use of the PROOF-BP tool which was developed by two of the authors with colleagues.(35) This combines factors associated with home-clinic BP differences with BP readings to identify which patients may exhibit masked or white coat effects and would benefit most from out-of-office monitoring. It accurately identifies hypertension in 93% of cases and is more accurate than current diagnostic guidelines.(36) Implementation of this tool could improve detection of masked effects and avoid unnecessary out-of-office monitoring.

For peer review only - http://bmjopen.bmj.com/site/about/guidelines.xhtml

Less than 1 in 5 participants with hypertension reported having their BP measured in both arms at a single appointment previously. Large differences between arms are associated with vascular disease and mortality.(37) These results suggest little change since 13% of GPs said they adhered to this recommendation a decade ago.(16) Other recent estimates suggest that around half of practices measure BP in both arms as part of the diagnostic procedure,(32) which, although more optimistic, further demonstrates room for improvement. Barriers to such improvement may include practitioner discordance with guidance (previously only 30% agreed with the recommendation), or a lack of a suitable devices.(38)

The results of this study provide a preliminary insight into how BP is measured routinely and indicate that repeat BP measurements are taken in line with guidelines but not with strict study protocols. The impact of these differences on patient care requires further investigation.

Funding

This project is funded by the National Institute for Health Research School for Primary Care Research (NIHR SPCR). The views expressed are those of the author(s) and not necessarily those of the NHS, the NIHR or the Department of Health.

Conflicts of interest

RM has received BP monitoring equipment for research purposes from Lloyds Pharmacies and Omron.

Contributions

All authors conceived and designed the study. SS was responsible for the management of the study and carried out the statistical analysis. SS drafted the paper which RS and RM then contributed to.

Collaborators

We thank computer programmers David Judge and Luis Castello (Nuffield Department of Primary Care Health Sciences, University of Oxford) who developed and managed the survey sites. We also thank patient representatives Derek Shaw, Valerie Keston-Hole and others for their help developing the study materials.

Data sharing

No additional data are available.

For peer review only

For peer review only - http://bmjopen.bmj.com/site/about/guidelines.xhtml

References

1. Lewington S, Clarke R, Qizilbash N, Peto R, Collins R. Age-specific relevance of usual blood pressure to vascular mortality: A meta-analysis of individual data for one million adults in 61 prospective studies. Lancet [Internet]. 2002;360(9349):1903–13. Available from: http://www.scopus.com/inward/record.url?eid=2-s2.0-0037079309&partnerID=40&md5=6b6019ec4f94d9603b79803a2fbcd31e

2. Adler AI, Stratton IM, Neil HAW, Yudkin JS, Matthews DR, Cull CA, et al. Association of systolic blood pressure with macrovascular and microvascular complications of type 2 diabetes (UKPDS 36): prospective observational study. BMJ [Internet]. 2000 [cited 2017 Jun 6];321(7258). Available from: http://www.bmj.com/content/321/7258/412

3. Giorgini P, Weder AB, Jackson EA, Brook RD. A review of blood pressure measurement protocols among hypertension trials: Implications for “evidence-based” clinical practice. J Am Soc Hypertens. 2014;8(9):670–6.

4. The SPRINT Research Group. A Randomized Trial of Intensive versus Standard Blood-Pressure Control. N Engl J Med [Internet]. 2015 Nov 26 [cited 2016 Sep 23];373(22):2103–16. Available from: http://www.nejm.org/doi/10.1056/NEJMoa1511939

5. The ACCORD Study Group. Effects of Intensive Blood-Pressure Control in Type 2 Diabetes Mellitus. N Engl J Med [Internet]. 2010 Apr 29 [cited 2017 Jun 6];362(17):1575–85. Available from: http://www.nejm.org/doi/abs/10.1056/NEJMoa1001286

6. National Institute for Health and Care Excellence. Hypertension: Clinical management of primary hypertension in adults (NICE CG127). National Institute for Health and Care Excellence; 2011.

7. Mancia G, Fagard R, Narkiewicz K, Redon J, Zanchetti A, Böhm M, et al. 2013 ESH/ESC guidelines for the management of arterial hypertension: the Task Force for the Management of Arterial Hypertension of the European Society of Hypertension (ESH) and of the European Society of Cardiology (ESC). Eur Heart J [Internet]. 2013 Jul 14 [cited 2014 Jul 10];34(28):2159–219. Available from: http://eurheartj.oxfordjournals.org/content/early/2013/06/13/eurheartj.eht151

8. James PA, Oparil S, Carter BL, Cushman WC, Dennison-Himmelfarb C, Handler J, et al. Evidence-Based Guideline for the Management of High Blood Pressure in Adults. Jama [Internet]. 2013;1097(5):1–14. Available from: http://jama.jamanetwork.com/article.aspx?articleid=1791497%5Cnhttp://jama.jamanetwork.com/article.aspx?doi=10.1001/jama.2013.284427

9. Pickering TG, Gerin W, Schwartz AR. What is the white-coat effect and how should it be measured? Blood Press Monit [Internet]. 2002;7(6):329–33. Available from: http://ovidsp.ovid.com/ovidweb.cgi?T=JS&PAGE=reference&D=emed5&NEWS=N&AN=2003053147

10. Birkett NJ. The effect of alternative criteria for hypertension on estimates of prevalence and control. J Hypertens. 1997;15:237–44.

11. McAlister FA, Straus SE. Evidence based treatment of hypertension: Measurement of blood presssure: an evidence based review. BMJ. 2001;322(7291):908–11.

12. Carey IM, Nightingale CM, DeWilde S, Harris T, Whincup PH, Cook DG. Blood pressure recording bias during a period when the Quality and Outcomes Framework was introduced. J

For peer review only - http://bmjopen.bmj.com/site/about/guidelines.xhtml

Hum Hypertens [Internet]. 2009 Nov 12 [cited 2016 Aug 4];23(11):764–70. Available from: http://www.nature.com/doifinder/10.1038/jhh.2009.18

13. Doran T, Fullwood C, Gravelle H, Reeves D, Kontopantelis E, Hiroeh U, et al. Pay-for-performance programs in family practices in the United Kingdom. N Engl J Med [Internet]. 2006 Jul 27 [cited 2015 Jun 10];355(4):375–84. Available from: http://www.ncbi.nlm.nih.gov/pubmed/16870916

14. Schiffrin EL, Calhoun DA, Flack JM. Do We Need a New Definition of Hypertension After SPRINT? Am J Hypertens [Internet]. 2016 Oct [cited 2016 Sep 23];29(10):1127–9. Available from: http://www.ncbi.nlm.nih.gov/pubmed/27334926

15. Hippisley-Cox J, Coupland C, Vinogradova Y, Robson J, Minhas R, Sheikh A, et al. Predicting cardiovascular risk in England and Wales: prospective derivation and validation of QRISK2. BMJ [Internet]. 2008;336(7659):1475–82. Available from: http://www.pubmedcentral.nih.gov/articlerender.fcgi?artid=2440904&tool=pmcentrez&rendertype=abstract

16. Heneghan C, Perera R, Mant D, Glasziou P. Hypertension guideline recommendations in general practice: awareness, agreement, adoption, and adherence. Br J Gen Pract [Internet]. 2007 Dec [cited 2015 Feb 4];57(545):948–52. Available from: http://www.pubmedcentral.nih.gov/articlerender.fcgi?artid=2084133&tool=pmcentrez&rendertype=abstract

17. Meneton P, Ricordeau P, Weill A, Tuppin P, Samson S, Allemand H, et al. Evaluation of the agreement between guidelines and initial antihypertensive drug treatment using a national health care reimbursement database. J Eval Clin Pract [Internet]. 2012 Jun [cited 2016 May 5];18(3):623–9. Available from: http://www.ncbi.nlm.nih.gov/pubmed/21276142

18. Khatib R, Schwalm J-D, Yusuf S, Haynes RB, McKee M, Khan M, et al. Patient and healthcare provider barriers to hypertension awareness, treatment and follow up: a systematic review and meta-analysis of qualitative and quantitative studies. PLoS One [Internet]. 2014 Jan 15 [cited 2016 Apr 16];9(1):e84238. Available from: http://journals.plos.org/plosone/article?id=10.1371/journal.pone.0084238

19. Bartlett JE, Kotrlik JW, Higgins CC. Organizational Research: Determining Appropriate Sample Size in Survey Research . Inf Technol Learn Perform J. 2001;19(1).

20. StataCorp. Stata Statistical Software: Release 14. College Station, Texas, USA: StataCorp LP; 2015.

21. R Core Team. R: A language and environment for statistical computing. [Internet]. R Foundation for Statistical Computing, Vienna, Austria; 2016. Available from: https://www.r-project.org/

22. Office for National Statistics. Internet users in the UK : 2016 [Internet]. 2016. Available from: https://www.ons.gov.uk/businessindustryandtrade/itandinternetindustry/bulletins/internetusers/2016

23. Stamler J, Vaccaro O, Neaton JD, Wentworth D. Diabetes, Other Risk Factors, and 12-Yr Cardiovascular Mortality for Men Screened in clusively of people with the Multiple Risk Factor Intervention Trial. Diabetes Care. 1993;16(2):434–44.

24. Mercer SW, Guthrie B, Furler J, Watt GCM, Hart JT. Multimorbidity and the inverse care law in primary care. BMJ [Internet]. 2012 [cited 2017 Apr 21];344:e4152. Available from:

For peer review only

For peer review only - http://bmjopen.bmj.com/site/about/guidelines.xhtml

http://eprints.gla.ac.uk

25. Fletcher BR, Hinton L, Bray EP, Hayen A, Hobbs FR, Mant J, et al. Self-monitoring blood pressure in patients with hypertension: an internet-based survey of UK GPs. Br J Gen Pract [Internet]. 2016;66(652):e831–7. Available from: http://bjgp.org/cgi/doi/10.3399/bjgp16X687037

26. Stevens SL. Improving cardiovascular risk prediction through more accurate and alternative methods of blood pressure measurement. University of Oxford; 2017.

27. A'Court C, Stevens R, Sanders S, Ward A, McManus R, Heneghan C. Type and accuracy of sphygmomanometers in primary care: a cross-sectional observational study. Br J Gen Pract [Internet]. 2011 Sep [cited 2016 Jun 22];61(590):e598-603. Available from: http://www.ncbi.nlm.nih.gov/pubmed/22152749

28. Keenan K, Hayen A, Neal BC, Irwig L. Long term monitoring in patients receiving treatment to lower blood pressure: analysis of data from placebo controlled randomised controlled trial. BMJ. 2009;338.

29. Verberk WJ, Kessels AGH, de Leeuw PW. Prevalence, Causes, and Consequences of Masked Hypertension: A Meta-analysis. Am J Hypertens [Internet]. 2008 Sep 1 [cited 2017 Dec 20];21(9):969–75. Available from: https://academic.oup.com/ajh/article-lookup/doi/10.1038/ajh.2008.221

30. Kaczorowski J, Myers MG, Gelfer M, Dawes M, Mang EJ, Berg A, et al. How do family physicians measure blood pressure in routine clinical practice? Can Fam Physician [Internet]. 2017 [cited 2017 Apr 24];63(3). Available from: http://www.cfp.ca/content/63/3/e193

31. Leung AA, Nerenberg K, Daskalopoulou SS, McBrien K, Zarnke KB, Dasgupta K, et al. Hypertension Canada's 2016 Canadian Hypertension Education Program Guidelines for Blood Pressure Measurement, Diagnosis, Assessment of Risk, Prevention, and Treatment of Hypertension. Can J Cardiol. 2016;32(5):569–88.

32. Mejzner N, Clark CE, Smith LF, Campbell JL. Trends in the diagnosis and management of hypertension: repeated primary care survey in South West England. Br J Gen Pract [Internet]. 2017 [cited 2017 May 4];67(658). Available from: http://bjgp.org/content/67/658/e306

33. National Institute for Health and Care Excellence. NICE CG28. Type 2 diabetes in adults: management [Internet]. NICE; 2015 [cited 2017 Dec 21]. Available from: https://www.nice.org.uk/guidance/ng28/chapter/1-Recommendations#blood-pressure-management-2

34. Baral-Grant S, Haque MS, Nouwen A, Greenfield SM, McManus RJ. Self-Monitoring of Blood Pressure in Hypertension: A UK Primary Care Survey. Int J Hypertens [Internet]. 2012 [cited 2017 May 12];2012:582068. Available from: http://www.ncbi.nlm.nih.gov/pubmed/22013510

35. Sheppard JP, Fletcher B, Gill P, Martin U, Roberts N, McManus RJ. Predictors of the Home-Clinic Blood Pressure Difference: A Systematic Review and Meta-Analysis. Am J Hypertens [Internet]. 2016 May [cited 2016 Aug 11];29(5):614–25. Available from: http://ajh.oxfordjournals.org/lookup/doi/10.1093/ajh/hpv157

36. Sheppard JP, Stevens R, Gill P, Martin U, Godwin M, Hanley J, et al. Predicting Out-of-Office Blood Pressure in the Clinic (PROOF-BP). Hypertension [Internet]. 2016 May [cited 2016 Aug 11];67(5):941–50. Available from:

For peer review only

For peer review only - http://bmjopen.bmj.com/site/about/guidelines.xhtml

http://hyper.ahajournals.org/lookup/doi/10.1161/HYPERTENSIONAHA.115.07108

37. Clark CE, Taylor RS, Shore AC, Ukoumunne OC, Campbell JL. Association of a difference in systolic blood pressure between arms with vascular disease and mortality: a systematic review and meta-analysis. Lancet [Internet]. 2012 Mar 10 [cited 2016 May 5];379(9819):905–14. Available from: http://www.sciencedirect.com/science/article/pii/S0140673611617108

38. A'Court C, Sheppard J, Greenhalgh T. Blood pressure measurement: a call to arms. Br J Gen Pract. 2016;66(652).

For peer review only

For peer review only - http://bmjopen.bmj.com/site/about/guidelines.xhtml

Table 1: Characteristics of participants completing the prospective survey with and without diabetes

		Participants with diabetes (N=279)	Participants without diabetes (N=55)
**Characteristic**		Mean (SD) / N (%)	Mean (SD) / N (%)
**Male**		157 (56.3)	15 (27.3)
**Age**		59.0 (12.1)	60.3 (12.7)
**Current smoker**		21 (7.5)	4 (7.3)
**Hypertensive**		159 (57.0)	41 (74.6)
	**Antihypertensive medication**	141 (88.7)	32 (78.0)
**Previous CVD**		29 (10.4)	2 (3.6)
**Chronic kidney disease**		11 (3.9)	1 (1.8)
**Rheumatoid arthritis**		12 (4.3)	1 (1.8)
**Told at high risk of CVD**		26 (9.3)	4 (7.3)
**Region**			
	**North East**	9 (3.2)	0 (0.0)
	**North West**	111 (39.8)	14 (25.5)
	**Yorkshire & The Humber**	19 (6.8)	1 (1.8)
	**East Midlands**	6 (2.2)	2 (3.6)
	**West Midlands**	13 (4.7)	3 (5.5)
	**East of England**	22 (7.9)	6 (10.9)
	**South West**	40 (14.3)	9 (16.4)
	**South East**	42 (15.1)	15 (27.3)
	**London**	13 (4.7)	2 (3.6)
	**Other**	0 (0.0)	2 (3.6)
	**Unknown**	4 (1.4)	1 (1.8)

For peer review only

For peer review only - http://bmjopen.bmj.com/site/about/guidelines.xhtml

Table 2: Likelihood of having BP measured multiple times or being asked to monitor BP at home, according to patient and practitioner characteristics (stratified by diabetes status)

	Likelihood of multiple BP measurements (n (%) in each group) (difference [95% confidence interval])	Likelihood of being asked to monitor BP at home (n (%) in each group) (difference [95% confidence interval])
In participants with diabetes		
If the participant was hypertensive vs. normotensive	46/103 (44.7%) vs. 28/80 (35.0%) difference = 9.7% [-4.5 to 23.9%])	24/159 (15.1%) vs. 13/120 (10.8%) difference=4.3% [-3.6 to 12.1%]
If the participant had treated hypertension vs. untreated hypertension	40/93 (43.0%) vs. 6/10 (60.0%) difference = -17.0% [-49.0 to 15.0%]	22/141 (15.6%) vs. 2/18 (11.1%) difference = 4.5% [-11.2 to 20.2%]
If BP was measured by a GP vs. a nurse	16/38 (42.1%) vs. 56/139 (40.3%) difference = 1.8% [-15.9 to 19.5%]	11/38 (28.9%) vs. 15/139 (10.8%) difference = 18.2% [2.8 to 33.5%]
In participants without diabetes		
If the participant was hypertensive vs. normotensive	2/6 (33.3%) vs. 9/28 (32.1%) difference = 1.2% [-40.3 to 42.7%]	14/41 (34.1%) vs. 0/14 (0.0%) difference = 34.1% [19.6 to 48.7%]
If the participant had treated hypertension vs. untreated hypertension	8/23 (34.8%) vs. 1/5 (20.0%) difference = 14.8% [-25.3 to 54.9%]	9/32 (28.1%) vs. 5/9 (55.6%) difference = -27.4% [-63.4% to 8.6%]
If BP was measured by a GP vs. a nurse	9/21 (42.9%) vs. 2/11 (18.2%) difference = 24.7% [-6.4 to 55.8%]	7/21 (33.3%) vs. 4/11 (36.4%) difference = -3.0% [-37.9 to 31.8%]

For peer review only

Fig. 1: Study flowchart

Fig.2: Mean blood pressure and 95% confidence intervals by reading number in 91 participants with diabetes who reported a value for each blood pressure reading in the prospective survey

Fig. 3: Mean blood pressure and 95% confidence intervals by reading number in 20 participants without diabetes who reported a value for each blood pressure reading in the prospective survey

For peer review only

For peer review only - http://bmjopen.bmj.com/site/about/guidelines.xhtml

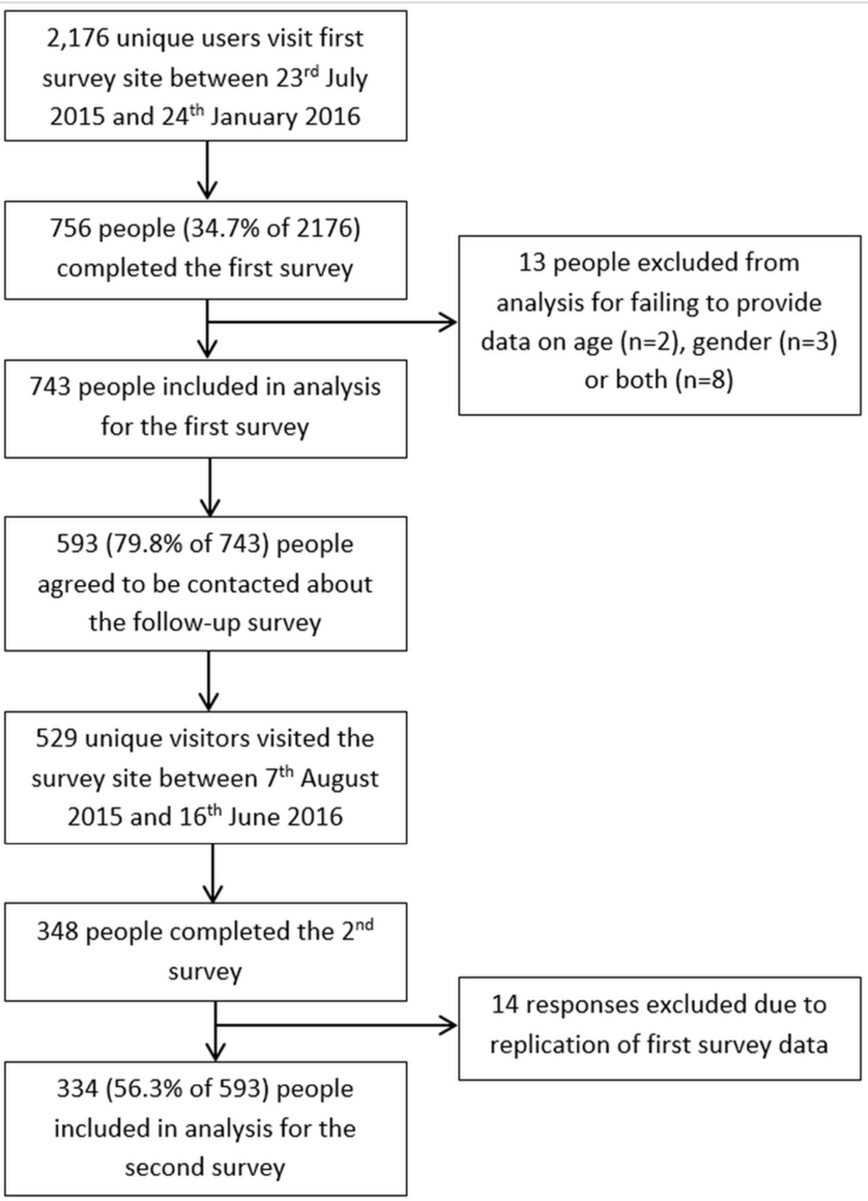

Fig. 1: Study flowchart

41x57mm (600 x 600 DPI)

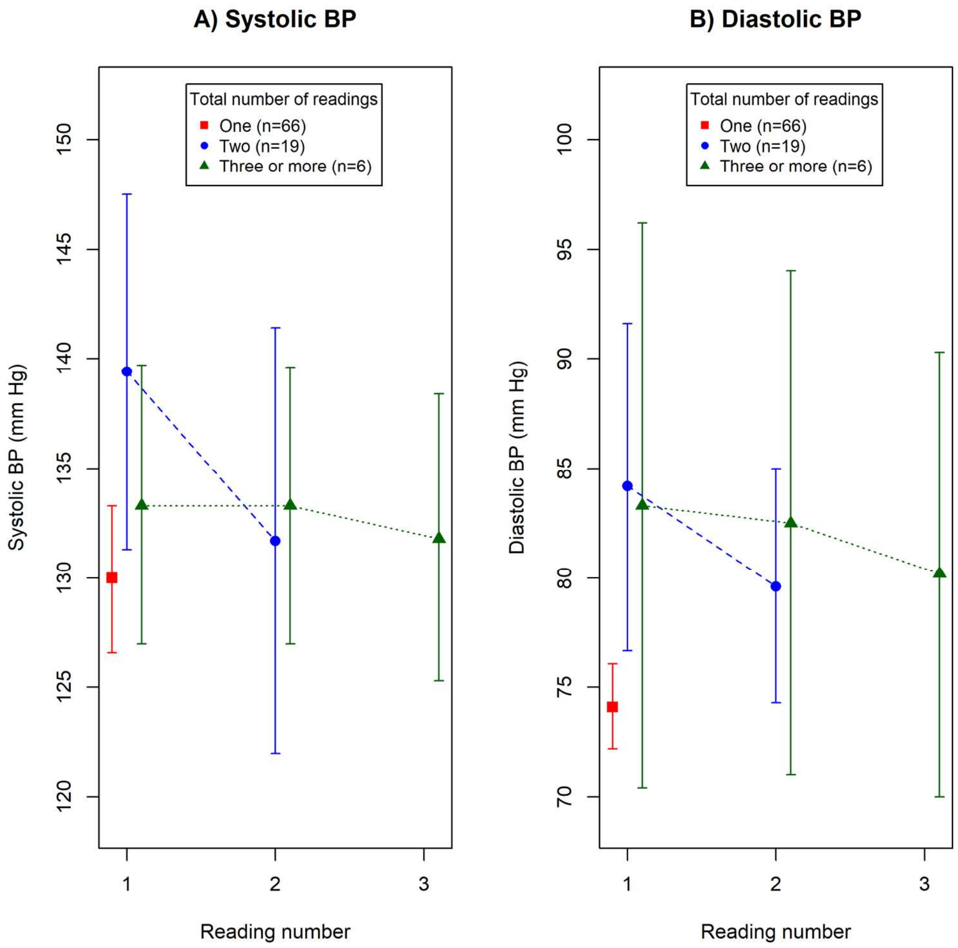

Fig.2: Mean blood pressure and 95% confidence intervals by reading number in 91 participants with diabetes who reported a value for each blood pressure reading in the prospective survey

179x179mm (300 x 300 DPI)

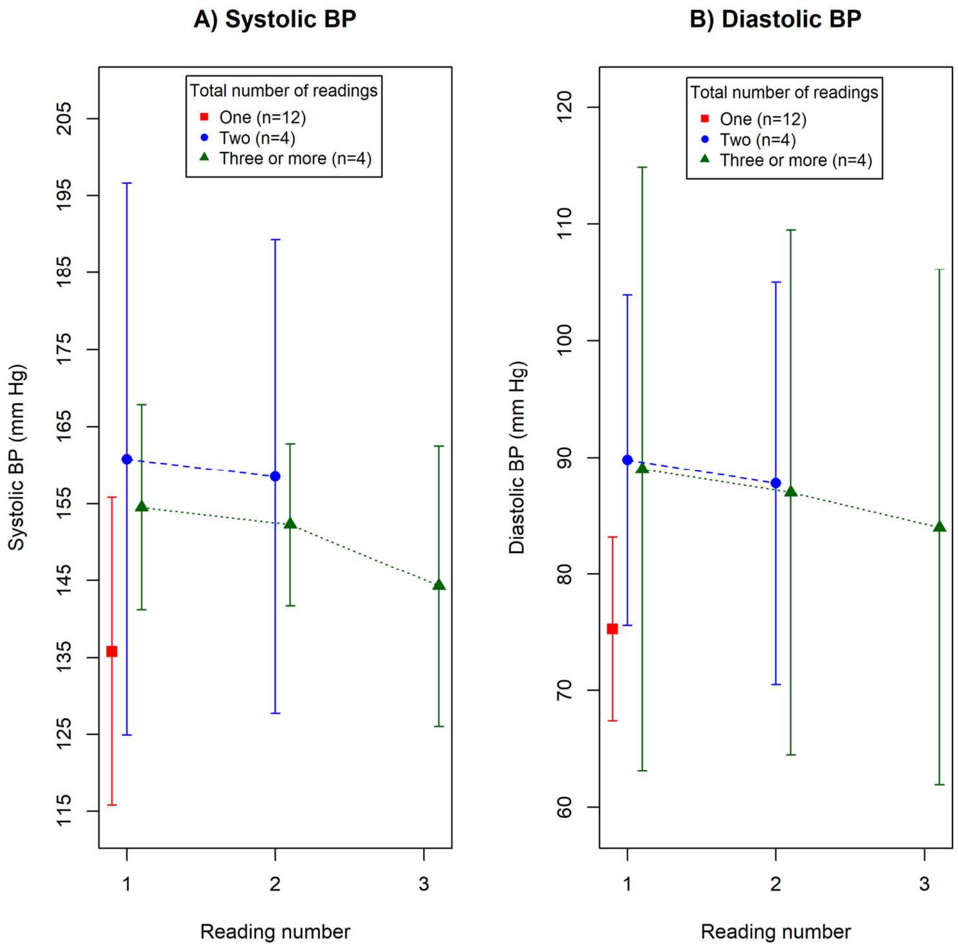

Fig. 3: Mean blood pressure and 95% confidence intervals by reading number in 20 participants without diabetes who reported a value for each blood pressure reading in the prospective survey

179x179mm (300 x 300 DPI)

Supplementary material

Initial survey

UNIVERSITY OF OXFORD
NUFFIELD DEPARTMENT OF PRIMARY CARE HEALTH SCIENCES

Nuffield Department of Primary Care Health Sciences • University of Oxford • New Radcliffe House • Radcliffe Observatory Quarter • Woodstock Road • Oxford OX2 6GG • www.phc.ox.ac.uk

Sarah Stevens
+44 (0)1865 289449 • sarah.stevens@phc.ox.ac.uk

Survey of blood pressure measurement in primary care (Survey 1)

Q1 Have you got high blood pressure or have you ever been told by your GP that you have high blood pressure?
- ☐ Yes. Go to Q2
- ☐ No. Go to Q3

Q2 Do you take any medication for your high blood pressure?
- ☐ Yes
- ☐ No

Q3 When was your last appointment with a GP or practice nurse at your general practice surgery?
- ☐ In the last week
- ☐ In the last month
- ☐ In the last 6 months
- ☐ In the last year
- ☐ More than a year ago
- ☐ Can't remember

Q4 Who was your last appointment at the surgery with?
- ☐ GP
- ☐ Nurse
- ☐ Can't remember

Q5 Was your blood pressure measured during your last appointment?
- ☐ Yes, by a GP. Go to Q6
- ☐ Yes, by a nurse. Go to Q6
- ☐ Yes, by yourself in the waiting room. Go to Q6.
- ☐ No. Go to Q9
- ☐ Can't remember

Q6 How many times was your blood pressure measured during the appointment? Count one measurement for each time the cuff on your arm went up.
- ☐ Once
- ☐ Twice
- ☐ Three times
- ☐ Four or more times
- ☐ Can't remember

Q7 Did your GP/ nurse tell you your blood pressure reading or discuss it with you?
- ☐ Yes
- ☐ No
- ☐ Can't remember

Q8 Do you know your blood pressure reading from the appointment?
- ☐ Yes. Go to Q9
- ☐ No. Go to Q10
- ☐ Wasn't told. Go to Q10
- ☐ Can't remember. Go to Q10

Q9 What was your blood pressure reading during the appointment?
(Your blood pressure consists of two numbers: systolic pressure and diastolic pressure. Systolic pressure is always the larger of the two numbers. For example if your blood pressure was 130/80 mmHg, 130 is the systolic reading and 80 is the diastolic reading.)

Systolic ____________ Diastolic ____________

Q10 During your last appointment, were you asked to measure your blood pressure yourself away from the practice e.g. at home?
- ☐ Yes
- ☐ No
- ☐ Can't remember

Q11 Have you ever had your blood pressure measured on both arms at the same appointment?
- ☐ Yes
- ☐ No
- ☐ Can't remember

Q12 Are you
- ☐ Male
- ☐ Female

Q13 How old are you? ____________ years

Q14 Do you currently smoke?
- ☐ Yes
- ☐ No

Q15 Do you take any medication for high cholesterol e.g. statins?
- ☐ Yes
- ☐ No

Q16 Have you ever been told by your GP that you have had any of the following conditions or a chronic condition? (Tick all that apply)
- ☐ Diabetes (Type 1 or Type 2)
- ☐ Chronic kidney disease
- ☐ Stroke
- ☐ Heart attack
- ☐ Irregular heart beat (atrial fibrillation)
- ☐ Rheumatoid arthritis
- ☐ At high risk of having a heart attack or stroke
- ☐ Other. Please specify ____________________________
- ☐ None of the above

Q17 How did you hear about this survey?
- ☐ University of the Third Age
- ☐ Blood Pressure UK
- ☐ Other. Please specify ______________________

Q18 Please provide the first half of your postcode (e.g. ME19 4SH would be ME19. This will be used to analyse results by area only)

____

Q19 When do you expect your next appointment with your GP or nurse at your general practice surgery to be (confirmed or possible)?

- ☐ In the next week
- ☐ In the next two weeks
- ☐ In the next month
- ☐ In the next 3 months
- ☐ Don't know
- ☐ None scheduled

Q20 Would you be prepared to take part in a short (5 minute) follow-on survey after your next appointment? (The follow-on survey will ask further, similar questions about whether and how your blood pressure was measured at the appointment. This will include how many times your blood pressure was measured and what the level of your blood pressure was.)

- ☐ Yes (Go to Q21)
- ☐ No (END)

Q21 In order to take part in the follow-on survey, the researcher will need to contact you by email and needs your consent to do this. Please complete the following participant declaration:

a) I have read the study information above, had the opportunity to ask questions and have received satisfactory answers (Q1 of 8)

☐ Yes

b) I understand that this project has been reviewed by, and received ethics clearance through, the University of Oxford Central University Research Ethics Committee (Q2 of 8)

☐ Yes

c) I understand that my participation is voluntary and I am free to withdraw myself and my data at any time, without giving any reason, and without any adverse consequences (Q3 of 8)

☐ Yes

d) I understand who will have access to personal data provided (Q4 of 8)

☐ Yes

e) I understand that personal data will be stored according to the Data Protection Act and will only be accessed by researchers from the Nuffield Department of Primary Care Health Sciences, University of Oxford (Q5 of 8)

☐ Yes

f) I understand that the research will be written up and published peer-reviewed journals, presented at research meetings and published online as part of a student thesis, deposited both in print and online in the University of Oxford archives (Q6 of 8)

☐ Yes

g) I understand how to raise concerns or make a complaint (Q7 of 8)

☐ Yes

h) I agree to take part in the study (Q8 of 8)

☐ Yes

i) Please enter your email address (This will only be used to contact you via email)

____

Thank you for taking the time to complete this survey.

only

Prospective follow-up survey

NUFFIELD DEPARTMENT OF
PRIMARY CARE
HEALTH SCIENCES

Nuffield Department of Primary Care Health Sciences • University of Oxford • New Radcliffe House • Radcliffe Observatory Quarter • Woodstock Road • Oxford OX2 6GG • www.phc.ox.ac.uk

Sarah Stevens
+44 (0)1865 289449 • sarah.stevens@phc.ox.ac.uk

Survey of blood pressure measurement in primary care (Survey 2)

Q1 Please enter your email address (the email address given in survey 1. This will be used to match your responses from both surveys.)

Email address _______________

Q3 When was your last appointment with a GP or practice nurse at your general practice surgery?

- ☐ In the last week
- ☐ In the last month
- ☐ In the last 6 months
- ☐ In the last year
- ☐ More than a year ago
- ☐ Can't remember

Q4 Who was your last appointment at the surgery with?

- ☐ GP
- ☐ Nurse
- ☐ Can't remember

Q5 Was your blood pressure measured during your last appointment?

- ☐ Yes, by a GP. Go to Q6
- ☐ Yes, by a nurse. Go to Q6
- ☐ Yes, by yourself in the waiting room. Go to Q6.
- ☐ No. Go to Q9
- ☐ Can't remember

Q6 How many times was your blood pressure measured during the appointment? Count one measurement for each time the cuff on your arm went up.

- ☐ Once
- ☐ Twice
- ☐ Three times
- ☐ Four or more times
- ☐ Can't remember

Q7 Did your GP/ nurse tell you your blood pressure level or discuss it with you?

- ☐ Yes
- ☐ No
- ☐ Can't remember

Q8 Do you know your blood pressure reading from the appointment?

- ☐ Yes. Go to Q9
- ☐ No. Go to Q10
- ☐ Wasn't told. Go to Q10
- ☐ Can't remember. Go to Q10

Q9 What was your blood pressure reading during the appointment?
(Your blood pressure consists of two numbers: systolic pressure and diastolic pressure. Systolic pressure is always the larger of the two numbers. For example if your blood pressure was 130/80 mmHg, 130 is the systolic reading and 80 is the diastolic reading.)

	Systolic	Diastolic
First measurement	___________	___________
Second measurement	___________	___________
Third measurement	___________	___________

Q10 During your last appointment, were you asked to measure your blood pressure yourself away from the practice e.g. at home?

- ☐ Yes
- ☐ No
- ☐ Can't remember

Thank you for taking the time to complete this survey.

For peer review only

Table S1: Characteristics of participants completing initial and follow-up surveys

		Completed 1st survey (N=743)	Completed prospective survey (N=334)
**Characteristic**		Mean (SD) / N (%)	Mean (SD) / N (%)
**Male**		377 (50.7)	172 (51.5)
**Age**		57.4 (13.28)	59.3 (12.14)
**Current smoker**		48 (6.5)	25 (7.5)
**Hypertensive**		413 (55.6)	200 (59.9)
	**Antihypertensive medication**	353 (85.5)	173 (86.5)
**Diabetes**		623 (83.9)	279 (83.5)
**Previous CVD**		62 (8.3)	31 (9.3)
**Chronic kidney disease**		25 (3.4)	12 (3.6)
**Rheumatoid arthritis**		23 (3.1)	13 (3.9)
**Told at high risk of CVD**		55 (7.4)	30 (9.0)
**Region**			
	**North East**	18 (2.4)	9 (2.7)
	**North West**	285 (38.4)	125 (37.4)
	**Yorkshire & The Humber**	55 (7.4)	20 (6.0)
	**East Midlands**	23 (3.1)	8 (2.4)
	**West Midlands**	38 (5.1)	16 (4.8)
	**East of England**	42 (5.7)	28 (8.4)
	**South West**	103 (13.9)	49 (14.7)
	**South East**	93 (12.5)	57 (17.1)
	**London**	50 (6.7)	15 (4.5)
	**Other**	8 (1.1)	2 (0.6)
	**Unknown**	28 (3.8)	5 (1.5)

For peer review only

Table S2: Participant blood pressure (BP) measurement at their last general practice appointment in those with or without diabetes (initial survey results)

	Participants with diabetes (N=623)	Participants without diabetes (N=120)
**Did not have their BP measured**	209 (33.6%)	45 (37.5%)
**Had their BP measured by a GP**	119 (19.1%)	37 (30.8%)
**Had their BP measured by a nurse**	286 (45.9%)	35 (29.2%)
**Measured their BP themselves in the waiting room**	9 (1.4%)	3 (2.5%)

Table S3: Number of blood pressure measurements taken in participants with and without diabetes who could recall how many BP readings were taken (initial survey results)

Number of blood pressure measurements	Number of participants with diabetes (N=408)	Number of participants without diabetes (N=72)
**1**	250 (61.3%)	36 (50.0%)
**2**	124 (30.4%)	20 (27.8%)
**3 or more**	34 (8.3)	16 (22.2%)

Table S4: Blood pressure values by reading number and total number of readings in 91 participants with diabetes who reported a value for each blood pressure reading in the prospective survey

	Total number of readings	Mean (95% CI) [Range]		
		Reading number		
		1	2	3
**Systolic**	**1 (n=66)**	130.0 (126.6 to 133.3) [110,176]	- -	- -
	**2 (n=19)**	139.4 (131.3 to 147.5) [100,173]	131.7 (122.0 to 141.4) [80, 173]	- -
	**3 (n=6)**	133.3 (127.0 to 139.7) [126, 140]	133.3 (127.0 to 139.6) [126, 140]	131.8 (125.3 to 138.4) [121, 139]
**Diastolic**	**1 (n=66)**	74.1 (72.2 to 76.1) [60, 95]	- -	- -
	**2 (n=19)**	84.2 (76.7 to 91.6) [65, 133]	79.6 (74.3 to 85.0) [62, 112]	- -
	**3 (n=6)**	83.3 (70.4 to 96.2) [66, 96]	82.5 (71.0 to 94.0) [69, 94]	80.2 (70.0 to 90.3) [68, 91]

For peer review only

Table S5: Blood pressure values by reading number and total number of readings in 20 participants without diabetes reporting a value for all blood pressure readings in the prospective survey.

	Total number of readings	Mean (95% CI) [Range]		
		Reading number		
		1	2	3
Systolic	1 (n=12)	135.8 (115.8 to 155.8) [110, 213]	- -	- -
	2 (n=4)	160.8 (124.9 to 196.6) [137,181]	158.5 (127.7 to 189.3) [137, 179]	- -
	3 (n=4)	154.5 (141.2 to 167.8) [147, 166]	152.3 (141.7 to 162.8) [147, 162]	144.3 (126.0 to 162.5) [128, 155]
Diastolic	1 (n=12)	75.3 (67.3 to 83.2) [60, 100]	- -	- -
	2 (n=4)	89.8 (75.6 to 103.9) [78, 98]	87.8 (70.5 to 105.0) [78, 99]	- -
	3 (n=4)	89.0 (63.1 to 114.9) [70, 108]	87.0 (64.5 to 109.5) [69, 101]	84.0 (61.9 to 106.1) [65, 97]

For peer review only

Table S6: Blood pressure measurement by diabetes status in a random sample of respondents from unique postcode districts

	**Participants with diabetes** BP was measured in = 108/171 participants: 22 (20.4%) by a GP, 81 (75%) by a nurse and 5 (4.6%) by the patient		**Participants without diabetes** BP was measured in = 24/41 participants: 15 (62.5%) by a GP, 7 (29.2%) by a nurse and 2 (8.3%) by the patient	
**Number of times BP measured**	**N (%)**	**Asked to measure BP at home (N, %)**	**N (%)**	**Asked to measure BP at home (N, %)**
**Once**	58 (53.7%)	6 (10.3%)	17 (70.8%)	3 (17.6%)
**Twice**	34 (31.5%)	7 (20.6%)	2 (8.3%)	2 (100.0%)
**Three or more**	16 (14.8%)	5 (31.3%)	5 (20.8%)	3 (60.0%)

Table S7: Blood pressure values by reading number and total number of readings in a random sample of respondents from unique postcode districts (stratified by diabetes status, in those reporting all BP readings)

	**Total number of readings**	**Mean (SD)**		
		**Reading number**		
		**1**	**2**	**3**
**Participants with diabetes reporting all BP readings (N=49; 31 (63.3%) had BP measured according to guidelines)**				
**Systolic**	**1 (n=35)**	128.0 (16.3)	-	-
	**2 (n=10)**	135.1 (11.8)	125.1 (19.3)	-
	**3 (n=4)**	132.5 (6.5)	132.0 (6.5)	130.8 (7.7)
**Diastolic**	**1 (n=35)**	75.8 (8.7)	-	-
	**2 (n=10)**	85.8 (19.1)	80.9 (13.6)	-
	**3 (n=4)**	78.5 (12.3)	79.0 (11.7)	77.8 (10.8)
**Participants without diabetes reporting all BP readings (N=15; 8 (53.3%) had BP measured according to guidelines)**				
**Systolic**	**1 (n=9)**	137.0 (36.8)	-	-
	**2 (n=2)**	163.5 (24.7)	159.0 (15.6)	-
	**3 (n=4)**	154.5 (8.3)	152.3 (6.6)	144.3 (11.5)
**Diastolic**	**1 (n=9)**	78.3 (12.8)	-	-
	**2 (n=2)**	93.0 (7.1)	89.0 (14.1)	-
	**3 (n=4)**	89.0 (6.3)	87.0 (14.1)	84.0 (13.9)

For peer review only

Table S8: Likelihood of having BP measured multiple times or being asked to monitor BP at home, according to patient and practitioner characteristics in patients with and without diabetes from in a random sample of responses from unique postcode districts

	**Likelihood of multiple BP measurements (n (%) in each group) (difference [95% confidence interval])**	**Likelihood of being asked to monitor BP at home (n (%) in each group) (difference [95% confidence interval])**
**In patients with diabetes**		
**If the patient is hypertensive vs. normotensive**	31/55 (56.4%) vs. 19/53 (35.9%) difference = 20.5% [2.1 to 38.9%]	14/88 (15.9%) vs. 10/83 (12.1%) difference = 3.9% [-6.5 to 14.2%]
**If the patient has treated hypertension vs. untreated hypertension**	27/48 (56.3%) vs. 4/7 (57.1%) difference = 0.9% [-38.4 to 40.1%]	13/75 (17.3%) vs. 1/13 (7.7%) difference = 9.6% [-7.2 to 26.5%]
**If BP was measured by a GP vs. a nurse**	12/22 (54.5%) vs. 36/81 (44.4%) difference = 10.1% [-13.4 to 33.6%]	7/22 (31.8%) vs. 9/81 (11.1%) difference = 20.7% [0.1 to 41.3%]
**In patients without diabetes**		
**If the patient is hypertensive vs. normotensive**	6/19 (31.6%) vs. 1/5 (20.0%) difference = 11.6% [-29.2 to 52.4%]	11/31 (35.5%) vs. 0/10 (0.0%) difference = 35.5% [18.6% to 52.3%]
**If the patient has treated hypertension vs. untreated hypertension**	5/15 (33.3%) vs. 1/4 (25.0%) difference = 8.3% [-40.3 to 57.0%]	7/23 (30.4%) vs. 4/8 (50.0%) difference = 19.6% [-19.9 to 59.0%]
**If BP was measured by a GP vs. a nurse**	5/15 (33.3%) vs. 2/7 (28.6%) difference = 4.8% [-36.3 to 45.9%]	6/15 (40.0%) vs. 2/7 (28.6%) difference = 11.4% [-30.2 to 53.1%]

For peer review only

Table S9: Blood pressure measurement by diabetes status, excluding possible duplicate submissions

	Participants with diabetes<br>BP was measured in = 172/263 participants: 36 (20.9%) by a GP, 130 (75.6%) by a nurse and 6 (3.5%) by the patient		Participants without diabetes<br>BP was measured in = 33/54 participants: 21 (63.6%) by a GP, 10 (30.3%) by a nurse and 2 (6.1%) by the patient	
**Number of times BP measured**	**N (%)**	**Asked to measure BP at home (N, %)**	**N (%)**	**Asked to measure BP at home (N, %)**
**Once**	100 (58.1%)	10 (10.0%)	22 (66.6%)	5 (22.7%)
**Twice**	50 (29.1%)	11 (22.0%)	5 (12.2%)	3 (60.0%)
**Three or more**	22 (12.8%)	7 (31.8%)	6 (18.2%)	3 (50.0%)

For peer review only

Table S10: Blood pressure values by reading number and total number of readings excluding possible duplicate submissions (stratified by diabetes status, in those reporting all BP readings)

	**Total number of readings**	**Mean (SD)**		
		**Reading number**		
		**1**	**2**	**3**
**Participants with diabetes reporting all BP readings (N=91; 58 (63.7%) had BP measured according to guidelines)**				
**Systolic**	**1 (n=66)**	130.0 (13.5)	-	-
	**2 (n=19)**	139.4 (16.8)	131.7 (20.1)	-
	**3 (n=6)**	133.3 (6.1)	133.3 (6.0)	131.8 (6.2)
**Diastolic**	**1 (n=66)**	74.1 (7.9)	-	-
	**2 (n=19)**	84.2 (15.4)	79.6 (11.1)	-
	**3 (n=6)**	83.3 (12.3)	82.5 (10.9)	80.2 (9.7)
**Participants without diabetes reporting all BP readings (N=20; 12 (60.0%) had BP measured according to guidelines)**				
**Systolic**	**1 (n=12)**	135.8 (31.5)	-	-
	**2 (n=4)**	160.8 (22.5)	158.5 (19.4)	-
	**3 (n=4)**	154.5 (8.3)	152.3 (6.7)	144.3 (11.5)
**Diastolic**	**1 (n=12)**	75.3 (12.5)	-	-
	**2 (n=4)**	89.8 (8.9)	87.8 (10.8)	-
	**3 (n=4)**	89.0 (16.3)	87.0 (14.1)	84.0 (13.9)

Table S11: Likelihood of having BP measured multiple times or being asked to monitor BP at home, according to patient and practitioner characteristics in patients with and without diabetes, excluding possible duplicate submissions

	Likelihood of multiple BP measurements<br>(n (%) in each group)<br>(difference [95% confidence interval])	Likelihood of being asked to monitor BP at home<br>(n (%) in each group)<br>(difference [95% confidence interval])
**In patients with diabetes**		
**If the patient is hypertensive vs. normotensive**	45/98 (45.9%) vs. 27/74 (36.5%)<br>difference = 9.4% [-5.3 to 24.2%]	24/153 (15.7%) vs. 11/110 (10.0%)<br>difference = 5.7% [-2.4 to 13.7%]
**If the patient has treated hypertension vs. untreated hypertension**	39/88 (44.3%) vs. 6/10 (60.0%)<br>difference = 15.7% [-16.4 to 47.8%]	22/135 (16.3%) vs. 2/18 (11.1%)<br>difference = 5.2% [-10.6 to 21.0%]
**If BP was measured by a GP vs. a nurse**	16/36 (44.4%) vs. 54/130 (41.5%)<br>difference = 2.9% [-15.4 to 21.2%]	11/36 (30.6%) vs. 15/130 (11.5%)<br>difference = 19.0% [3.0 to 35.0%]
**In patients without diabetes**		
**If the patient is hypertensive vs. normotensive**	9/27 (33.3%) vs. 2/6 (33.3%)<br>difference = 0.0% [-41.7 to 41.7%]	14/40 (35.0%) vs. 0/14 (0.0%)<br>difference = 35.0% [20.2 to 49.8%]
**If the patient has treated hypertension vs. untreated hypertension**	8/22 (36.4%) vs. 1/5 (20.0%)<br>difference = 16.4% [-24.1 to 56.8%]	9/31 (29.0%) vs. 5/9 (55.6%)<br>difference = 26.5% [-9.7 to 62.7%]
**If BP was measured by a GP vs. a nurse**	9/21 (42.9%) vs 2/10 (20.0%)<br>difference = 22.9% [-9.7 to 55.5%]	7/21 (33.3%) vs 4/10 (40.0%)<br>difference = 6.7% [-29.8 to 43.1%]

For peer review only

STROBE 2007 (v4) Statement—Checklist of items that should be included in reports of *cross-sectional studies*

**Section/Topic**	**Item #**	**Recommendation**	**Reported on page #**
**Title and abstract**	1	(*a*) Indicate the study's design with a commonly used term in the title or the abstract	1
		(*b*) Provide in the abstract an informative and balanced summary of what was done and what was found	2
**Introduction**			
Background/rationale	2	Explain the scientific background and rationale for the investigation being reported	3
Objectives	3	State specific objectives, including any prespecified hypotheses	3
**Methods**			
Study design	4	Present key elements of study design early in the paper	4
Setting	5	Describe the setting, locations, and relevant dates, including periods of recruitment, exposure, follow-up, and data collection	4
Participants	6	(*a*) Give the eligibility criteria, and the sources and methods of selection of participants	4
Variables	7	Clearly define all outcomes, exposures, predictors, potential confounders, and effect modifiers. Give diagnostic criteria, if applicable	4
Data sources/ measurement	8*	For each variable of interest, give sources of data and details of methods of assessment (measurement). Describe comparability of assessment methods if there is more than one group	4/ Supplement
Bias	9	Describe any efforts to address potential sources of bias	*4*
Study size	10	Explain how the study size was arrived at	4
Quantitative variables	11	Explain how quantitative variables were handled in the analyses. If applicable, describe which groupings were chosen and why	4
Statistical methods	12	(*a*) Describe all statistical methods, including those used to control for confounding	4
		(*b*) Describe any methods used to examine subgroups and interactions	4
		(*c*) Explain how missing data were addressed	5
		(*d*) If applicable, describe analytical methods taking account of sampling strategy	N/A
		(*e*) Describe any sensitivity analyses	4
**Results**			

For peer review only

Participants	13*	(a) Report numbers of individuals at each stage of study—eg numbers potentially eligible, examined for eligibility, confirmed eligible, included in the study, completing follow-up, and analysed	5/ Figure 1
		(b) Give reasons for non-participation at each stage	Figure 1
		(c) Consider use of a flow diagram	Figure 1
Descriptive data	14*	(a) Give characteristics of study participants (eg demographic, clinical, social) and information on exposures and potential confounders	Table 1/ Table S1
		(b) Indicate number of participants with missing data for each variable of interest	5/Table 1/ S1
Outcome data	15*	Report numbers of outcome events or summary measures	5
Main results	16	(*a*) Give unadjusted estimates and, if applicable, confounder-adjusted estimates and their precision (eg, 95% confidence interval). Make clear which confounders were adjusted for and why they were included	5/ Table 2/ Figures 2 and 3
		(*b*) Report category boundaries when continuous variables were categorized	N/A
		(*c*) If relevant, consider translating estimates of relative risk into absolute risk for a meaningful time period	N/A
Other analyses	17	Report other analyses done—eg analyses of subgroups and interactions, and sensitivity analyses	5/Supplement
**Discussion**			
Key results	18	Summarise key results with reference to study objectives	6
Limitations	19	Discuss limitations of the study, taking into account sources of potential bias or imprecision. Discuss both direction and magnitude of any potential bias	6
Interpretation	20	Give a cautious overall interpretation of results considering objectives, limitations, multiplicity of analyses, results from similar studies, and other relevant evidence	7/8
Generalisability	21	Discuss the generalisability (external validity) of the study results	7
**Other information**			
Funding	22	Give the source of funding and the role of the funders for the present study and, if applicable, for the original study on which the present article is based	9

*Give information separately for cases and controls in case-control studies and, if applicable, for exposed and unexposed groups in cohort and cross-sectional studies.

Note: An Explanation and Elaboration article discusses each checklist item and gives methodological background and published examples of transparent reporting. The STROBE checklist is best used in conjunction with this article (freely available on the Web sites of PLoS Medicine at http://www.plosmedicine.org/, Annals of Internal Medicine at http://www.annals.org/, and Epidemiology at http://www.epidem.com/). Information on the STROBE Initiative is available at www.strobe-statement.org.

For peer review only
